# Supplementary material for: 8-Hydroxydaidzein Downregulates JAK/STAT, MMP, Oxidative Phosphorylation, and PI3K/AKT Pathways in K562 Cells
Source: Biomedicines. 2021 Dec 14;9(12):1907. doi: 10.3390/biomedicines9121907 (PMC8698423; doi:10.3390/biomedicines9121907)
Supplement: Supplementary file 1 [file biomedicines-09-01907-s001.zip › biomedicines-1476563-supplementary.pdf]

Supplementary materials:

Supplementary Table S1: Biological processes in the Gene Ontology (GO) analysis of up- and downregulated genes, associated with DEGs of 8-OHD-treated samples (H1 and H5).

Cluster	Ontology Name(Ontology-ID)	P Value	Gene symbols
**c1**	mitochondrion(GO:0005739)	1.81E-33	AADAT, AATK, ABCB10, ABCB6, ABCB7, ACAD9, ACADM, ACADS, ACAT1, ACOX3, ACP6, ACSM1, ACSM2B, ACSM3, ADCK2, ADH5, AGK, AGPS, AKR7A2, ALDH1B1, ALDH1L2, ALDH2, ALDH4A1, ALDH5A1, APOBEC3G, ARG2, ASB9, ATIC, ATP5D, ATP5G3, ATP5I, ATP5J2, ATP5L, AZI2, BCAT1, BCAT2, BID, C17orf89, C1QBP, CA5A, CASP8, CAT, CBR4, CCDC58, CECR5, CHCHD10, CHCHD6, CISD1, CKB, CLPP, CLYBL, COMT, COMTD1, COQ3, COQ4, COX20, CPOX, CTPS2, CYB5R2, CYBB, CYC1, D2HGDH, DCPS, DDIT4, DECR1, DGUOK, DHFRL1, DHODH, DIAPH2, DNAJC15, DNAJC19, DYNLL1, ECH1, ECHDC2, ECHDC3, ECI1, EFHD1, EHHADH, ENDOG, ETFDH, FASTKD1, FASTKD2, FDPS, FKBP4, G0S2, GALC, GBAS, GCAT, GFM2, GLYATL1, GLYCTK, GPT2, GSTK1, GUF1, H6PD, HAGH, HCCS, HDDC2, HEATR1, HINT2, HLCS, HOXB9, HRSP12, HSD17B8, HSDL2, HSPE1, IARS2, ICT1, IDH1, IMMP2L, KCNJ11, LACE1, LACTB2, LIAS, LIG3, LIN28B, LIPT2, LONP1, LRPPRC, LYRM4, LYRM5, LYRM7, MACROD1, MAPK12, MARS, MARS2, MDH2, ME2, MECR, METAP1D, MLYCD, MRM1, MRPL11, MRPL13, MRPL19, MRPL21, MRPL23, MRPL24, MRPL30, MRPL4, MRPL40, MRPL42, MRPL47, MRPL48, MRPS25, MTG1, MTHFD1L, MTIF2, MTO1, MTPAP, NARS,

			NARS2, NDUFA10, NDUFA3, NDUFAF2, NDUFAF3, NDUFB10, NDUFB8, NDUFS6, NDUFS7, NDUFV1, NIPSNAP1, NIPSNAP3A, NLN, NME1, NME4, NMNAT3, NNT, NT5C, NT5C3A, NT5M, NTHL1, NUBPL, NUDT8, OPA1, OXCT1, OXLD1, P4HA1, PCCA, PCCB, PCK2, PECR, PEMT, PET100, PHYH, PNKD, PNPT1, PON2, PPA2, PPOX, PRELID2, PSMC2, PTCD2, PTGES2, PTPN11, PTPRH, PTRH1, PXMP2, PYCR1, QDPR, QTRT1, RAB11A, RAB3D, RAD51C, RHBDD1, RHOT1, RMDN1, RRP15, RTN4IP1, SARS2, SDHB, SERHL2, SFXN2, SFXN4, SIRT5, SLC25A12, SLC25A21, SLC25A30, SLC25A32, SLC25A39, SLC25A41, SLC25A43, SLC35B3, SOD2, SORD, STAP1, STAR, SURF1, TARS2, TATDN3, TAZ, TBC1D15, TFB1M, TGM2, THEM4, TIMM10, TIMM13, TIMM8B, TMEM70, TMLHE, TRAK2, TRAP1, TRIT1, TRMT61B, TSPO, UNG, URI1, VARS, VHL, VWA8, ZADH2
**c1**	catalytic activity(GO:0003824)	2.58E-26	AADAT, AATK, ABCA3, ABCA8, ABCB10, ABCB6, ABCB7, ABCC4, ABHD16A, ABHD3, ACAD9, ACADM, ACADS, ACAT1, ACOX3, ACP6, ACSL3, ACSM1, ACSM2B, ACSM3, ADA, ADAM15, ADAMTS3, ADAMTSL4, ADAT2, ADCK2, ADCY10, ADCY3, ADH5, ADRBK1, ADRBK2, ADSS, AGK, AGL, AGPAT3, AGPS, AKAP7, AKR1C2, AKR7A2, AKT1, ALDH1A1, ALDH1B1, ALDH1L2, ALDH2, ALDH4A1, ALDH5A1, ALKBH2, ALKBH6, ALKBH8, ALOX12, ALOX5, ALPK1, ANAPC4, APEH, APH1B, APIP, APOBEC3D, APOBEC3G, APRT, APTX, ARG2, ARL2, ARL4A, ARSK, ASCC3, ASL, ASMTL, ASS1, ATAD2B, ATAD5, ATG10, ATG4C, ATHL1, ATIC, ATP11B, ATP11C, ATP2A1, ATP2A3, ATP5D, ATP5H, ATP5I, ATP5J2, ATP5L, ATP6V0E1, ATP7A, ATP8B3, AURKB, AZU1, B3GALNT1, B4GALT2, BCAT1, BCAT2, BIRC3, BLVRB, BMP2K, BMX, BPNT1, BRSK1, BTK, C3orf33, CA1, CA5A, CA8, CAMKK2, CARS, CASP8, CASS4, CAT, CBR4, CBS, CCNB1,

CCNE1, CDK10, CDKAL1, CDKL1, CECR5, CENPE, CERS4, CES2, CFD, CFI, CHKB, CHPT1, CKB, CLIC2, CLOCK, CLPP, CLYBL, CMAS, CMSS1, CNOT6, CNOT6L, COLGALT2, COMT, COMTD1, COQ3, CPA2, CPOX, CPSF2, CPZ, CSRP2BP, CTBS, CTPS2, CTSC, CTSS, CWC27, CYB5R2, CYBB, CYP2F1, CYP2R1, CYP4V2, D2HGDH, DARS, DCPS, DCTN2, DDX10, DDX43, DDX52, DDX60L, DECR1, DECR2, DEGS2, DGKA, DGUOK, DHDH, DHFRL1, DHODH, DHRS11, DHRS3, DIMT1, DIRAS3, DIS3L, DKC1, DLG3, DLGAP5, DMC1, DNA2, DNAJC10, DNMT3B, DNPEP, DNPH1, DPH5, DPM3, DPP7, DSTYK, DTD1, DTD2, DUS4L, DUSP19, DUSP9, DYNC2LI1, DYNLL1, DYRK4, DZIP3, EBPL, ECH1, ECHDC2, ECHDC3, ECI1, EEF1A2, EEF2K, EFEMP1, EHHADH, ELOVL6, EML2, ENDOG, ENGASE, ENO3, ENPP3, EPHX2, EPM2A, EPRS, ERAP1, ERCC4, ERCC8, ERMP1, ESD, ETFDH, EXOSC2, EXOSC5, F12, FAH, FAM135A, FAN1, FANCL, FANCM, FAR1, FASTKD1, FASTKD2, FBXO4, FBXO6, FDPS, FGFR3, FHIT, FIP1L1, FKBP4, FKBP7, FTSJ1, FUOM, FUT1, FUT8, G2E3, GAL3ST1, GALC, GALK1, GALK2, GALM, GALNT11, GALNT12, GALNT5, GART, GBA3, GCAT, GDPD1, GFM2, GGACT, GGPS1, GLYATL1, GLYCTK, GMPPA, GNB1, GOT1, GPT2, GSPT1, GSTA4, GSTCD, GSTK1, GSTO2, GTF2H1, GTF2H3, GTPBP2, GUF1, GXYLT2, GYG2, GYLTL1B, H6PD, HAGH, HAGHL, HBB, HBS1L, HCCS, HDAC10, HDDC2, HDDC3, HECW2, HEPH, HEXDC, HFM1, HINT2, HLCS, HLTF, HMBS, HNMT, HNRNPU, HRASLS, HRSP12, HS3ST3A1, HSD11B1L, HSD17B8, HSD3B7, HSDL2, HSPA5, IARS, IARS2, ICT1, IDH1, IFI30, IMMP2L, IMPA2, INPP1, INPP5J, INTS9, IRAK1, ISPD, ITPA, ITPKA, KCNH2, KDM1B, KDM4D, KHK, KIF13A, KIF14, KIF20A, KIF2A, KIT, KLHL13, KLK8, KSR1, L3HYPDH, LACTB2, LANCL3, LARS, LGALS13, LHPP, LIAS, LIG3, LIPT2, LONP1,

LPGAT1, LPL, LRR1, LYPLAL1, LYZ, MACROD1, MAN2A2, MAP3K13, MAP3K5, MAPK12, MARS, MARS2, MATK, MBOAT2, MBTPS2, MCM6, MDH2, ME2, MECR, MET, METAP1D, METTL10, METTL9, MGST2, MINA, MLYCD, MMP11, MMP14, MOCOS, MPP3, MRM1, MSH2, MSH5, MTG1, MTHFD1L, MTHFR, MTIF2, MTO1, MTPAP, MTR, MYO16, MYO6, MYO7A, N4BP2L2, NAAA, NAALAD2, NADSYN1, NARS, NARS2, NAT2, NAT8L, NAV1, NAV2, NDUFA10, NDUFA3, NDUFAF2, NDUFB10, NDUFB8, NDUFS6, NDUFS7, NDUFV1, NEIL3, NEK1, NEK3, NLN, NME1, NME4, NME7, NMNAT3, NMRK1, NMRK2, NMT2, NNT, NQO2, NSUN6, NT5C, NT5C3A, NT5M, NTHL1, NTRK1, NUDT12, NUDT14, NUDT16, NUDT17, NUDT22, NUDT7, NUDT8, OGFOD2, OGFOD3, OLA1, OPA1, OXCT1, P4HA1, PAFAH1B1, PAICS, PANK1, PAPLN, PAPPA, PBK, PBLD, PCCA, PCCB, PCK2, PCSK4, PCSK6, PCSK9, PECR, PEMT, PEX1, PFKL, PHGDH, PHKA2, PHKB, PHYH, PI4K2B, PIF1, PIGB, PIGK, PIGN, PIGT, PIGU, PIGZ, PIK3CA, PIK3CG, PIK3R3, PKDCC, PKLR, PKN3, PLAU, PLBD1, PLCD1, PLCL1, PLD1, PLOD3, PM20D2, PMS2, PNKD, PNPO, PNPT1, POLB, POLD2, POLH, POLR1B, POLR2E, POLR2I, POLR3C, POLR3E, POLR3G, POMGNT1, PON2, POP1, PPA1, PPA2, PPAPDC3, PPIA, PPID, PPIL1, PPIP5K2, PPM1A, PPM1B, PPM1E, PPM1J, PPM1M, PPOX, PPP2R5C, PPP3CB, PRKCH, PRKX, PRMT2, PRSS21, PRSS57, PSAT1, PSKH1, PSMC2, PSPH, PTGDS, PTGES2, PTGR2, PTP4A2, PTP4A3, PTPDC1, PTPN11, PTPN18, PTPRC, PTPRH, PTRH1, PUS7, PYCR1, PYGL, PYROXD1, QDPR, QPRT, QTRT1, RAB11A, RAB27A, RAB27B, RAB28, RAB3D, RAB40B, RAC3, RAD51B, RAD51C, RAG1, RAP2B, RASL10A, RBP3, RDH10, RERG, RHBDD1, RHBDL1, RHOBTB1, RHOT1, RNF130, RNF138, RNF213, RNF5, RNGTT, RPIA, RPS6KA3, RPS6KB2, RPUSD2, RTCA, RTEL1,

			RTN4IP1, RUVBL1, RUVBL2, SARS2, SCRN3, SDHB, SEC62, SENP7, SEPHS1, SEPHS2, SEPSECS, SERHL2, SETMAR, SGK494, SGMS1, SIGMAR1, SKIV2L2, SLC9B2, SMPDL3B, SMYD2, SOD2, SORD, SPATA20, SPCS2, ST3GAL4, ST6GALNAC1, ST8SIA4, ST8SIA6, STEAP1, STEAP3, STK16, STK24, STRADB, STT3B, SURF1, SYK, TALDO1, TARBP1, TARS2, TATDN3, TAZ, TFB1M, TFR2, TGM2, TGM3, THEM4, TKTL1, TMLHE, TMPRSS4, TMPRSS5, TMX1, TMX3, TNFAIP3, TPK1, TPST2, TRDMT1, TRIT1, TRMT11, TRMT1L, TRMT5, TRMT61B, TSTA3, TUBE1, TXNRD3, UBA5, UBE2E1, UBE2E2, UBE2L6, UBE2N, UGGT2, UNG, UNKL, USP15, USP18, USP33, USP44, USP45, USP47, USP5, USP9X, VARS, VHL, VRK1, VRK2, VWA8, WARS, WBSCR22, WDR4, XRCC6BP1, XRN1, YARS, YDJC, YES1, ZADH2, ZC3HAV1, ZDHHC19, ZDHHC2, ZMYND8, ZRANB3
**c1**	small molecule metabolic process(GO:0044281)	8.17E-21	AADAT, ABCB7, ACADM, ACADS, ACAT1, ACOX3, ACSL3, ACSM1, ACSM2B, ACSM3, ADA, ADCY10, ADCY3, ADH5, ADSS, AGL, AGPAT3, AGPS, AKR1C2, AKT1, ALDH1A1, ALDH1B1, ALDH1L2, ALDH2, ALDH4A1, ALDH5A1, ALKBH2, ALKBH8, ALOX12, ALOX5, APIP, APOBEC3G, APRT, ARG2, ARL2, ARL4A, ARSK, ARV1, ASL, ASS1, ATIC, ATP2A1, ATP5D, ATP5G3, ATP5H, ATP5I, ATP5J2, ATP5L, ATP7A, ATP8B3, B4GALT2, BCAT1, BCAT2, BLVRB, BPNT1, CA1, CA5A, CA8, CALCRL, CARS, CAT, CBR4, CBS, CERS4, CHCHD10, CHKB, CHPT1, CKB, CMAS, COMT, COQ3, COQ4, CPOX, CTCFL, CTPS2, CYB5R2, CYC1, CYP2F1, CYP2R1, CYP4V2, D2HGDH, DARS, DECR1, DECR2, DEGS2, DGUOK, DHDH, DHFRL1, DHODH, DHRS3, DIRAS3, DNA2, DNAJC10, DNMT3B, DNPH1, DRD4, DTD1, DTD2, DUS4L, EBPL, ECH1, ECHDC2, ECI1, EHHADH, ELOVL6, ENO3, ENPP3, EPHX2, EPRS, ESD, ETFDH, FABP5, FAH, FAR1, FDPS, FHIT, FTSJ1, FUOM, FUT1,

	FUT8, G0S2, GAL3ST1, GALC, GALK1, GALK2, GALM, GART, GBA3, GBAS, GCAT, GDPD1, GFM2, GGACT, GGPS1, GMPPA, GNB1, GOT1, GPC2, GPT2, GSPT1, GSTA4, GSTK1, GSTO2, GYG2, H6PD, HAGH, HAGHL, HBB, HLTF, HMBS, HMMR, HRASLS, HS3ST3A1, HSD17B8, HSD3B7, HSPA5, IARS, IARS2, IDH1, IGF1, IMPA2, INPP1, INPP5J, ITPA, ITPKA, KCNJ11, KDM1B, KDM4D, KHK, LARS, LEPR, LIAS, LIPT2, LONP1, LPGAT1, LPL, LRP5, LYRM4, MACROD1, MAN2A2, MARCKS, MARS, MARS2, MBOAT2, MBTPS2, MDH2, ME2, MECR, METTL10, MGST2, MLXIPL, MLYCD, MOCOS, MSH2, MTG1, MTHFD1L, MTHFR, MTO1, MTR, MYC, NADSYN1, NARS, NARS2, NAT2, NDUFA10, NDUFA3, NDUFB10, NDUFB8, NDUFS6, NDUFS7, NDUFV1, NFYB, NME1, NME4, NME7, NMNAT3, NMRK1, NMRK2, NNT, NOSTRIN, NR5A2, NSUN6, NT5C, NT5C3A, NT5M, NTHL1, NUDT12, NUDT16, NUDT7, NUP210, NUP88, OLA1, OPA1, OXCT1, P4HA1, PAFAH1B1, PAICS, PANK1, PCCA, PCCB, PCK2, PCSK9, PECR, PEMT, PEX7, PFKL, PHGDH, PHKA2, PHKB, PHYH, PI4K2B, PIK3CA, PIK3CG, PIK3R3, PKLR, PLBD1, PLCD1, PLD1, PMS2, PNKD, PNPO, PPA1, PPA2, PPIP5K2, PPOX, PRKAR2B, PRMT2, PSAT1, PSMC2, PSPH, PTGDS, PTGES2, PTGR2, PTPN11, PYCR1, PYGL, QDPR, QKI, QPRT, QTRT1, RAB11A, RAB27A, RAB27B, RAB28, RAB3D, RAB40B, RAC3, RAP2B, RASL10A, RDH10, RERG, RHAG, RHOBTB1, RHOT1, RNF213, RPIA, RTEL1, RUVBL2, SARS2, SDHB, SEPHS2, SEPSECS, SETMAR, SGMS1, SIGMAR1, SLC25A12, SLC25A21, SLC25A32, SLC2A3, SLC2A5, SLC35A3, SLC35D1, SLC37A4, SLC44A5, SLC7A5, SLCO1B1, SMYD2, SOD2, SORD, SPATA20, ST3GAL4, STAR, STAT5A, SURF1, SYK, TALDO1, TARS2, TAZ, TBL1X, TDRD5, TGM3, THEM4, TKTL1, TMLHE, TMX3, TPK1, TRDMT1, TRMT11, TRMT61B, TSTA3, TUBE1, VAPA, VARS, WARS, YARS

**c1**	mitochondrial part(GO:0044429)	1.57E-18	AADAT, ABCA8, ABCB10, ABCB6, ABCB7, ACADM, ACADS, ACAT1, ACSL3, ACSM1, ACSM2B, ACSM3, AGK, ALDH1B1, ALDH2, ALDH4A1, ALDH5A1, ANXA1, ARG2, ARL2, ATP5D, ATP5G3, ATP5H, ATP5I, ATP5J2, ATP5L, BCAT2, BID, C1QBP, CA5A, CASP8, CAT, CBR4, CHCHD5, CHCHD6, CISD1, CLPP, COA1, COQ3, COQ4, COX20, CPOX, CYC1, D2HGDH, DECR1, DGUOK, DHFRL1, DHODH, DNA2, DNAJC19, ECI1, EFHD1, ETFDH, GPT2, GRAMD4, GSTK1, GUF1, HAGH, HCCS, HSD17B8, HSPE1, IARS2, ICT1, IMMP2L, LONP1, LRPPRC, LYRM4, LYRM7, MARS2, MDH2, ME2, MRPL11, MRPL13, MRPL23, MRPL40, MRPL42, MRPL47, MRPL48, MRPS25, NARS2, NAT8L, NDUFA10, NDUFA3, NDUFAF3, NDUFB10, NDUFB8, NDUFS6, NDUFS7, NDUFV1, NIPSNAP1, NLN, NME4, NNT, NT5M, OPA1, OXCT1, PCCA, PCCB, PCK2, PEMT, PNPT1, PPA2, PPOX, PRKAR2B, PYCR1, RHOT1, RNF5, SARS2, SDHB, SFXN2, SFXN4, SIRT5, SLC25A12, SLC25A21, SLC25A30, SLC25A32, SLC25A39, SLC25A41, SLC25A43, SLC9B2, SOD2, SORD, STAR, SURF1, TARS2, TAZ, TDRD7, TFB1M, THEM4, TIMM10, TIMM13, TIMM8B, TMEM173, TMEM70, TMLHE, TRMT5, TSPO, TXNIP, VRK2
**c1**	primary metabolic process(GO:0044238)	5.67E-14	AADAT, AATK, ACADM, ACADS, ACAT1, ACOX3, ACP6, ACSL3, ACSM1, ACSM2B, ACSM3, ADA, ADAM15, ADAMTS3, ADAT2, ADCK2, ADCY10, ADCY3, ADH5, ADRBK1, ADRBK2, ADSS, AES, AFP, AGK, AGL, AGPAT3, AGPS, AJUBA, AKAP7, AKIRIN2, AKR1C2, AKR7A2, AKT1, ALDH1A1, ALDH1B1, ALDH2, ALDH4A1, ALDH5A1, ALKBH2, ALKBH8, ALOX12, ALOX5, ALPK1, ANAPC16, ANAPC4, ANP32A, ANXA1, APEH, APH1B, APIP, APOBEC3D, APOBEC3G, APOL4, APRT, APTX, ARG2, ARHGAP22, ARL2, ARL4A, ARSK, ARV1, ASB13, ASB9, ASCC3, ASF1A, ASL, ASS1, ASXL1, ATF5, ATF6, ATG10, ATG4C, ATHL1, ATIC, ATP2A1, ATP5D, ATP5G3, ATP5H,

ATP5I, ATP5J2, ATP5L, ATP7A, ATP8B3, AURKB, AZU1, B3GALNT1, B4GALT2, BCAT1, BCAT2, BLVRB, BMP2K, BMX, BOP1, BPNT1, BRSK1, BTK, C1QBP, CALCRL, CAMKK2, CAND2, CARS, CASP8, CAT, CBFA2T3, CBR4, CBS, CCNB1, CCNE1, CCT5, CDK10, CDKAL1, CDKL1, CEBPD, CELF4, CERS4, CFD, CFH, CFI, CHCHD10, CHKB, CHPT1, CIRBP, CIRH1A, CISH, CITED1, CKB, CLGN, CLPP, CLPSL2, CMAS, CNOT6, CNOT6L, COL4A2, COLGALT2, COMT, COQ3, CPA2, CPNE7, CPSF2, CPZ, CR2, CSRP2BP, CSTF2, CSTF3, CTBS, CTCFL, CTPS2, CTSC, CTSS, CWC27, CYB5R2, CYP2R1, CYP4V2, D2HGDH, DARS, DCAF4, DCPS, DEAF1, DECR1, DECR2, DEGS2, DEPDC1, DERL3, DGUOK, DHDH, DHFRL1, DHODH, DHRS3, DIMT1, DIRAS3, DIS3L, DKC1, DMC1, DNA2, DNAJC10, DNAJC19, DNAJC21, DNMT3B, DNPEP, DNPH1, DPH1, DPH5, DPM3, DPP7, DRD4, DSTYK, DTD1, DTD2, DUS4L, DUSP19, DUSP9, DYNLL1, DYRK4, DZIP3, E2F2, E2F5, EAF2, EBPL, ECH1, ECHDC2, ECI1, EDF1, EEF2K, EFEMP1, EFNA1, EHHADH, EIF4EBP1, ELOVL6, ENDOG, ENO3, ENPP3, EPHX2, EPM2A, EPRS, ERAP1, ERCC4, ERCC8, EREG, ERMP1, ESD, ESRRB, ETFDH, ETS1, ETV1, EXOSC2, EXOSC5, EXOSC8, F12, FABP5, FAH, FAM135A, FAM175A, FAN1, FANCC, FANCD2, FANCF, FANCL, FANCM, FAR1, FBXO4, FBXO6, FDPS, FGF2, FGFR3, FHIT, FIP1L1, FKBP4, FKBP7, FOXP1, FTSJ1, FUBP1, FUOM, FUS, FUT1, FUT8, FYB, G0S2, G2E3, GAL3ST1, GALC, GALK1, GALK2, GALM, GALNT11, GALNT12, GALNT5, GART, GATA1, GBA3, GBAS, GCAT, GCFC2, GDPD1, GFI1B, GFM2, GGACT, GGPS1, GIN1, GLYCTK, GMPPA, GNB1, GOT1, GPC2, GPT2, GSPT1, GSTA4, GSTCD, GSTK1, GTF2H1, GTF2H3, GUF1, GXYLT2, GYG2, H6PD, HAGH, HAGHL, HBS1L, HDAC10, HEATR1, HECW2, HES1, HES4, HES6, HEXDC, HEXIM2, HHEX, HINT2, HIST1H4C, HIST1H4H, HIST1H4I, HLCS,

	HLTF, HMBOX1, HMBS, HMG20B, HMGB1, HMGB3, HMMR, HNRNPD, HNRNPK, HNRNPU, HOXB7, HOXB9, HOXC4, HOXD8, HRASLS, HS3ST3A1, HSD17B8, HSD3B7, HSPA4L, HSPA5, HSPE1, IARS, IARS2, IDH1, IFT88, IGF1, IGF2BP3, IMMP2L, IMPA2, INPP1, INPP5J, INTS10, INTS9, IRAK1, IRAK1BP1, IRX5, ISPD, ITPA, ITPKA, JAG2, JKAMP, KANK1, KCNJ11, KDM1B, KDM4D, KHK, KIAA0368, KIAA1429, KIT, KLF1, KLF10, KLF3, KLHL13, KLK8, KSR1, L3MBTL1, LARS, LEPR, LGALS13, LHPP, LHX3, LIAS, LIG3, LIN28B, LIPT2, LMAN1, LONP1, LPGAT1, LPL, LRP5, LRP8, LRPPRC, LRR1, LSM5, LYPLAL1, MACC1, MACROD1, MAGOHB, MAMDC2, MAN2A2, MAP3K13, MAP3K5, MAPK12, MARS, MARS2, MATK, MAZ, MBOAT2, MBTPS2, MCM6, MDH2, MECOM, MECR, MEF2C, MEIS2, MESP1, MET, METAP1D, METTL10, MGST2, MID1IP1, MLF1, MLXIPL, MLYCD, MMP11, MMP14, MNX1, MOB1B, MPHOSPH6, MRM1, MRPL11, MRPL13, MRPL19, MRPL21, MRPL23, MRPL24, MRPL4, MRPL42, MRPL47, MRPS25, MSH2, MSH5, MTG1, MTHFR, MTO1, MTPAP, MTR, MUC19, MUM1, MURC, MYB, MYC, NAAA, NAALAD2, NADSYN1, NARS, NARS2, NASP, NDUFA10, NDUFS6, NEIL3, NEK1, NEK3, NFE2, NFIB, NFYB, NHLH1, NLN, NME1, NME4, NME7, NMNAT3, NMRK1, NMRK2, NMT2, NPM3, NR2C1, NR5A2, NT5C, NT5C3A, NT5M, NTHL1, NTRK1, NUDT12, NUDT16, NUDT7, NUP210, NUP88, OLA1, OPA1, ORC2, OXCT1, P4HA1, PAFAH1B1, PAICS, PANK1, PAPPA, PBK, PBX2, PCCA, PCCB, PCK2, PCOLCE, PCSK4, PCSK6, PCSK9, PECR, PEMT, PEX1, PEX7, PFKL, PGR, PHF10, PHF11, PHF19, PHGDH, PHKA2, PHKB, PHTF1, PHTF2, PHYH, PI4K2B, PIGB, PIGK, PIGN, PIGT, PIGU, PIGZ, PIK3CA, PIK3CG, PIK3R3, PKDCC, PKLR, PKN3, PLAU, PLBD1, PLCD1, PLCL1, PLD1, PLOD3, PLSCR1, PMS2, PNKD, PNPT1, POLB, POLD2, POLH, POLR1B, POLR2E,

POLR2I, POLR3C, POLR3E, POLR3G, POLR3GL, POMGNT1, POP1, POT1, POU3F4, PPA1, PPA2, PPIA, PPID, PPIL1, PPIP5K2, PPM1A, PPM1B, PPM1J, PPM1M, PPP2R5C, PPP3CB, PRKAR2B, PRKCH, PRKX, PRMT2, PRR13, PRSS21, PRSS57, PSAT1, PSIP1, PSKH1, PSMC2, PSPC1, PSPH, PTCD2, PTGDS, PTGER3, PTGES2, PTGR2, PTP4A2, PTP4A3, PTPDC1, PTPN11, PTPN18, PTPRC, PTPRH, PUS7, PYCR1, PYGL, QDPR, QKI, QPRT, QTRT1, RAB11A, RAB27A, RAB27B, RAB28, RAB3D, RAB40B, RAC3, RAD51AP1, RAD51B, RAD51C, RAG1, RAP2B, RASL10A, RBMX, RBP3, RCOR2, RDH10, RDM1, RERG, RFX5, RFXANK, RGCC, RHBDD1, RHBDL1, RHOBTB1, RHOT1, RNASEH2B, RNF138, RNF213, RNF5, RNGTT, RPIA, RPL17, RPL26, RPL34, RPL41, RPS19, RPS6KA3, RPS6KB2, RPUSD2, RTCA, RTEL1, RUNX1, RUNX2, RUVBL1, RUVBL2, SAP18, SARS2, SCRN3, SENP7, SEPHS1, SEPHS2, SEPSECS, SETMAR, SFPQ, SGK494, SGMS1, SIGMAR1, SIRT5, SIX5, SKIV2L2, SLC25A12, SLC25A21, SLC25A32, SLC2A3, SLC2A5, SLC35A3, SLC35D1, SLC37A4, SLC44A5, SLC7A5, SLCO1B1, SMC6, SMEK2, SMPDL3B, SMYD2, SNAPC5, SNRPD1, SOCS2, SOCS3, SOD2, SORD, SOX18, SOX5, SOX6, SP2, SPATA20, SPCS2, SRA1, SRSF10, SRSF7, SSX5, ST3GAL4, ST6GALNAC1, ST8SIA4, ST8SIA6, STAR, STAT3, STAT5A, STK16, STK24, STRADB, STT3B, SURF1, SYK, SYNCRIP, TAF4B, TAF7L, TALDO1, TARBP1, TARS2, TAZ, TBL1X, TCEA1, TCEA2, TCEANC2, TCF19, TCFL5, TDRD5, TEAD4, TEX11, TFB1M, TFR2, TGM2, TGM3, THEM4, THOC2, THOC7, THRB, TIMM10, TIMM13, TIMM8B, TKTL1, TMEM165, TMEM67, TMLHE, TMPRSS4, TMPRSS5, TMX1, TNFAIP3, TPD52L1, TPST2, TRA2B, TRAK2, TRAP1, TRDMT1, TRIT1, TRMT11, TRMT1L, TRMT61B, TSEN15, TSPO, TSR3, TSTA3, TTC5, TUBE1, TXNIP, UBA5, UBAC1, UBE2E1, UBE2E2, UBE2L6, UBE2N, UGGT2, UNG, UNKL, URI1, USP15, USP18,

			USP33, USP44, USP45, USP47, USP5, USP9X, UTP11L, UTP18, UVSSA, VAPA, VARS, VEGFA, VHL, VPS28, VPS36, VRK1, VRK2, WARS, WDHD1, WDR12, WDR33, WDR36, WDR4, WDR61, WDR74, WNT10B, WT1, XRCC6BP1, XRN1, YARS, YDJC, YES1, ZDHHC2, ZFC3H1, ZIM2, ZMYND11, ZNF175, ZNF239, ZNF25, ZNF30, ZNF311, ZNF467, ZNF558, ZNF566, ZNF573, ZNF654, ZNF674, ZNF711, ZNF783, ZNF789, ZRANB3
**c1**	heterocycle metabolic process(GO:0046483)	2.58E-10	AADAT, ABCB6, ADA, ADCY10, ADSS, ALDH1L2, ALDH4A1, APIP, APOBEC3G, APRT, ARL2, ARL4A, ATIC, ATP2A1, ATP5D, ATP5G3, ATP5H, ATP5I, ATP5J2, ATP5L, ATP7A, ATP8B3, BLVRB, BPNT1, CALCRL, CAT, CHCHD10, CPOX, CTPS2, DGUOK, DHFRL1, DHODH, DIRAS3, DNA2, DUS4L, FHIT, GART, GBAS, GFM2, GNB1, GSPT1, H6PD, HLTF, HMBS, HSPA5, IDH1, ITPA, LIAS, LIPT2, LONP1, MACROD1, MCM6, MDH2, MOCOS, MSH2, MTG1, MTHFD1L, MTHFR, MTO1, MTR, NADSYN1, NME1, NME4, NME7, NMNAT3, NMRK1, NMRK2, NT5C, NT5C3A, NT5M, NTHL1, NUDT12, NUDT16, NUDT7, OLA1, OPA1, P4HA1, PAICS, PANK1, PKLR, PMS2, PNPO, PPOX, PSAT1, PSMC2, PYCR1, PYGL, QDPR, QPRT, QTRT1, RAB11A, RAB27A, RAB27B, RAB28, RAB3D, RAB40B, RAC3, RAP2B, RASL10A, RERG, RHOBTB1, RHOT1, RNF213, RPIA, RUVBL2, SLC25A32, SLC25A39, SPTA1, STAR, STAT5A, SURF1, TALDO1, TGM3, TKTL1, TPK1, TRMT61B, TSPO, TUBE1
**c1**	cellular nitrogen compound biosynthetic process(GO:0044271)	4.79E-10	ABCB6, ACADM, ADA, ADCY10, ADCY3, ADSS, AKT1, ALDH4A1, APIP, APRT, ARG2, ASL, ASS1, ATIC, ATP5D, ATP5G3, ATP5H, ATP5I, ATP5J2, ATP5L, ATP7A, BCAT1, BCAT2, CALCRL, CBS, CHCHD10, CHKB, CPOX, CTPS2, DGUOK, DHFRL1, DHODH, DUS4L, GART, GBAS, GOT1, GPT2, HAGH, HAGHL, HMBS, MCM6, MGST2, MOCOS, MTHFD1L, MTHFR, MTR,

			NADSYN1, NME1, NME4, NME7, NMNAT3, NMRK1, NMRK2, PAICS, PHGDH, PKLR, PNKD, PPOX, PSAT1, PSPH, PYCR1, QDPR, QPRT, QTRT1, SEPHS2, SLC25A39, SPTA1, SURF1, TMLHE, TPK1, TSPO
**c1**	nucleotide binding(GO:0000166)	8.14E-10	AATK, ABCA3, ABCA8, ABCB10, ABCB6, ABCB7, ABCC4, ACSL3, ACSM1, ACSM2B, ACSM3, ACTR3B, ADCK2, ADCY10, ADCY3, ADRBK1, ADRBK2, ADSS, AGK, AKAP7, AKT1, ALKBH8, ALPK1, APRT, ARL15, ARL2, ARL4A, ASCC3, ASS1, ATAD2B, ATAD5, ATP11B, ATP11C, ATP2A1, ATP2A3, ATP5D, ATP7A, ATP8B3, AURKB, BMP2K, BMX, BRSK1, BTK, CAMKK2, CARS, CAT, CBR4, CCDC18, CCT5, CDK10, CDKL1, CELF4, CENPE, CHKB, CIRBP, CKB, CLCN3, CLPP, CMSS1, CNTRL, CSTF2, CTPS2, DACH1, DARS, DDX10, DDX43, DDX52, DDX60L, DECR1, DGKA, DGUOK, DHFRL1, DHODH, DHRS3, DIRAS3, DMC1, DNA2, DSTYK, DUS4L, DYRK4, EEF1A2, EEF2K, EPRS, FANCM, FGFR3, FHIT, FIP1L1, FKBP4, FUS, GALK1, GALK2, GART, GFM2, GLYCTK, GMPPA, GSPT1, GTPBP2, GUF1, H6PD, HBS1L, HFM1, HINT2, HLCS, HLTF, HNRNPD, HNRNPU, HSPA4L, HSPA5, HSPE1, IARS, IARS2, IDH1, IGF2BP3, IRAK1, ITPA, ITPKA, KCNJ11, KHK, KIF13A, KIF14, KIF20A, KIF2A, KIT, KSR1, LACE1, LARS, LIG3, LONP1, MAP3K13, MAP3K5, MAPK12, MARS, MARS2, MATK, MAZ, MCM6, ME2, MET, MRPL23, MSH2, MSH5, MTG1, MTHFD1L, MTIF2, MTPAP, MYO16, MYO6, MYO7A, N4BP2L2, NADSYN1, NARS, NARS2, NAV1, NAV2, NDUFA10, NDUFV1, NEK1, NEK3, NME1, NME4, NME7, NMNAT3, NMRK1, NMRK2, NNT, NT5C, NT5C3A, NT5M, NTRK1, NUBPL, NUDT16, NXF5, OLA1, OPA1, OSBP2, PABPC1L, PAICS, PANK1, PBK, PCCA, PCCB, PCK2, PDS5B, PEX1, PFKL, PHGDH, PI4K2B, PIF1, PIK3CA, PIK3CG, PKDCC, PKLR, PKN3, PMS2, PNPO, PPIP5K2, PRKAR2B, PRKCH, PRKX, PSKH1, PSMC2, PSPC1, PYCR1, PYGL, QDPR,

			RAB11A, RAB27A, RAB27B, RAB28, RAB3D, RAB40B, RABL6, RAC3, RAD51B, RAD51C, RAP2B, RASL10A, RBM12, RBM41, RBMX, RDM1, RERG, RHOBTB1, RHOT1, RNF213, RNGTT, RPS6KA3, RPS6KB2, RTCA, RTEL1, RUNX1, RUNX2, RUVBL1, RUVBL2, SARS2, SEC62, SEPHS1, SEPHS2, SFPQ, SGK494, SIRT5, SKIV2L2, SLC22A5, SMC6, SORD, SRSF10, SRSF7, STK16, STK24, STRADB, SYK, SYNCRIP, TAF15, TARS2, TGM2, TGM3, TIA1, TMEM173, TPK1, TRA2B, TRAP1, TRIT1, TUBE1, TXNRD3, UBA5, UBE2E1, UBE2E2, UBE2N, VARS, VRK1, VRK2, VWA8, WARS, YARS, YES1, ZRANB3
**c1**	cofactor binding(GO:0048037)	4.43E-08	AADAT, ACAD9, ACADM, ACADS, ACAT1, ACBD4, ACCS, ACOX3, AGPS, BLVRB, CAT, CBR4, CBS, CYBB, D2HGDH, DECR1, DHFRL1, DHODH, DHRS11, DUS4L, EHHADH, ETFDH, FAR1, GCAT, GOT1, GPT2, H6PD, HSD3B7, IDH1, KDM1B, ME2, MOCOS, MTO1, NDUFS7, NDUFV1, NNT, PECR, PHGDH, PHYH, PNPO, PPOX, PSAT1, PYCR1, PYGL, QDPR, SDHB, SEPSECS, SIRT5, SORD, TSTA3, TXNRD3, UBA5
**c1**	oxidation-reduction process(GO:0055114)	5.48E-08	ACAD9, ACADM, ACADS, ACOX3, ACSM1, ADCY3, ADH5, AGL, AGPS, AKR1C2, AKT1, ALDH1A1, ALDH1B1, ALDH1L2, ALDH2, ALDH4A1, ALDH5A1, ALKBH2, ALKBH6, ALKBH8, ALOX12, ALOX5, ATP5D, ATP5H, ATP5I, ATP5J2, ATP5L, BLVRB, CAT, CPOX, CYB5R2, CYBB, CYC1, CYP2F1, CYP2R1, CYP4V2, D2HGDH, DECR1, DEGS2, DHFRL1, DHODH, DUS4L, ECH1, ECI1, EHHADH, EPM2A, ETFDH, FASTKD1, FASTKD2, GNB1, GSTO2, GYG2, H6PD, HCCS, HEPH, HSD3B7, IDH1, KCNJ11, KDM1B, LEPR, MARCKS, MDH2, ME2, MECR, MLXIPL, MLYCD, MTHFD1L, MTHFR, MTO1, MYC, NDUFA10, NDUFA3, NDUFB10, NDUFB8, NDUFS6, NDUFS7, NDUFV1, NNT, OGFOD2, OGFOD3, P4HA1, PCCA, PCCB, PECR, PEX7, PHGDH, PHKA2, PHKB, PHYH, PKLR, PLOD3, PNPO, PPOX, PRKAR2B, PTGR2,

			PYCR1, PYGL, PYROXD1, RPIA, RTN4IP1, SDHB, SOD2, SORD, STEAP1, STEAP3, SURF1, TALDO1, TAZ, TMLHE, TMX1, TXNRD3, ZADH2
**c1**	cytoplasm(GO:0005737)	1.38E-06	ABCB7, ACP6, ACTR3B, ACTR6, ADA, ADCY10, ADCY3, ADH5, ADRBK1, ADSS, AFP, AGL, AHCTF1, AKAP7, AKR1C2, AKT1, ALDH1A1, ALDH1L2, ALKBH6, ALKBH8, ALOX5, ANAPC16, ANP32A, ANP32E, ANXA1, ANXA3, APEH, APIP, APOBEC3D, APOBEC3G, APRT, APTX, ARFGEF1, ARHGAP4, ARL15, ARL2, ARL4A, ARVCF, ASAP3, ASB9, ASCC3, ASL, ASMTL, ASS1, ATF5, ATG10, ATG16L2, ATG4C, ATRN, AZI2, BAIAP2, BBIP1, BCAS1, BCAT1, BEX2, BID, BIRC3, BIVM, BLVRB, BRSK1, BSPRY, BST2, BTK, C1QBP, CA1, CA8, CALB1, CAMKK2, CARD8, CARD9, CARS, CASKIN1, CASP8, CASS4, CAV2, CBS, CC2D2A, CCNB1, CCT5, CDKL1, CELF4, CENPE, CEP70, CETN3, CHKB, CHP2, CIRBP, CITED1, CLIC2, CLOCK, CNKSR3, CNOT6L, COQ3, CPOX, CRIP3, CRLF1, CSRP2BP, CTCFL, CTHRC1, CUEDC2, CYBB, CYC1, DACH1, DARS, DCPS, DCTN2, DDIT4, DEAF1, DECR1, DGUOK, DIAPH2, DIS3L, DKC1, DLG3, DLGAP5, DNAAF3, DNPEP, DNPH1, DPH1, DRD4, DSTYK, DTD1, DTD2, DUSP19, DUSP9, DYNLL1, DZIP3, E2F5, EDF1, EEF1A2, EEF2K, EHBP1, EHHADH, EIF4EBP1, EML2, EML3, ENGASE, ENO3, EPB41L2, EPHX2, EPM2A, EPRS, ERAP1, ESD, ETS1, EXOSC2, EXOSC5, EXOSC8, FABP5, FADD, FAIM, FAM114A1, FAM129A, FANCC, FANCL, FBXL20, FBXO4, FBXO6, FDPS, FEZ1, FGF2, FHIT, FKBP4, FOXA2, FOXP1, FTSJ1, FUS, G2E3, GALK1, GALK2, GALM, GART, GDPD1, GLYCTK, GOT1, GPRASP1, GPSM3, GRAP2, GSTCD, GSTO2, H6PD, HAGH, HAMP, HDAC10, HECW2, HES1, HEXDC, HEXIM2, HHEX, HLCS, HMBOX1, HMGB1, HMMR, HNMT, HNRNPK, HOMER1, HOXB7, HRASLS, HRSP12, HSPA4L, HSPB3, HSPE1, IARS, IARS2, ICA1, IDH1, IFT43, IFT46, IGF2BP3, IMPA2, INPP5J, INTS10,

INTU, IRAK1BP1, ITPA, KANK1, KCNH2, KHK, KIAA1524, KIF14, KIT, KLHL5, KLK8, KNSTRN, LARS, LCN2, LHPP, LIN28B, LIPT2, LONP1, LPGAT1, LRPPRC, LRRCC1, LYPLAL1, LZTFL1, MACC1, MAGED1, MAPK12, MARCKS, MARS, MARS2, MARVELD1, MATK, MAZ, MBTPS2, MCOLN1, MEF2C, MEIOB, METTL10, MICALL2, MID1IP1, MINA, MLF1, MLXIPL, MLYCD, MMP14, MOB1B, MPHOSPH6, MRPS25, MS4A3, MTL5, MTPAP, MTR, MTRNR2L4, MTRNR2L8, MTRNR2L9, MURC, MVB12A, MYC, MYO16, MYO6, MYO7A, NAAA, NARS, NARS2, NASP, NAT8L, NAV1, NBAS, NCS1, NEDD9, NEK1, NEK3, NFE2, NME1, NMT2, NOXA1, NQO2, NR5A2, NSMF, NT5C, NT5C3A, NTRK1, NUDT12, NUDT14, NUDT16, NXF5, OLA1, PACSIN3, PAFAH1B1, PCM1, PCSK9, PDCD2, PDCD2L, PEX1, PFKL, PGR, PI4K2B, PIGT, PIK3CG, PLCD1, PLCL1, PNPT1, POLB, POLH, POLR1B, PPA1, PPA2, PPFIA3, PPHLN1, PPID, PPM1E, PPP1R14C, PPP1R42, PPP2R3C, PRC1, PRKAR2B, PRKCH, PRKX, PRMT2, PRSS21, PSMC2, PSPC1, PSPH, PTGR2, PTP4A2, PTPN11, PTPN18, PTPRH, PXMP2, PYCR1, PYGL, QDPR, QKI, QPRT, QTRT1, RAB11A, RAB28, RAB3D, RABL6, RAD51C, RALGPS2, RASA1, RASGRP4, RASSF5, RBP5, RCAN1, RDH10, RDM1, RERG, RFXANK, RGCC, RNF130, RNF213, RPS19, RPS6KB2, RPTOR, RUNX2, RUVBL1, RUVBL2, S100P, SAMSN1, SAP18, SARS2, SCIN, SDCCAG3, SDPR, SEPSECS, SERPINB6, SERPINB9, SFPQ, SGCG, SH3BGRL, SH3GL3, SIX5, SMAP2, SMEK2, SMIM19, SMYD2, SNRPD1, SNTA1, SOCS2, SOD2, SPA17, SPAG6, SPG11, SPRED1, SRA1, SRI, SRSF10, STAP1, STAT3, STAT5A, STK24, STRADB, STRBP, SYK, SYNCRIP, TAF15, TAF4B, TAF7L, TALDO1, TARS2, TBC1D15, TDRD7, TFR2, TGM2, TGM3, THEMIS2, THOC7, TIA1, TKTL1, TLE6, TMCO6, TMUB1, TNFAIP3, TNFRSF8, TNNI3, TPD52L1, TPK1, TPM1, TRAK2, TRIM14, TRIM7,

			TSC22D3, TSTA3, TTC25, TTC5, TXNIP, UBA5, UBAC1, UBE2N, UNKL, URI1, USP15, USP33, USP47, USP9X, UTP11L, UTRN, VARS, VEGFA, VPS28, VRK1, VRK2, WARS, WDHD1, WDR11, WDR18, WDR34, WDR4, WDR61, WIPF3, WT1, XRN1, YARS, YES1, ZC3HAV1, ZMYND11, ZNF175, ZP3
**c1**	cytosol(GO:0005829)	3.35E-06	ADA, ADCY10, ADRBK1, ADSS, AGL, AHCTF1, AJUBA, AKAP7, AKT1, ALDH1A1, ALKBH8, ALOX12, ALOX5, ANAPC4, ANO7, AP1S3, APIP, APOBEC3G, APRT, ARFGEF1, ARHGAP19, ARHGAP22, ARHGAP28, ARHGAP4, ARHGEF2, ARHGEF25, ASL, ASPSCR1, ASS1, ATG4C, ATIC, ATP7A, AURKB, BAIAP2, BCAT1, BID, BIRC3, BLVRB, BMX, BPNT1, BTK, C1QBP, C21orf59, CA1, CALB1, CARS, CASP8, CAT, CAV2, CBS, CCNB1, CCND2, CCNE1, CCT5, CENPE, CENPK, CEP70, CHAC1, CHKB, CISH, CITED1, CKB, CLOCK, CNOT6, CNOT6L, CNTRL, COG5, COMT, CORO7, CPLX1, CTPS2, DARS, DCPS, DCTN2, DDIT4, DENND4C, DES, DGKA, DGUOK, DIAPH2, DOCK8, DPP7, DUSP9, DYNC2LI1, DYNLL1, EEF2K, EHHADH, EIF4EBP1, ENGASE, ENO3, EPHX2, EPM2A, EPRS, ERAP1, EXOSC2, EXOSC5, EXOSC8, FADD, FAH, FANCC, FDPS, FGF2, FHIT, FKBP4, FYB, GALK1, GART, GBA3, GGPS1, GOT1, GRAP2, GSTA4, GSTO2, GYG2, HBB, HBD, HLCS, HMBS, HNRNPD, IARS, ICA1, IDH1, IGF2BP3, IMPA2, INPP1, INPP5J, IRAK1, ITPA, ITPKA, KHK, KIF2A, LARS, LCN2, LCP2, LHPP, LSM5, LYPLAL1, MAPK12, MARS, MATK, MID1IP1, MINA, MLXIPL, MLYCD, MOB1B, MOCOS, MTHFR, MTR, MYL4, MYO16, MYO6, MYO7A, NADSYN1, NARS, NAT2, NCS1, NIPSNAP3A, NME1, NMNAT3, NMT2, NT5C, NT5C3A, NUP88, PAFAH1B1, PAICS, PANK1, PARD6G, PCM1, PDS5B, PEX1, PEX7, PFKL, PHGDH, PHKA2, PHKB, PI4K2B, PIK3CA, PIK3CG, PIK3R3, PKLR, PLBD1, PLCD1, PLSCR1, PNPO, POMP, PPA1, PPIA, PPIP5K2, PPM1A,

			PPM1B, PPP3CB, PRKAR2B, PRKCH, PRMT2, PSAT1, PSIP1, PSMC2, PSPH, PTGES2, PTPN11, PYGL, QDPR, QPRT, RAC3, RAP2B, RASA1, RERG, RHOBTB1, RHOT1, RPIA, RPL17, RPL26, RPL34, RPL41, RPS19, RPS6KA3, RPTOR, SAMSN1, SDPR, SELENBP1, SERPINB6, SERPINB9, SH3BGR, SLC7A5, SMYD2, SNRPD1, SOCS2, SOCS3, SOS1, SPC24, SPC25, SPG11, SPTA1, SPTBN2, SRI, STAR, STAT3, STAT5A, STK24, STRADB, SYK, TALDO1, THEM4, TMEM173, TNFAIP3, TNNI3, TPK1, TPM1, TSC22D3, TUBGCP5, TXNIP, UBE2E1, UBE2L6, UBE2N, USP18, USP9X, VARS, VHL, VPS28, VPS36, VRK1, WARS, WIPF3, XRN1, YARS, YES1
**c1**	organelle inner membrane(GO:0019866)	6.52E-06	ABCA8, ABCB10, ABCB7, ACAT1, ATP11B, ATP5D, ATP5H, ATP5I, ATP5J2, ATP5L, CHCHD6, CPOX, CYC1, DHFRL1, DHODH, DNAJC19, ECI1, EFHD1, ETFDH, GSTK1, GUF1, HCCS, LMNB1, LRPPRC, MDH2, NDUFA10, NDUFA3, NDUFAF3, NDUFB10, NDUFB8, NDUFS6, NDUFS7, NDUFV1, NIPSNAP1, NME4, NNT, OPA1, PRKAR2B, SDHB, SFXN2, SIGMAR1, SLC25A12, SLC25A21, SLC25A30, SLC25A32, SLC25A39, SLC25A41, SLC25A43, SLC9B2, SOD2, TAZ, THEM4, TIMM10, TIMM13, TIMM8B, TMEM70, UNC50
**c1**	peroxisome(GO:0005777)	1.25E-05	ACOX3, AGPS, CAT, DECR2, ECH1, EHHADH, EPHX2, FAR1, GSTK1, HRASLS, HSDL2, IDH1, MLYCD, NUDT12, NUDT7, PECR, PEX1, PEX7, PHYH, SERHL2, TMEM173, ZADH2
**c1**	**single-stranded DNA binding(GO:0003697)**	5.12E-05	DMC1, ERCC4, FUBP1, HMGB1, HNRNPK, LONP1, LRPPRC, MCM6, MSH2, NEIL3, NME1, PMS2, POT1, RAD51AP1, RAD51B, SSBP4
**c1**	cofactor metabolic process(GO:0051186)	5.69E-05	AADAT, ABCB6, ALDH1L2, APIP, ATIC, BLVRB, COQ3, COQ4, CPOX, DHFRL1, ELOVL6, FAR1, H6PD, HMBS, IDH1, LIAS, LIPT2, MCM6, MDH2, MLYCD, MOCOS, MTHFD1L, MTHFR, NADSYN1, NMNAT3, NMRK1, NMRK2, NNT, NUDT12, NUDT7, PANK1, PNPO, PPOX, QDPR, QPRT, RPIA,

			SDHB, SLC25A32, SLC25A39, SPTA1, TALDO1, TPK1, TSPO
**c1**	**vitamin binding(GO:0019842)**	6.16E-05	AADAT, ACCS, CALB1, CBS, CD320, GCAT, GOT1, GPT2, HLCS, IRX5, MOCOS, MTR, OGFOD2, OGFOD3, P4HA1, PCCA, PHYH, PLOD3, PSAT1, PYGL, RBP3, RBP5, SEPSECS, SLC2A3, TMLHE
**c1**	magnesium ion binding(GO:0000287)	6.30E-05	ADCY10, ADSS, ATP11B, ATP11C, ATP8B3, BPNT1, BRSK1, COMT, ENO3, EPHX2, FAN1, IDH1, LHPP, MAP3K5, MAPK12, MSH2, MTPAP, NME1, NT5C3A, NUDT16, NUDT7, OPA1, PIF1, PKLR, PPA1, PPA2, PPM1A, PPM1B, PSPH, RPS6KA3, S100P, TGM3
**c1**	**cellular aromatic compound metabolic process(GO:0006725)**	6.54E-05	AADAT, ACSM1, ADA, ADSS, ALDH1L2, APRT, ATIC, ATP7A, CAT, CLYBL, COMT, CPOX, CYP2F1, DGUOK, DHFRL1, DHODH, DRD4, EPHX2, FAH, GART, HMBS, MOCOS, MTHFD1L, MTHFR, MTR, NT5C, NT5C3A, NT5M, PAICS, PON2, PPOX, QDPR, SLC25A32, STAR, TKTL1, TPK1
**c1**	response to interferon-beta(GO:0035456)	2.15E-04	BST2, IFITM1, IFITM2, PLSCR1, PNPT1, TMEM173
**c1**	**4 iron, 4 sulfur cluster binding(GO:0051539)**	3.48E-04	CDKAL1, DNA2, ETFDH, LIAS, NDUFS7, NDUFV1, NTHL1, NUBPL, RTEL1, SDHB
**c1**	exosome (RNase complex)(GO:0000178)	6.84E-04	DIS3L, EXOSC2, EXOSC5, EXOSC8, MPHOSPH6, PNPT1, SKIV2L2
**c1**	**organophosphate metabolic process(GO:0019637)**	8.27E-04	AGPAT3, AJUBA, ALDH5A1, BPNT1, CHKB, CHPT1, DPM3, FABP5, FAM135A, FAR1, FDPS, FGF2, GGPS1, IMPA2, INPP1, INPP5J, ITPKA, LGALS13, LPGAT1, LPL, MBOAT2, NUDT12, NUDT7, PAFAH1B1, PCSK9, PEMT, PI4K2B, PIGB, PIGN, PIGU, PIGZ, PIK3CA, PIK3CG, PIK3R3, PLBD1, PLCD1, PLD1, PLSCR1, PPIP5K2, PYCR1, SGMS1, SLC44A5, SMPDL3B, TALDO1, TAZ
**c1**	nucleoplasm(GO:0005654)	1.04E-03	AHCTF1, AKT1, ALKBH2, ANAPC4, ANP32A, APTX, ATF6, BOP1, CBFA2T3,

			CCNB1, CCNE1, CENPK, CIRBP, CPSF2, CSTF2, CSTF3, DKC1, DNA2, E2F2, E2F5, EAF2, ERCC4, ERCC8, ESRRB, FANCC, FANCD2, FANCF, FANCL, FANCM, FUS, GATA1, GTF2H1, GTF2H3, H3F3B, HDAC10, HEMGN, HES1, HIST1H4C, HIST1H4H, HIST1H4I, HMG20B, HMGB1, HNRNPD, HNRNPK, HNRNPU, KIF20A, L3MBTL1, LIG3, LMNB1, LRPPRC, MAPK12, MCM6, MEF2C, MIS18BP1, MLXIPL, MYC, MYO16, MYO6, NFE2, NFYB, NR2C1, NR5A2, NTHL1, NUDT16, ORC2, PDS5B, PGR, POLB, POLD2, POLH, POLR1B, POLR2E, POLR2I, POLR3C, POLR3E, POLR3G, POLR3GL, POT1, PPID, PPM1A, PSIP1, PSMC2, PSPC1, RAD51B, RAD51C, RBMX, RNGTT, RPS6KA3, RPS6KB2, RTCA, RUVBL1, SFPQ, SNAPC5, SNRPD1, SRSF10, SRSF7, STAT3, STAT5A, STK24, SYNCRIP, TAF4B, TBL1X, TCEA1, TEAD4, THRB, UBE2E1, VHL, VRK1, WDHD1, WDR12, WDR18, WDR4, WT1
**c1**	**pigment biosynthetic process(GO:0046148)**	1.09E-03	ADA, APRT, CITED1, CPOX, GART, HMBS, MCM6, PAICS, PPOX, SLC25A39, SLC45A2, TSPO
**c1**	electron carrier activity(GO:0009055)	1.38E-03	ADH5, AKR7A2, ALDH2, ALDH4A1, CYBB, CYC1, CYP2F1, CYP2R1, CYP4V2, DHDH, DHRS3, DNAJC10, ETFDH, GSTO2, ME2, NDUFAF2, NDUFS6, NQO2, PHGDH, PHYH, PTGES2, QDPR, SDHB, TMX3, TSTA3, TXNRD3
**c1**	**identical protein binding(GO:0042802)**	1.44E-03	AADAT, ABCB10, ACADM, ACAT1, ACCS, ADH5, AKT1, ALDH4A1, ALDH5A1, ANXA1, APIP, APOBEC3G, ATIC, ATP2A1, BAIAP2, BCAT1, BST2, BTK, C10orf88, CARD8, CARD9, CARS, CASP8, CAT, CAV2, CBS, CBY1, CCDC91, CITED1, CLCN3, CLDN7, CMYA5, CPOX, CR2, CTSC, DYNLL1, ENO3, EPHX2, FADD, FBXO4, FHIT, FOXP1, GSTA4, GYPA, HAND2, HES1, HHEX, HLCS, HOMER1, IDH1, IMPA2, IRAK1, IZUMO1, KIT, L3MBTL1, LCN2, LHPP, LZTFL1, MAP3K13, MAP3K5, MCM6, MECOM, MSH2, MTHFD1L, MTPAP, NME1, NR2C1, NTRK1, NUDT16, OXCT1, PAFAH1B1,

			PAICS, PCM1, PCSK9, PEX7, PFKL, PON2, PRC1, PRKX, PRMT2, PSPH, PYCR1, PYGL, QDPR, QPRT, RAG1, RPS19, RUNX1, RUVBL2, SETMAR, SH3GL3, SOD2, TIMM10, TMEM173, TMTC2, TMTC4, TPD52L1, TTC32, VEGFA, VRK1
**c1**	regulation of Golgi to plasma membrane protein transport(GO:0042996)	1.87E-03	ACSL3, CNST, LYPLAL1, PKDCC
**c1**	**cilium assembly(GO:0042384)**	1.97E-03	AHI1, B9D1, BBIP1, CBY1, CC2D2A, DYNC2LI1, INTU, NEK1, PCM1, TMEM107, TMEM237, TMEM67, VANGL2, WDR35
**c1**	centrosome(GO:0005813)	1.98E-03	AHI1, ARL2, B9D1, BRSK1, CASP8, CCDC15, CCNB1, CCT5, CEP70, CETN3, CNTRL, DCTN2, DIS3L, DLGAP5, DYNLL1, FEZ1, HEPACAM2, IFT80, IFT81, KIAA0368, KIF13A, KIF2A, LRRC45, LRRCC1, MARCKS, MZT2B, NEK1, NME1, NME7, PAFAH1B1, PCM1, PIBF1, PPP1R42, PPP2R3C, PRKAR2B, RABL6, RGCC, RMDN1, SERPINB6, TCEA2, TMEM67, TNFAIP3, TUBGCP5, USP33, WDR35
**c1**	**RNA binding(GO:0003723)**	2.63E-03	ALKBH8, APOBEC3G, APTX, C1QBP, CARS, CELF4, CIRBP, CNOT6, CPSF2, CSTF2, CSTF3, DCPS, DDX10, DDX43, DDX52, DDX60L, DHFRL1, DIMT1, DIS3L, DKC1, DLX2, DZIP3, EEF1A2, EEF2K, EPRS, EXOSC2, EXOSC5, EXOSC8, FIP1L1, FUBP1, FUS, GSPT1, GTF2H3, HBS1L, HEXIM2, HNRNPD, HNRNPK, HNRNPU, ICT1, IGF2BP3, LARP1B, LARP4, LIN28B, LONP1, LRPPRC, LSM5, MAGOHB, MARS, MAZ, MEF2C, MEX3A, MPHOSPH6, MRM1, MRPL21, MRPL23, MTIF2, MTPAP, NSUN6, NUDT16, NUDT7, NXF5, PABPC1L, PNPT1, PSPC1, PUS7, QKI, RAD51AP1, RBM12, RBM41, RBMX, RDM1, RPL26, RPL34, RPL41, RPUSD2, RTCA, SEPSECS, SFPQ, SNRPD1, SRSF10, SRSF7, STRBP, SYNCRIP, TAF15, TARBP1, TCEA1, TCEA2, TDRD7,

			THOC2, THOC7, TIA1, TRA2B, TRDMT1, TRMT1L, UNC50, WDHD1, WT1, XRN1, YARS, ZC3HAV1, ZNF239
**c1**	oxidative phosphorylation(GO:0006119)	2.93E-03	ATP5D, CHCHD10, GBAS, MSH2, SURF1
**c1**	**motile primary cilium(GO:0031512)**	3.17E-03	DYNC2LI1, IFT46, IFT88, PAFAH1B1
**c1**	myeloid progenitor cell differentiation(GO:0002318)	4.00E-03	KIT, MLF1, RUNX1
**c1**	**proton-transporting ATP synthase complex, coupling factor F(o)(GO:0045263)**	4.12E-03	ATP5G3, ATP5H, ATP5I, ATP5J2, ATP5L
**c1**	regulation of megakaryocyte differentiation(GO:0045652)	4.34E-03	HIST1H4C, HIST1H4H, HIST1H4I, L3MBTL1, MEF2C, PF4, SCIN
**c1**	**intraflagellar transport(GO:0042073)**	4.96E-03	IFT43, IFT46, NME7, TTC21B
**c2**	nucleus(GO:0005634)	7.15E-22	ACIN1, ACRC, ACSL6, ACSS2, ADAM10, ADI1, ADNP2, ADRM1, AEN, AFF4, AFTPH, AGO2, AHNAK, AIM2, AKAP17A, ALAS1, ALG2, ALOX5AP, AMIGO2, ANKHD1, ANKS1A, ANLN, ANO7, AP5Z1, APC, APLP2, APPBP2, APPL1, ARF3, ARHGAP28, ARID3B, ARL5B, ARL6, ARL8B, ARNTL2, ASCC3, ATF6, ATXN1L, BAIAP2, BATF, BATF2, BATF3, BBS9, BCL6, BCL9L, BCLAF1, BDH1, BHLHB9, BIRC7, BMP2K, BRAF, BRCC3, BRMS1, BRMS1L, BRPF1, BRWD1, C1orf174, C9orf89, CALCOCO1, CAMKK2, CAPN2, CARD8, CASC5, CASP2, CASP7, CASP8, CBFA2T2, CBX6, CCNL1, CCRN4L, CDC5L, CDC6, CDK20, CDKN1A, CDT1, CEBPE, CELF1, CELF4, CENPBD1, CEP152, CEP97, CHAMP1, CHD2, CHD4, CHMP7, CIC, CIR1, CIRBP, CLK4, CLN3, CLSTN1,

CNOT6, COPRS, CPNE1, CRBN, CREB3, CREB3L2, CREB3L3, CREB5, CREBBP, CRK, CRTC2, CRY1, CRYM, CSRNP1, CSRNP2, CSRP1, CSTF3, CTSA, CWC22, DAPK3, DAXX, DCLRE1C, DDB1, DEDD2, DGKH, DHCR24, DIDO1, DIEXF, DIP2B, DLX2, DMAP1, DNAJC14, DOT1L, DPF2, DPP3, DSCR3, DSN1, DTX3L, DUSP1, DUSP3, DVL2, DYRK1A, DYRK1B, EDC4, EGR1, EHD2, EHMT2, EID2, EID3, ELF4, EPAS1, ERCC1, ERCC6L2, ERRFI1, ESRP2, ESRRB, ETS1, ETS2, EXOSC2, FAM111A, FAM50A, FAM9B, FAN1, FANCC, FBXL3, FBXO3, FBXO32, FEM1B, FIGNL1, FILIP1L, FIP1L1, FLCN, FLNA, FMR1, FNIP2, FOS, FOSB, FOSL2, FOXA3, FOXJ2, FOXJ3, FOXL2, FOXM1, FOXP1, FOXS1, FSD1, FTO, FUBP1, GADD45B, GATAD2A, GBP2, GCFC2, GEM, GLRX, GNB5, GON4L, GPRC5B, GPS2, GTF2IRD1, GZMB, HBP1, HDAC10, HDAC5, HELQ, HIF1AN, HIST1H2AD, HIST1H2AE, HIST1H2AL, HIST1H2AM, HIST1H4B, HIST1H4E, HIST2H2BE, HMGB3, HMGXB3, HNF4A, HNMT, HOXB6, HPSE, HRAS, HSF2, HSPA1A, HSPA1B, HUS1, ICK, ID2, IER3, IFI16, IGFN1, IMP3, INO80D, IP6K1, IPMK, IRF2BP1, IRF7, ITPKC, JMY, JUN, JUP, KAT6A, KCTD5, KDM3A, KDM4C, KEAP1, KIAA0368, KIAA1429, KIAA2018, KLF2, KLF3, KLF4, KLF5, KLHL41, LCORL, LIMK2, LMNA, LOXL2, LPIN1, LRPPRC, LSG1, LUC7L, MACC1, MAFA, MAGEA11, MAGI3, MAP3K7CL, MAPK7, MAU2, MBD1, MBD6, MDC1, MDM1, MDM2, MED15, MED26, MEF2A, MEF2D, MEIS3, MEX3A, MEX3B, MIS12, MLF1, MNX1, MOB1B, MPO, MSL3, MSRB1, MT1G, MT2A, MTF1, MUM1, MXD1, MYNN, NAA11, NAB1, NAB2, NASP, NAV2, NDNL2, NDRG1, NDRG2, NEDD4L, NEDD9, NEK7, NFAT5, NFATC3, NFKBIA, NFRKB, NFX1, NLK, NOL8, NOP58, NR1D2, NR1I3, NR2C2, NR2F2, NR3C1, NR4A1, NR6A1, NUDT16, NUP98, NXF5, NXN, OAS3, PAF1, PARD6B, PASK, PCBP1, PDCD2, PDGFRB, PDXK, PELI1, PER1, PER2, PGR, PHF1, PHF3,

PHLDA1, PHTF2, PIAS1, PIP4K2A, PKM, PLAG1, PLAUR, PLEKHA1, PNKD, PNRC1, POLH, POLR2A, POLR2L, PPARD, PPHLN1, PPIG, PPL, PPM1A, PPM1D, PPP1R8, PPP3CA, PPP4R2, PPP6R3, PRDM10, PRICKLE1, PRKCI, PRM3, PRMT2, PRPF18, PRPF4, PSMC2, PSPC1, PTK2B, PTMA, PTOV1, PTPN23, PTPN6, QKI, RAB17, RAB21, RAB22A, RAB26, RAB32, RAB33A, RAB33B, RAB38, RAB3D, RAB7A, RABL6, RAD51B, RAD52, RAD54L2, RANBP10, RASD1, RBM19, RBM23, RCBTB1, RCOR3, RELA, RELB, RFX1, RFX3, RLF, RNASEH2C, RNF40, RPF2, RPP25, RRAGB, RRAGC, RSF1, RSPH1, RSRC1, RUNX1, RUNX2, RXRA, S100A13, S100A16, S100A6, SAMD4B, SART1, SCAF4, SCAF8, SELM, SERTAD2, SESN3, SETD1A, SETD1B, SETD7, SETMAR, SETX, SF3B3, SFR1, SGK1, SH3BGRL3, SHOC2, SIN3A, SIPA1, SIX1, SKI, SKIL, SKIV2L, SLC2A1, SLC35A2, SLC7A6OS, SLFN11, SLU7, SMAD6, SMCR8, SMG1, SMNDC1, SNAP29, SNIP1, SOCS1, SOCS7, SOD3, SOHLH1, SP110, SP4, SP6, SPATA2, SRFBP1, SRRM1, SRRM2, SRSF10, SRSF4, SSX5, STAT1, STAT3, STK24, STXBP3, SUPT5H, SWAP70, SYNCRIP, SYTL4, TAF13, TAF4, TBC1D2, TBL1Y, TBX19, TCEAL7, TCEANC, TCEB3, TEAD4, TERF2, TESK2, TEX19, TFE3, TGFB1, THRA, THRAP3, THRB, TIA1, TIGD7, TJAP1, TNFAIP1, TNFRSF1A, TNIP1, TOPORS, TP53BP1, TP53INP1, TP53INP2, TP53RK, TP73, TRA2B, TRAK1, TRIB1, TRIM11, TRIM21, TRIM23, TRIM24, TRIM25, TRIM27, TRIM5, TRIM68, TRMT6, TRO, TROVE2, TXNRD1, U2AF2, UBD, UBE2I, UBE2Z, UBN1, UBOX5, UBQLN2, UBTD2, UBTF, UBXN1, UHRF2, UIMC1, UNKL, USP11, UTRN, VDR, VEZF1, VPS36, VPS37C, VWA5A, WBP4, WDR33, WIPI2, WRNIP1, WTAP, WTIP, YBEY, ZBTB1, ZBTB24, ZBTB26, ZBTB39, ZBTB41, ZBTB43, ZBTB45, ZBTB49, ZBTB5, ZBTB9, ZC3HAV1, ZFAND2A, ZFP1, ZFP36, ZFP37, ZFPM1, ZFX, ZGPAT, ZIC5, ZMIZ2, ZMYM3, ZMYND11,

			ZNF107, ZNF112, ZNF124, ZNF134, ZNF136, ZNF140, ZNF165, ZNF180, ZNF189, ZNF2, ZNF200, ZNF207, ZNF211, ZNF212, ZNF213, ZNF222, ZNF224, ZNF226, ZNF229, ZNF235, ZNF253, ZNF26, ZNF263, ZNF264, ZNF268, ZNF280A, ZNF282, ZNF292, ZNF296, ZNF304, ZNF317, ZNF318, ZNF350, ZNF37A, ZNF394, ZNF425, ZNF432, ZNF44, ZNF446, ZNF449, ZNF468, ZNF480, ZNF490, ZNF502, ZNF528, ZNF530, ZNF541, ZNF577, ZNF579, ZNF594, ZNF600, ZNF616, ZNF626, ZNF628, ZNF629, ZNF652, ZNF658, ZNF678, ZNF684, ZNF69, ZNF701, ZNF721, ZNF75A, ZNF771, ZNF776, ZNF789, ZNF79, ZNF805, ZNF808, ZNF837, ZNF841, ZNF844, ZNF846, ZNF93, ZSCAN12, ZSWIM7, ZXDC
**c2**	regulation of nucleobase, nucleoside, nucleotide and nucleic acid metabolic process(GO:0019219)	4.98E-15	ABLIM1, ACVR1, ADI1, ADM, ADNP2, AGAP1, AGO2, AIM2, AKAP12, AKAP17A, ALS2, AMH, ARHGEF19, ARID3B, ARNTL2, ASCC2, ASCC3, ASPH, ASXL1, ATF6, ATP8B1, ATXN1L, BATF, BATF2, BATF3, BCL6, BCL9L, BCLAF1, BMPR2, BRCC3, BRMS1, BRMS1L, BRPF1, BRWD1, CALCOCO1, CALCRL, CALM1, CAMKK2, CBFA2T2, CBX6, CCL5, CCNK, CCNL1, CCRN4L, CDC42EP2, CDC5L, CDC6, CDKN1A, CDT1, CEBPE, CELF1, CELF4, CGA, CHD2, CHD4, CIC, CIR1, CIRBP, CLCF1, CLK4, CNOT6, COPRS, CREB3, CREB3L2, CREB3L3, CREB5, CREBBP, CRK, CRTC2, CRY1, CRYM, CSRNP1, CSRNP2, CWC22, CYLD, CYR61, DAPK3, DAXX, DDX58, DEDD2, DLX2, DMAP1, DPF2, DRD5, DVL2, DYRK1B, ECM1, EFNA1, EGR1, EHMT2, EID2, EID3, ELF4, ELMOD2, EPAS1, EPC2, ERCC1, ERRFI1, ESCO2, ESRP2, ESRRB, ETS1, ETS2, F2RL1, FGD6, FLCN, FLNA, FNIP1, FNIP2, FOS, FOSB, FOSL2, FOXA3, FOXJ2, FOXJ3, FOXL2, FOXM1, FOXP1, FOXP4, FOXS1, FZD7, GABBR1, GATAD2A, GCFC2, GON4L, GPS2, GTF2IRD1, GUCA1B, HBP1, HDAC10, HDAC5, HIPK1, HNF4A, HOXB6, HRAS, HRH2, HSF2, HTR1D,

	HUS1, ID2, IER3, IFI16, IL11, IL1A, IL5, INO80D, IRF2BP1, IRF7, JMJD1C, JMY, JUN, JUP, KAT6A, KDM3A, KDM4C, KEAP1, KLF2, KLF4, KLF5, LCORL, LGR4, LOXL2, LPIN1, LRPPRC, LZTR1, MACC1, MAFA, MAP3K10, MAP3K13, MAPK7, MBD1, MC2R, MC3R, MDM2, MED10, MED15, MED26, MED29, MEF2A, MEF2D, MEIS3, MEPCE, MET, MNX1, MRAP2, MTF1, NAB1, NAB2, NDNL2, NEDD4L, NFAT5, NFATC3, NFKBIA, NFRKB, NFX1, NGEF, NLK, NLRP5, NR1D2, NR1I3, NR2C2, NR2F2, NR3C1, NR4A1, NR6A1, NRBF2, NUDT16, PAF1, PASK, PDGFRB, PDPK1, PELI1, PER1, PER2, PGR, PHF19, PHTF2, PIAS1, PIK3R1, PKIG, PLAC8, PLAG1, PLAUR, PNRC1, POLH, POLR2A, POLR2L, POT1, PPAP2B, PPARD, PPM1A, PPP1R8, PPP3CA, PPP4R2, PRDM10, PRICKLE1, PRKCI, PRMT2, PRRX2, PSPC1, PTK2B, PTOV1, RAPGEF2, RASA4, RASD1, RASGRP3, RC3H1, RCBTB1, RCOR3, RELA, RELB, RFX1, RFX3, RICTOR, RIPK1, RLF, RPRD1A, RPS6KA3, RSF1, RUNX1, RUNX2, RXRA, S1PR1, S1PR3, SAMD4B, SAP130, SAP30L, SCGB1A1, SERPINE1, SERTAD1, SERTAD2, SETD1A, SETD1B, SETD7, SGK1, SIN3A, SIPA1, SIRT5, SIX1, SKI, SKIL, SLC9A3R1, SMAD6, SNIP1, SOHLH1, SOS1, SP110, SP4, SP6, SQSTM1, SRFBP1, SRGAP2, SRRT, SRSF10, SRSF4, SSX5, STAT1, STAT3, STK16, SUPT5H, SYDE2, SYNCRIP, TAB3, TADA2B, TAF4, TAF7L, TBC1D10B, TBC1D10C, TBC1D15, TBC1D17, TBC1D19, TBC1D2, TBCK, TBL1Y, TBX19, TCEAL7, TCEANC, TCEB2, TCEB3, TEAD4, TERF2, TFE3, TGFB1, THRA, THRAP3, THRB, TIA1, TIRAP, TNFAIP1, TNFRSF1A, TNIP1, TOB1, TOPORS, TP53BP1, TP53INP1, TP53INP2, TP73, TPM1, TRA2B, TRAK1, TRIB1, TRIM11, TRIM21, TRIM24, TRIM25, TRIM27, TRIM29, TRIM5, TSC1, U2AF2, UBE2I, UBN1, UBTF, UIMC1, USP47, USP9X, VDR, VEZF1, VPS36, WRNIP1, WTIP, ZBTB1, ZBTB24, ZBTB26, ZBTB39, ZBTB41, ZBTB43, ZBTB45, ZBTB49, ZBTB5,

			ZBTB9, ZC3HAV1, ZFP1, ZFP36, ZFP37, ZFPM1, ZFX, ZGPAT, ZMIZ2, ZMYND11, ZNF107, ZNF112, ZNF124, ZNF134, ZNF136, ZNF140, ZNF165, ZNF180, ZNF189, ZNF2, ZNF200, ZNF207, ZNF211, ZNF212, ZNF213, ZNF222, ZNF224, ZNF226, ZNF229, ZNF235, ZNF253, ZNF26, ZNF263, ZNF264, ZNF268, ZNF280A, ZNF282, ZNF292, ZNF296, ZNF304, ZNF317, ZNF318, ZNF350, ZNF37A, ZNF394, ZNF425, ZNF432, ZNF44, ZNF446, ZNF449, ZNF468, ZNF480, ZNF490, ZNF502, ZNF528, ZNF530, ZNF541, ZNF577, ZNF579, ZNF594, ZNF600, ZNF616, ZNF626, ZNF628, ZNF629, ZNF652, ZNF658, ZNF678, ZNF684, ZNF69, ZNF701, ZNF721, ZNF75A, ZNF771, ZNF776, ZNF789, ZNF79, ZNF805, ZNF808, ZNF837, ZNF841, ZNF844, ZNF846, ZNF93, ZSCAN12, ZXDC
**c2**	cellular macromolecule metabolic process(GO:0044260)	1.04E-14	AARS2, ACVR1, ADAM10, ADAT2, ADAT3, ADM, ADNP2, ADRM1, AFF4, AGA, AGO2, AKAP17A, ALAS2, ALG2, ALKBH1, ALS2, AMHR2, ANAPC16, AP5Z1, APH1B, APOL2, ARFGEF2, ARID3B, ARSA, ART5, ASB6, ASCC2, ASCC3, ASPH, ASPHD1, ASXL1, ATF6, ATP6V0D1, ATXN1L, AVPI1, AXL, B3GALT6, B3GNT6, B4GALNT1, BAG3, BATF, BATF2, BATF3, BCL6, BCL9L, BCLAF1, BIRC7, BMP2K, BMPR2, BRAF, BRCC3, BRMS1, BRMS1L, BRPF1, BRPF3, BRWD1, C5, CACUL1, CALCOCO1, CALCRL, CALM1, CAMKK2, CBLL1, CBX6, CCL2, CCL5, CCNK, CCNL1, CCRN4L, CDC5L, CDC6, CDK19, CDK20, CDKL3, CDT1, CEBPE, CELF1, CELF4, CETN2, CGA, CHD4, CHERP, CHM, CIC, CIR1, CIRBP, CLK4, CLN3, CNNM3, CNOT6, CNP, COPRS, CRBN, CREB3, CREB3L2, CREB3L3, CREB5, CREBBP, CRK, CRTC2, CRY1, CSF1R, CSRNP1, CSRNP2, CSTF3, CWC22, CYLD, DAPK3, DAPP1, DAXX, DCLRE1C, DDB1, DEDD2, DERL3, DGUOK, DHCR24, DIDO1, DMAP1, DNAJB2, DNAJB9, DNASE1L1, DOT1L, DPF2, DPM3, DTX3L, DTX4, DUSP1, DUSP13, DUSP3,

DUSP5, DVL2, DYRK1A, DYRK1B, EDC4, EDEM1, EFNA1, EGR1, EHMT2, EID2, EID3, EIF2AK3, EIF2S2, EIF5, ELF4, EPAS1, EPC2, EPHB6, ERCC1, ERCC6L2, ESCO2, ESRP2, ESRRB, ETF1, ETS1, EXOC2, EXOSC2, FAN1, FANCB, FANCC, FANCD2, FBXL3, FBXO10, FBXO3, FBXO32, FBXO33, FBXO4, FEM1B, FIGNL1, FIP1L1, FKBP1A, FLNA, FNIP2, FOS, FOSL2, FOXJ3, FOXL2, FOXM1, FOXP1, FRS2, FTO, FUBP1, FUT3, GAD1, GADD45B, GALNT18, GATAD2A, GBE1, GCFC2, GCNT3, GIN1, GNMT, GOSR2, GPATCH1, GPC1, GSPT1, GXYLT1, HBP1, HBS1L, HDAC10, HDAC5, HIF1AN, HIPK1, HIST1H4B, HIST1H4E, HMGB3, HNF4A, HOXB6, HPSE, HRAS, HSF2, HSPA1A, HSPA1B, HSPA9, HUS1, ICK, IFI16, IGFBP6, IMP3, INO80D, INTS5, IRF2BP1, IRF7, ISG20, JKAMP, JMJD1C, JMY, JUN, KAT6A, KATNAL1, KCTD11, KDM3A, KDM4C, KEAP1, KIAA0368, KIAA1429, KLF2, KLF3, KLF4, KLF5, KLHL15, KLHL21, KLHL41, KSR1, LATS1, LCMT2, LCORL, LDLR, LIMK2, LMNA, LMTK2, LOXL2, LPIN1, LRP4, LRPPRC, MACC1, MAFA, MANBA, MAP3K10, MAP3K12, MAP3K13, MAP6D1, MAPK7, MBD1, MDC1, MDM2, MED10, MED15, MED26, MED29, MEF2A, MEF2D, MEPCE, MET, MEX3B, MGAT4A, MKRN1, MKRN3, MLEC, MLF1, MLKL, MME, MMP1, MMP13, MNX1, MOB1B, MRPL1, MSL2, MSL3, MSRB1, MTF1, MUM1, MXD1, MYNN, NAB1, NAB2, NASP, NDNL2, NEDD4L, NEK7, NFAT5, NFATC3, NFRKB, NFX1, NKTR, NLK, NOL8, NOP58, NR1D2, NR1I3, NR2C2, NR3C1, NR4A1, NR6A1, NRBF2, NRBP1, NUDT16, NUP214, NUP98, OXSR1, P4HA2, PAF1, PAK4, PAPPA, PASK, PATL1, PCBP1, PCF11, PCSK6, PDGFRB, PDK3, PDPK1, PELI1, PELI2, PER1, PER2, PGR, PHF1, PHF19, PHF3, PHLDA1, PHTF2, PIAS1, PIGC, PIGQ, PIGU, PIGV, PIK3R1, PIK3R4, PINK1, PJA2, PLAG1, PLAT, PLAUR, PMM2, PNRC1, POLH, POLR2A, POLR2L, POMT2, POT1, PPARD,

PPIG, PPM1A, PPM1B, PPM1D, PPP1R8, PPP3CA, PPP4R2, PRDM10, PRKCI, PRMT2, PRPF18, PRPF4, PSMC2, PSPC1, PTGER3, PTK2B, PTMA, PTOV1, PTPN23, PTPN6, PTPRF, PTPRJ, PXN, QKI, RAB3D, RAB7A, RAD51B, RAD52, RBM23, RC3H1, RCBTB1, RCOR3, RELB, RFX3, RHBDD1, RHO, RIPK1, RLF, RNASE4, RNASEH2C, RNF115, RNF185, RNF19B, RNF213, RNF40, RNF43, RPL17, RPP25, RPRD1A, RPS6KA3, RRAGC, RSF1, RSL1D1, RSRC1, RUNX1, RUNX2, RXRA, SAMD4B, SAP130, SAP30L, SART1, SCAF8, SCGB1A1, SCYL3, SEC24A, SEC24B, SERPINE1, SERTAD1, SERTAD2, SETD1A, SETD1B, SETD7, SETMAR, SETX, SF3B3, SF3B4, SFR1, SGK1, SIN3A, SIRT3, SIRT5, SKI, SKIL, SLK, SLU7, SLX4, SMAD6, SMC6, SMG1, SMNDC1, SNIP1, SOCS1, SOCS4, SOCS5, SOCS6, SOCS7, SOHLH1, SP110, SP4, SP6, SPRR3, SPSB3, SQSTM1, SREK1IP1, SRFBP1, SRP68, SRRM1, SRRM2, SRRT, SRSF10, SRSF4, SSH1, SSH2, SSX5, ST8SIA4, STAT1, STAT3, STK16, STK24, STX1A, SUPT5H, SWAP70, SYNCRIP, TAB3, TADA2B, TAF13, TAF4, TAF7L, TAOK1, TATDN2, TBCK, TBL1Y, TBX19, TCEAL7, TCEANC, TCEB2, TCEB3, TEAD4, TERF2, TESK1, TESK2, TEX14, TFE3, TGFB1, THRA, THRAP3, THRB, TIMM10B, TIRAP, TMEM67, TNFAIP1, TNIP1, TOPORS, TP53BP1, TP53INP1, TP53INP2, TP53RK, TP73, TRA2B, TRAK1, TRIB1, TRIM21, TRIM23, TRIM24, TRIM27, TRIM29, TRIM38, TRIM5, TRIM56, TRIM68, TRMT6, TROVE2, TSEN34, TTLL7, TUBA1A, TUBB2A, TUBB3, TUT1, TXNRD1, U2AF2, UBAP1, UBD, UBE2I, UBE2J1, UBOX5, UBQLN2, UBTF, UBXN1, UHRF2, UIMC1, UNKL, USP11, USP12, USP31, USP32, USP36, USP47, USP53, USP9X, VDR, VEZF1, VIMP, VPS36, WBP4, WDR33, WIBG, WRNIP1, WTAP, WTIP, XRN1, YBEY, ZBTB1, ZBTB24, ZBTB26, ZBTB39, ZBTB41, ZBTB43, ZBTB45, ZBTB49, ZBTB5, ZBTB9, ZFP1, ZFP36, ZFP37, ZFX, ZFYVE26, ZGPAT, ZMIZ2, ZMYND11, ZNF107,

			ZNF112, ZNF124, ZNF134, ZNF136, ZNF140, ZNF165, ZNF180, ZNF189, ZNF2, ZNF200, ZNF211, ZNF212, ZNF213, ZNF222, ZNF224, ZNF226, ZNF229, ZNF235, ZNF253, ZNF26, ZNF263, ZNF264, ZNF268, ZNF280A, ZNF282, ZNF296, ZNF304, ZNF317, ZNF318, ZNF350, ZNF37A, ZNF394, ZNF425, ZNF432, ZNF44, ZNF446, ZNF449, ZNF468, ZNF480, ZNF490, ZNF502, ZNF528, ZNF530, ZNF541, ZNF577, ZNF579, ZNF594, ZNF600, ZNF616, ZNF626, ZNF628, ZNF629, ZNF652, ZNF658, ZNF678, ZNF684, ZNF69, ZNF701, ZNF721, ZNF75A, ZNF771, ZNF776, ZNF789, ZNF79, ZNF805, ZNF808, ZNF837, ZNF841, ZNF844, ZNF846, ZNF93, ZSCAN12, ZSWIM7, ZXDC
**c2**	protein binding(GO:0005515)	1.91E-14	ABCB1, ABLIM1, ABTB2, ACIN1, ACSL6, ACVR1, ADAM10, ADAMTSL4, ADI1, ADM, ADNP2, ADRM1, AFF4, AFTPH, AGA, AGAP1, AGO2, AHNAK, AIM1L, AIM2, AKAP12, AKAP17A, AKR1B10, ALAS2, ALDOC, ALG2, ALKBH1, ALOX5AP, ALS2, AMBRA1, AMH, AMHR2, AMIGO2, ANK1, ANKFY1, ANKHD1, ANKHD1-EIF4EBP3, ANKLE2, ANKMY1, ANKRD10, ANKRD20A3, ANKRD30BL, ANKRD62, ANKS1A, ANLN, ANXA2, ANXA5, ANXA9, AP1G1, AP1G2, AP5Z1, APC, APH1B, APLP2, APOD, APOL2, APPBP2, APPL1, ARC, ARF3, ARFGEF2, ARFIP2, ARHGAP12, ARHGAP23, ARHGAP25, ARHGEF18, ARHGEF19, ARL5B, ARL6, ARL8B, ARMC8, ARMC9, ARMCX1, ARNTL2, ARPC2, ASB6, ASCC2, ASCC3, ASIP, ASPH, ASXL1, ATF6, ATG14, ATG16L1, ATG2A, ATP11A, ATP2A2, ATP2B1, ATP6V0D1, ATP6V0D2, ATP6V1C1, ATP6V1D, ATP6V1E1, ATP6V1H, ATP8B1, ATRAID, ATRN, ATXN1L, AVIL, AXL, AZI2, BAG3, BAIAP2, BAIAP3, BATF, BATF2, BBS9, BCAR1, BCAR3, BCAT1, BCL6, BCL7B, BCL9L, BCLAF1, BFSP1, BIN2, BIN3, BIRC7, BLCAP, BMF, BMPR2, BRAF, BRAP, BRCC3, BRMS1, BRPF1, BRPF3, BRWD1, BTBD7, C17orf59, C2orf72, C3, C3AR1, C5, C9orf89, CACNB3,

CACUL1, CALCOCO1, CALCRL, CALM1, CALY, CAMK2N1, CAMKK2, CAPN2, CARD6, CARD8, CASC5, CASP2, CASP7, CASP8, CASS4, CATSPER1, CBFA2T2, CBLL1, CBX6, CC2D2A, CCK, CCL2, CCL5, CCNK, CCNL1, CCR10, CCRN4L, CCSER2, CD244, CD274, CD276, CD2AP, CD44, CD59, CD82, CD9, CDC42EP2, CDC42EP3, CDC42EP4, CDC5L, CDC6, CDK20, CDKN1A, CDT1, CEBPE, CELF1, CEP152, CEP170B, CEP97, CETN2, CGA, CHAMP1, CHD4, CHERP, CHKA, CIC, CIR1, CIRBP, CLCA1, CLCF1, CLCN6, CLDN15, CLIP2, CLIP4, CLN3, CMTM7, CNNM3, CNNM4, CNOT6, CNPY2, CNPY3, CNPY4, CNST, COL13A1, COL16A1, COL1A2, COL5A1, COL7A1, COPB1, COPRS, CPNE1, CPNE5, CRBN, CREB3, CREB3L3, CREB5, CREBBP, CRIM1, CRIP2, CRK, CROT, CRY1, CRYM, CSF1R, CSF2RB, CSF3R, CSRNP2, CSRP1, CTSA, CWC22, CYHR1, CYLD, CYP1A1, CYP4F2, CYR61, DAAM1, DACT3, DAPK3, DAPP1, DAXX, DCUN1D2, DDB1, DDX58, DEDD2, DERL3, DFNB31, DGKH, DHCR24, DHH, DIAPH2, DIDO1, DIP2B, DLG3, DLK1, DLX2, DMAP1, DNAJB2, DNAJB9, DNAL1, DNMBP, DOCK9, DOT1L, DPF2, DPM3, DPYSL2, DSN1, DTX3L, DTX4, DUSP1, DVL2, DYRK1A, DYRK1B, ECM1, EDC4, EDEM1, EFHD2, EFNA1, EFNA4, EFNB2, EGFL6, EGR1, EHD2, EHMT2, EID2, EID3, EIF2AK3, EIF2S2, EIF5, ELF4, EML6, ENSA, EPAS1, EPB41L1, EPHB6, ERCC1, ERRFI1, ESAM, ESRRB, ETF1, ETS1, ETS2, EXOC2, EXOSC2, F2RL1, F2RL2, F3, FAF2, FAM111A, FAM160A2, FAM43A, FAM65C, FAM89B, FAN1, FANCC, FANCD2, FBLIM1, FBXL20, FBXL3, FBXO10, FBXO28, FBXO3, FBXO32, FBXO33, FBXO4, FCRLA, FCRLB, FEM1A, FEM1B, FGD6, FGFBP3, FIP1L1, FKBP1A, FLCN, FLNA, FLVCR1, FMNL3, FMR1, FNBP1L, FNIP1, FNIP2, FOS, FOSB, FOXA3, FOXJ2, FOXJ3, FOXL2, FOXM1, FOXP1, FOXP4, FOXS1, FRS2, FSD1, FSD1L, FUBP1, FXR2, FZD7, GABBR1, GAD1, GADD45B, GAS2L1,

GATAD2A, GBA, GEM, GIGYF1, GIPC3, GLMN, GLRX, GLS, GNB5, GNMT, GNS, GOLGA4, GOSR1, GPANK1, GPC1, GPR158, GPRASP1, GPS2, GRASP, GRB10, GRN, GSN, GSPT1, GZMB, HBEGF, HBP1, HCST, HDAC10, HDAC5, HGS, HIF1AN, HIPK1, HIST1H2AD, HIST1H2AE, HIST1H2AL, HIST1H2AM, HIST1H4B, HIST1H4E, HIST2H2BE, HLA-E, HNF4A, HPS1, HPS6, HPSE, HRAS, HSF2, HSPA1A, HSPA1B, HSPA9, HUS1, HYAL1, ICK, ID2, IDS, IDUA, IER3, IFI16, IFI6, IFIT3, IFNGR1, IGFBP6, IGFN1, IGSF11, IGSF3, IKBIP, IL11, IL1A, IL1R1, IL2RB, IL5, IL6R, IMP3, INPP4A, INPP5J, INTS5, IP6K1, IPO9, IQCE, IQCF1, IQCG, IRF7, IRS2, IST1, ITFG2, ITGA4, ITGA7, ITGB2, ITPKC, JKAMP, JMJD1C, JMY, JUN, JUP, KAT6A, KBTBD4, KCNA5, KCNE2, KCNJ11, KCNK3, KCTD17, KCTD5, KCTD6, KCTD7, KDM3A, KDM4C, KEAP1, KEL, KIAA0226, KIAA1524, KIAA2018, KIF13A, KIF3A, KIF3B, KLC3, KLF2, KLF4, KLF5, KLHL15, KLHL21, KLHL24, KLHL25, KLHL28, KLHL41, KLHL5, KLHL8, KRT15, KRT17, KRT19, KRT8, KSR1, LAMB3, LAMP1, LATS1, LCMT2, LDLR, LEPR, LEPROT, LGR4, LIFR, LIMK2, LIPG, LLGL2, LMNA, LMTK2, LNX2, LONRF3, LOXL2, LPIN1, LRG1, LRIG2, LRP10, LRP11, LRP4, LRPPRC, LRRC58, LRRC8A, LRRC8C, LSR, LTBP3, LTBR, LUC7L, LY96, LZIC, LZTR1, MACC1, MAFA, MAGEA11, MAGI3, MAOB, MAP1A, MAP3K10, MAP3K12, MAP3K13, MAP3K7CL, MAP6D1, MAPK7, MARCKSL1, MAU2, MBD1, MC2R, MC3R, MDC1, MDM2, MED15, MED26, MED29, MEF2A, MEF2BNB, MEF2D, MET, MEX3A, MEX3B, MIA3, MICA, MICAL3, MICB, MIS12, MKRN1, MKRN3, MLF1, MLKL, MME, MOB1B, MPDZ, MRAP2, MSANTD3, MSL2, MSL3, MT1G, MT2A, MTF1, MUM1, MXD1, MYNN, MYO7A, N4BP2L2, NAB1, NAB2, NAGK, NASP, NAV2, NBR1, NCF2, NDNL2, NDRG1, NDRG2, NEDD4L, NEDD9, NES, NEU1, NFAT5, NFATC3, NFKBIA, NFRKB, NFX1, NGEF, NGFR,

NHLRC2, NLK, NLRP5, NOL8, NOP58, NPC2, NPTN, NR1D2, NR1I3, NR2C2, NR2F2, NR3C1, NR4A1, NR6A1, NRBP1, NRCAM, NUDT16, NUMBL, NUP214, NUP98, NXF5, NXN, OAS3, OPTN, OSBPL7, OSGIN1, OXSR1, P4HA2, PAF1, PAK4, PALM3, PAPPA, PARD6B, PARD6G, PASK, PATL1, PCBP1, PCLO, PCSK6, PCYT1A, PDGFRB, PDK3, PDLIM4, PDPK1, PDXK, PEA15, PELI1, PELI2, PER1, PER2, PEX16, PGF, PGR, PHACTR2, PHF1, PHF19, PHF23, PHF3, PHLDA1, PI4K2A, PI4KA, PIAS1, PIK3R1, PIK3R4, PINK1, PJA2, PKM, PLA2G16, PLAA, PLAC8, PLAT, PLAUR, PLD1, PLEKHA1, PLEKHF2, PLEKHM1, PNRC1, POLR2A, POT1, PPAP2B, PPFIA1, PPL, PPM1A, PPM1B, PPM1D, PPP1R12B, PPP1R8, PPP3CA, PPP3CC, PPP4R1L, PPP4R2, PPP6R3, PPY, PRICKLE1, PRKCI, PRMT2, PROCR, PRPF4, PRSS3, PSMC2, PSPC1, PTGER3, PTK2B, PTPN23, PTPN6, PTPRF, PTPRJ, PXN, QKI, RAB17, RAB21, RAB22A, RAB26, RAB27B, RAB33A, RAB33B, RAB38, RAB3D, RAB7A, RABL6, RAD51B, RAD52, RAD54L2, RALB, RALGPS2, RANBP10, RAPGEF2, RAPH1, RASA4, RASD1, RASGRP3, RASSF3, RC3H1, RC3H2, RCAN3, RCBTB1, RCBTB2, RCOR3, REEP2, RELA, RELB, RFPL4A, RFX1, RFX3, RHBDD1, RHEB, RHO, RHOBTB1, RHOBTB2, RICTOR, RILP, RIN1, RIPK1, RLF, RMND5A, RNASE1, RND1, RNF114, RNF115, RNF166, RNF185, RNF19B, RNF213, RNF38, RNF40, RNF43, ROPN1L, RPH3A, RPP25, RPS6KA3, RRAD, RRAGB, RRAGC, RSF1, RSL1D1, RSRC1, RTN4R, RUNX1, RUNX2, RUSC2, RXRA, RYR2, S100A10, S100A13, S100A14, S100A16, S100A6, SAMD4B, SAMD5, SAMD8, SAMD9, SAP130, SART1, SASH1, SCAF8, SCN4A, SCYL3, SDC2, SDC4, SEC23IP, SEC24B, SERPINB8, SERPINE1, SERTAD1, SERTAD2, SETD1A, SETD1B, SETD4, SETD7, SETMAR, SETX, SF3B3, SF3B4, SFR1, SGK1, SH2B2, SH2D1A, SH3BP5, SH3D21, SHB, SHBG, SHCBP1, SHOC2, SIGLEC14,

	SIGLEC5, SIN3A, SIPA1, SIRT3, SIRT5, SIX1, SKI, SKIL, SLC1A3, SLC22A17, SLC25A4, SLC2A1, SLC39A7, SLC4A1, SLC7A8, SLC9A3R1, SLK, SLX4, SMAD6, SMC6, SMCHD1, SMG1, SMNDC1, SNAP29, SNIP1, SNPH, SNX11, SNX30, SNX7, SOCS1, SOCS4, SOCS5, SOCS6, SOCS7, SOD3, SOHLH1, SOS1, SP110, SPATS2, SPG20, SPINK14, SPINK4, SPINT1, SPNS1, SPRR3, SPSB3, SPTAN1, SQSTM1, SRFBP1, SRGAP2, SRRM2, SRRT, SRSF10, SSH1, SSH2, STAT1, STAT3, STIM2, STK16, STK24, STON2, STX1A, STX3, STXBP3, SULT2A1, SUPT5H, SURF4, SWAP70, SYDE2, SYN1, SYNCRIP, SYNJ1, SYNRG, SYT11, SYTL4, TAB3, TAC1, TAC3, TADA2B, TAF13, TAF4, TANC2, TBC1D15, TBC1D2, TBCCD1, TBL1Y, TBX19, TCAP, TCEB2, TCEB3, TDRKH, TEAD4, TECPR2, TERF2, TESK1, TESK2, TEX14, TFE3, TFRC, TGFB1, THRA, THRAP3, THRB, TIA1, TIMP1, TIRAP, TJAP1, TJP2, TMEM132D, TMEM25, TMEM67, TMSB15A, TMSB15B, TNC, TNFAIP1, TNFRSF1A, TNFRSF9, TNFSF9, TNIP1, TOB1, TOLLIP, TOM1, TOPORS, TOR1AIP1, TP53BP1, TP53I3, TP53INP1, TP53INP2, TP53RK, TP73, TPM1, TPRG1L, TRA2B, TRAK1, TRAPPC10, TREML2, TRIB1, TRIM11, TRIM15, TRIM2, TRIM21, TRIM23, TRIM24, TRIM25, TRIM27, TRIM29, TRIM38, TRIM5, TRIM52, TRIM56, TRIM68, TRO, TROAP, TRPC5, TSC1, TSEN34, TTC26, TTC30B, TTYH2, TUBB3, TUFT1, TUT1, TWF1, TXLNA, TXNRD1, U2AF2, UBAP1, UBD, UBE2I, UBE2Q1, UBFD1, UBL7, UBN1, UBOX5, UBQLN2, UBTD2, UBTF, UBXN1, UHRF2, UIMC1, UNKL, USP11, USP12, USP47, USP9X, UTRN, VASN, VDR, VEPH1, VIL1, VIMP, VMP1, VPS18, VPS36, VPS37B, VPS41, VWA5A, WASF1, WASF2, WBP4, WBP5, WDR33, WDR35, WDR45B, WDR54, WDR6, WDR73, WHAMM, WIBG, WIPF2, WIPI2, WRNIP1, WTIP, XRN1, YKT6, ZBTB1, ZBTB24, ZBTB26, ZBTB39, ZBTB41, ZBTB43, ZBTB45, ZBTB49, ZBTB5, ZBTB9, ZC3HAV1, ZCCHC14,

			ZFP36, ZFPM1, ZFYVE1, ZFYVE26, ZFYVE9, ZGPAT, ZMIZ2, ZMYND11, ZNF106, ZNF136, ZNF165, ZNF212, ZNF224, ZNF350, ZNF622, ZNF652, ZSWIM7, ZXDC
**c2**	zinc ion binding(GO:0008270)	3.17E-14	ABLIM1, ADAM10, ADAT2, ADAT3, ADNP2, AGAP1, ANKMY1, ARHGEF18, BCL6, BIRC7, BRAP, BRPF1, BRPF3, CA9, CBFA2T2, CBLL1, CHAMP1, CHD4, CIR1, CREB5, CREBBP, CRIP2, CSRP1, CYHR1, CYLD, DDX58, DHH, DIDO1, DPF2, DPP3, DTX3L, DTX4, EGR1, EHMT2, ESRRB, FBLIM1, GATAD2A, HIF1AN, HNF4A, KAT6A, KCTD7, KDM4C, KLF2, KLF3, KLF4, KLF5, LIMK2, LNX2, LONRF3, MAN2B2, MBD1, MDM2, MEX3A, MEX3B, MICAL3, MKRN1, MKRN3, MME, MMP1, MMP13, MMP17, MSL2, MSRB1, MT1G, MT2A, MTF1, MYNN, NBR1, NFX1, NR1D2, NR1I3, NR2C2, NR2F2, NR3C1, NR4A1, NR6A1, PAPPA, PDCD2, PDLIM4, PDXK, PGLYRP4, PGR, PHF1, PHF19, PHF23, PHF3, PIAS1, PJA2, PLAG1, PLAUR, PNKD, POLR2L, PPARD, PRDM10, PRICKLE1, PRKCI, PTGR1, PXN, RC3H1, RC3H2, RFPL4A, RLF, RNF114, RNF115, RNF166, RNF185, RNF19B, RNF213, RNF38, RNF40, RNF43, RPH3A, RSF1, RTN4IP1, RXRA, S100A13, S100A6, SEC24A, SEC24B, SETMAR, SIRT3, SIRT5, SKI, SLU7, SOD3, SP110, SP4, SP6, SQSTM1, SREK1IP1, SYTL4, TAB3, TADA2B, TCEANC, TES, THRA, THRB, TOPORS, TP53I3, TRIM11, TRIM15, TRIM2, TRIM21, TRIM23, TRIM24, TRIM25, TRIM27, TRIM29, TRIM38, TRIM5, TRIM52, TRIM56, TRIM68, UBOX5, UHRF2, UNK, UNKL, UTRN, VDR, VEZF1, VPS41, WBP4, WTIP, ZBTB1, ZBTB24, ZBTB26, ZBTB39, ZBTB41, ZBTB43, ZBTB45, ZBTB49, ZBTB5, ZBTB9, ZC3HAV1, ZCCHC14, ZCCHC24, ZDHHC18, ZDHHC20, ZFAND2A, ZFP1, ZFP36, ZFP37, ZFPM1, ZFX, ZFYVE1, ZGPAT, ZIC5, ZMIZ2, ZMYM3, ZMYND11, ZNF106, ZNF107, ZNF112, ZNF124, ZNF134, ZNF136, ZNF140, ZNF165, ZNF180, ZNF189, ZNF2, ZNF200, ZNF207,

			ZNF211, ZNF212, ZNF213, ZNF222, ZNF224, ZNF226, ZNF229, ZNF235, ZNF253, ZNF26, ZNF263, ZNF264, ZNF268, ZNF280A, ZNF282, ZNF292, ZNF296, ZNF304, ZNF317, ZNF318, ZNF350, ZNF37A, ZNF394, ZNF425, ZNF432, ZNF44, ZNF446, ZNF449, ZNF468, ZNF480, ZNF490, ZNF502, ZNF528, ZNF530, ZNF541, ZNF577, ZNF579, ZNF594, ZNF600, ZNF616, ZNF622, ZNF626, ZNF628, ZNF629, ZNF652, ZNF658, ZNF678, ZNF684, ZNF69, ZNF701, ZNF721, ZNF75A, ZNF771, ZNF776, ZNF789, ZNF79, ZNF805, ZNF808, ZNF837, ZNF841, ZNF844, ZNF846, ZNF93, ZSCAN12, ZSWIM6, ZSWIM7, ZSWIM8, ZXDC, ZZEF1
**c2**	regulation of macromolecule biosynthetic process(GO:0010556)	2.60E-13	ABLIM1, ACVR1, ADI1, ADNP2, AGO2, AIM2, AKAP17A, AMH, ARID3B, ARNTL2, ASCC2, ASCC3, ASPH, ASXL1, ATF6, ATP8B1, ATXN1L, BATF, BATF2, BATF3, BCL6, BCL9L, BCLAF1, BMPR2, BRMS1, BRMS1L, BRPF1, BRWD1, CALCOCO1, CAMKK2, CBFA2T2, CBX6, CCL2, CCL5, CCNK, CCNL1, CCRN4L, CD276, CDC5L, CDC6, CDKN1A, CDT1, CEBPE, CGA, CHD2, CHD4, CIC, CIR1, CIRBP, CNOT6, CNPY2, COPRS, CREB3, CREB3L2, CREB3L3, CREB5, CREBBP, CRK, CRTC2, CRY1, CRYM, CSRNP1, CSRNP2, CYLD, CYR61, DAPK3, DAXX, DDX58, DEDD2, DLX2, DMAP1, DPF2, DVL2, DYRK1B, ECM1, EFNA1, EGR1, EHMT2, EID2, EID3, EIF2AK3, EIF5, ELF4, EPAS1, EPC2, ESCO2, ESRRB, ETF1, ETS1, ETS2, F2RL1, FLCN, FLNA, FMR1, FNIP1, FNIP2, FOS, FOSB, FOSL2, FOXA3, FOXJ2, FOXJ3, FOXL2, FOXM1, FOXP1, FOXP4, FOXS1, FZD7, GATAD2A, GCFC2, GLMN, GON4L, GPS2, GRB10, GTF2IRD1, HBEGF, HBP1, HDAC10, HDAC5, HIPK1, HNF4A, HOXB6, HRAS, HSF2, HUS1, ID2, IFI16, IL11, IL1A, IL5, IL6R, INO80D, IRF2BP1, IRF7, IRS2, JMJD1C, JMY, JUN, JUP, KAT6A, KDM3A, KDM4C, KEAP1, KLF2, KLF4, KLF5, KRT17, LCORL, LGR4, LOXL2, LPIN1, LRPPRC, LZTR1, MACC1, MAFA,

MAP3K10, MAP3K13, MAPK7, MBD1, MDM2, MED10, MED15, MED26, MED29, MEF2A, MEF2D, MEIS3, MEPCE, MET, MNX1, MTF1, NAB1, NAB2, NDNL2, NEDD4L, NFAT5, NFATC3, NFKBIA, NFRKB, NFX1, NLK, NR1D2, NR1I3, NR2C2, NR2F2, NR3C1, NR4A1, NR6A1, NRBF2, PAF1, PASK, PDGFRB, PDPK1, PELI1, PER1, PER2, PGR, PHF19, PHTF2, PIAS1, PIK3R1, PKIG, PLAC8, PLAG1, PLAUR, PNRC1, POLR2A, POLR2L, POT1, PPAP2B, PPARD, PPM1A, PPP1R8, PPP3CA, PRDM10, PRICKLE1, PRKCI, PRMT2, PRRX2, PSPC1, PTK2B, PTOV1, QKI, RASD1, RCBTB1, RCOR3, RELA, RELB, RFX1, RFX3, RIPK1, RLF, RPRD1A, RPS6KA3, RSF1, RUNX1, RUNX2, RXRA, S1PR1, SAMD4B, SAMD8, SAP130, SAP30L, SCGB1A1, SERPINE1, SERTAD1, SERTAD2, SETD1A, SETD1B, SETD7, SGK1, SIN3A, SIRT5, SIX1, SKI, SKIL, SMAD6, SNIP1, SOHLH1, SP110, SP4, SP6, SQSTM1, SRFBP1, SRRT, SRSF10, SSX5, STAT1, STAT3, STK16, SUCO, SUPT5H, SYNCRIP, TAB3, TADA2B, TAF4, TAF7L, TBL1Y, TBX19, TCEAL7, TCEANC, TCEB3, TEAD4, TERF2, TFE3, TGFB1, THRA, THRAP3, THRB, TIA1, TIRAP, TNFAIP1, TNFRSF1A, TNIP1, TOB1, TOPORS, TP53BP1, TP53INP1, TP53INP2, TP73, TRAK1, TRIB1, TRIM11, TRIM21, TRIM24, TRIM25, TRIM27, TRIM29, TRIM5, TRMT6, TSC1, UBE2I, UBN1, UBTF, UIMC1, USP47, USP9X, VDR, VEZF1, VIMP, VPS36, WIBG, WRNIP1, WTIP, ZBTB1, ZBTB24, ZBTB26, ZBTB39, ZBTB41, ZBTB43, ZBTB45, ZBTB49, ZBTB5, ZBTB9, ZFP1, ZFP36, ZFP37, ZFPM1, ZFX, ZGPAT, ZMIZ2, ZMYND11, ZNF107, ZNF112, ZNF124, ZNF134, ZNF136, ZNF140, ZNF165, ZNF180, ZNF189, ZNF2, ZNF200, ZNF207, ZNF211, ZNF212, ZNF213, ZNF222, ZNF224, ZNF226, ZNF229, ZNF235, ZNF253, ZNF26, ZNF263, ZNF264, ZNF268, ZNF280A, ZNF282, ZNF292, ZNF296, ZNF304, ZNF317, ZNF318, ZNF350, ZNF37A, ZNF394, ZNF425, ZNF432, ZNF44, ZNF446, ZNF449, ZNF468,

			ZNF480, ZNF490, ZNF502, ZNF528, ZNF530, ZNF541, ZNF577, ZNF579, ZNF594, ZNF600, ZNF616, ZNF626, ZNF628, ZNF629, ZNF652, ZNF658, ZNF678, ZNF684, ZNF69, ZNF701, ZNF721, ZNF75A, ZNF771, ZNF776, ZNF789, ZNF79, ZNF805, ZNF808, ZNF837, ZNF841, ZNF844, ZNF846, ZNF93, ZSCAN12, ZXDC
**c2**	cytoplasm(GO:0005737)	1.94E-12	AARS2, ABLIM1, ACIN1, ACSS2, ACTA2, ADAM10, ADI1, ADM, ADRM1, AFTPH, AGAP1, AGO2, AIM2, AKAP12, ALAS1, ALDOC, ALG2, ALS2, AMBRA1, AMH, ANAPC16, ANKHD1, ANKLE2, ANKS1A, ANXA2, ANXA4, ANXA5, AP1G1, AP5Z1, APC, APPBP2, APPL1, AQP3, ARC, ARF3, ARFGEF2, ARFIP2, ARHGEF18, ARL5B, ARL6, ARL8B, ARNTL2, ARPC2, ASB6, ASCC3, ASPH, ATP6V1B2, ATP6V1C1, ATP6V1E1, ATRN, AVIL, AZI2, BAG3, BAIAP2, BATF, BBS9, BCAR1, BCAT1, BCLAF1, BFSP1, BHLHB9, BIN2, BIN3, BIRC7, BMF, BRAF, BRAP, BRMS1, BRPF1, BRWD1, C1orf116, C7orf49, CALCOCO1, CALM1, CAMKK2, CAPN2, CARD8, CASP2, CASP7, CASP8, CASS4, CC2D2A, CCL2, CCL5, CD2AP, CD44, CDC37L1, CDC42EP2, CDC42EP3, CDC42EP4, CDC5L, CDC6, CDK20, CDKL3, CDKN1A, CELF1, CELF4, CEP152, CEP170B, CEP97, CHAMP1, CHD4, CHERP, CHMP7, CIR1, CIRBP, CLCF1, CLN3, CNP, COPB1, CRBN, CREB3, CREB3L3, CREBBP, CRK, CRTC2, CRYM, CTSK, CWC22, CYHR1, CYP1A1, CYP27B1, CYP4F2, DAAM1, DAPK3, DAXX, DDB1, DDX58, DERA, DFNB31, DGKH, DGUOK, DHCR24, DIAPH2, DIDO1, DLG3, DMAP1, DNAJB9, DNMBP, DPF2, DPP3, DPYSL2, DSN1, DTX3L, DTX4, DUSP13, DVL2, EDC4, EGR1, EID3, EIF2AK3, EIF5, EML6, ENO2, ENSA, EPAS1, EPB41L1, ERCC1, ERCC6L2, ERRFI1, ESRP2, ETF1, ETNK1, ETS1, EXOSC2, FAM111A, FAM114A1, FAM213A, FAM89B, FANCC, FBXL20, FBXO32, FBXO4, FCRLA, FCRLB, FEM1A, FEM1B, FGD6, FIGNL1, FILIP1L,

FKBP1A, FLCN, FLNA, FMNL3, FMR1, FNIP1, FNIP2, FOXM1, FOXP1, FOXP4, FRS2, FSD1, FXR2, FZD7, GABBR1, GALK2, GAS2L1, GLRX, GM2A, GNMT, GNPDA1, GOLGA4, GPRASP1, GRB10, GSDMB, GTF2IRD1, GZMB, H6PD, HDAC10, HDAC5, HGS, HIF1AN, HIPK1, HNF4A, HNMT, HPS1, HRAS, HSF2, HSPA1A, HSPA1B, HSPA9, HYAL1, ID2, IFI16, IFIT3, IGFBP6, IL11, INPP5J, IP6K1, IPO9, IRF7, ITGA7, JMY, JUP, KATNAL1, KCTD11, KDM3A, KEAP1, KIAA0513, KIAA0753, KIAA1524, KLC3, KLHL21, KLHL24, KLHL25, KLHL41, KLHL5, KRT17, KRT8, LIMK2, LLGL2, LMNA, LPGAT1, LPIN1, LRIG2, LRPPRC, LSG1, MACC1, MAGEA11, MAP3K10, MAP3K12, MAPK7, MARCKSL1, MBD1, MCOLN3, MDM2, MEF2A, MEX3B, MICA, MICAL3, MLF1, MME, MOB1B, MPDZ, MSRB1, MT1G, MT2A, MTRF1L, MXD1, MYO7A, NAA11, NASP, NCF2, NDNL2, NDRG1, NDRG2, NDUFC2, NEDD4L, NEDD9, NEK7, NES, NFAT5, NFATC3, NFKBIA, NGEF, NGFR, NLK, NOL8, NOP58, NR1I3, NR3C1, NRBF2, NUDT16, NUMBL, NXF5, OAS3, OPTN, OXSR1, PALM3, PARD6B, PASK, PCBP1, PDCD2, PDGFRB, PDPK1, PDPR, PDXK, PEA15, PER1, PER2, PGR, PHF1, PIK3R1, PJA2, PKM, PLA2G16, PLAT, PLEKHA1, PLEKHM1, PMM2, POLH, PPAPDC1B, PPFIA1, PPHLN1, PPIG, PPP1R11, PPP1R12B, PPP1R8, PPP3CA, PPP4R2, PPP6R3, PRKCI, PRM3, PRMT2, PSMC2, PSPC1, PTGR1, PTK2B, PTMA, PTPN23, PTPN6, QKI, RAB17, RAB21, RAB22A, RAB26, RAB32, RAB33A, RAB33B, RAB38, RAB3D, RAB7A, RABL6, RALGPS2, RANBP10, RAPH1, RASA4, RASGRP3, RASSF3, RBM19, RC3H1, RCBTB1, RELA, RIN1, RIPK1, RNF213, RNF40, ROPN1L, RRAGB, RRAGC, RSRC1, RUNX2, S100A13, S100A14, S100A16, S100A6, SAMD4B, SAMD9, SART1, SAT2, SCGB1A1, SCYL3, SEC23IP, SELM, SERTAD2, SETX, SH2B2, SH2D1A, SH3BGRL3, SH3BP5, SHB, SHOC2, SIRT3, SKI, SKIL, SLC2A1,

			SLC30A3, SLC7A6OS, SLC7A8, SLC9A3R1, SLK, SLU7, SMAD6, SMG1, SMNDC1, SNAP29, SNIP1, SNX30, SOCS1, SOCS6, SOCS7, SOD3, SOHLH1, SPATA2, SPATS2, SPG20, SPRR3, SRP68, SRRT, SRSF10, SSH1, SSH2, STARD3, STAT1, STAT3, STK24, STON2, STXBP3, SWAP70, SYNCRIP, SYNRG, SYTL4, TAF4, TAF7L, TBC1D10B, TBC1D15, TBC1D2, TBCCD1, TDRKH, TERF2, TES, TESK2, TEX14, TFE3, TGFB1, THRA, TIA1, TIRAP, TJP2, TMEM259, TMSB15A, TMSB15B, TNFAIP1, TNIP1, TOB1, TOLLIP, TP53BP1, TPM1, TRAK1, TRIB1, TRIM11, TRIM2, TRIM21, TRIM23, TRIM24, TRIM25, TRIM27, TRIM29, TRIM5, TRIM56, TRIM68, TRO, TROAP, TROVE2, TSC1, TTLL7, TUBB2A, TUBB3, TUFT1, TWF1, TXLNA, TXNRD1, UBAP1, UBD, UBE2I, UBE2Z, UBQLN2, UBTD2, UBTF, UBXN1, UNKL, USP11, USP47, USP9X, UTRN, VASH2, VIL1, WASF1, WDR45B, WDR6, WHAMM, WIBG, WIPI2, XRN1, YKT6, ZC3HAV1, ZFAND2A, ZFP36, ZFPM1, ZFYVE26, ZFYVE9, ZMYND11, ZNF263, ZNF318, ZNF425, ZNF44, ZNF480, ZNF622
**c2**	negative regulation of biological process(GO:0048519)	3.50E-10	ABHD6, ACP5, ACVR1, ADAM10, ADM, ADNP2, AGO2, ALKBH1, AMBRA1, AMIGO2, ANKLE2, ANXA2, ANXA4, ANXA5, APC, APOD, ASPH, ASXL1, ATP2A2, ATP8B1, ATRAID, AVIL, AXL, BAG3, BCL6, BCL9L, BCLAF1, BIRC7, BMPR2, BRAF, BRAP, BRMS1, C5, C9orf89, CALCRL, CALM1, CARD8, CASP8, CBFA2T2, CBLL1, CBX6, CCK, CCL2, CCL5, CCNK, CD274, CD276, CD44, CD59, CD9, CDC6, CDKN1A, CDT1, CELF1, CGA, CHERP, CIR1, CIRBP, CLCF1, CLN3, CLTA, CNOT6, CNPY2, CRBN, CREB3, CREBBP, CRY1, CRYM, CYLD, CYP27B1, CYP4F2, CYR61, DACT3, DAPK3, DAXX, DDX58, DEDD2, DGUOK, DHCR24, DLG3, DLK1, DLX2, DMAP1, DNAJB2, DRD5, DUSP3, DYRK1A, ECM1, EFNA1, EFNB2, EGR1, EHMT2, EID2, EIF2AK3, ERCC1, ERRFI1, ESRRB, ETS1, F2RL1, F3, FAM111A, FAM89B, FEM1A, FIGNL1, FLCN,

	FLNA, FMR1, FNIP1, FNIP2, FOSB, FOXA3, FOXL2, FOXM1, FOXP1, FOXS1, FZD7, GAS2L1, GATAD2A, GBA, GCFC2, GLMN, GPC1, GPS2, GRB10, GSN, HBEGF, HBP1, HDAC10, HDAC5, HGS, HIF1AN, HIST1H4B, HIST1H4E, HNF4A, HRAS, HSPA1A, HSPA1B, HSPA9, HTR1D, HUS1, HYAL1, ID2, IER3, IFI16, IFI6, IFIT3, IGFBP6, IL11, IL1A, IL2RB, IL6R, INPP5J, IRF7, IRS2, JMY, JUN, JUP, KAT6A, KCNA5, KCNJ11, KCTD11, KDM3A, KIAA0226, KLF2, KLF4, LATS1, LEPROT, LGR4, LOXL2, LPIN1, LRP4, LRPPRC, LTBP3, LUC7L, MAOB, MAP3K10, MAP6D1, MAPK7, MBD1, MDM2, MED10, MED15, MEF2A, MEIS3, MEPCE, MIA3, MICB, MLF1, MPO, MT1G, MT2A, NAB1, NAB2, NBR1, NDRG2, NEDD4L, NES, NFKBIA, NFX1, NGEF, NGFR, NLK, NR1I3, NR2F2, NR6A1, NUDT16, NXN, OAS3, OSGIN1, PAF1, PALM3, PASK, PDPK1, PEA15, PELI1, PER1, PER2, PGLYRP4, PGR, PHF1, PHF19, PIAS1, PIK3R1, PINK1, PKIG, PLA2G16, PLAC8, PLAG1, PLAT, PLAUR, PLEKHA1, POT1, PPAP2B, PPARD, PPM1A, PPM1D, PPP3CA, PRICKLE1, PRKCI, PRMT2, PROCR, PSMC2, PTK2B, PTPN6, PTPRJ, RAB7A, RASA4, RASD1, RC3H1, RELA, RFX3, RHBDD1, RHEB, RIPK1, RND1, RNF43, RPS6KA3, RSF1, RTN4R, RUNX1, RUNX2, RXRA, RYR2, S1PR1, SAP130, SART1, SCGB1A1, SERPINE1, SERTAD2, SESN3, SETMAR, SIN3A, SIPA1, SIRT5, SIX1, SKI, SKIL, SLC25A4, SLC30A1, SLC9A3R1, SLFN11, SMAD6, SMPD1, SOCS1, SOCS4, SOCS5, SOCS6, SOCS7, SPG20, SPTAN1, SQSTM1, SRSF10, SRSF4, STAT1, STAT3, STK24, STXBP3, SUPT5H, SYNCRIP, SYTL4, TAC1, TBL1Y, TCEAL7, TERF2, TES, TEX14, TGFB1, THRA, THRB, TIA1, TIMP1, TIRAP, TMEM67, TMSB15A, TMSB15B, TNFAIP1, TNFRSF1A, TNFRSF9, TNIP1, TOB1, TOPORS, TP53INP1, TP73, TPM1, TRIB1, TRIM11, TRIM21, TRIM24, TRIM25, TRIM27, TRIM29, TRIM38, TRO, TSC1, TWF1, U2AF2, UBD, UBE2I, UBTF, UBXN1, UIMC1,

			USP47, USP9X, VDR, VIL1, VIMP, WDR6, WTIP, ZC3HAV1, ZFP36, ZFPM1, ZFYVE9, ZGPAT, ZMYND11, ZNF136, ZNF189, ZNF224, ZNF253, ZNF263, ZNF282, ZNF350, ZNF425
**c2**	nucleic acid binding(GO:0003676)	8.02E-09	AARS2, ACIN1, ADNP2, AEN, AGO2, AHDC1, AIM2, AKAP17A, ANKHD1, ANKHD1-EIF4EBP3, APLP2, ARID3B, ARNTL2, ASCC3, ASXL1, ATF6, ATXN1L, BATF, BATF2, BATF3, BCL6, BCLAF1, BRPF1, CALCOCO1, CBLL1, CBX6, CCDC81, CDC5L, CDT1, CEBPE, CELF1, CELF4, CENPBD1, CETN2, CHAMP1, CHD2, CHD4, CHERP, CIC, CIR1, CIRBP, CNNM3, CNOT6, CNP, CREB3, CREB3L2, CREB3L3, CREB5, CREBBP, CRY1, CSRNP1, CSRNP2, CSTF3, CWC22, DDB1, DDX58, DDX60L, DEDD2, DHX29, DHX34, DHX40, DLX2, DMAP1, DNASE1L1, DOT1L, EFTUD1, EGR1, EHD2, EIF2S2, EIF5, ELF4, ENDOD1, EPAS1, ERCC1, ERCC6L2, ESRP2, ESRRB, ETF1, ETS1, ETS2, EXOSC2, FAM200A, FAN1, FIP1L1, FMR1, FOS, FOSB, FOSL2, FOXA3, FOXJ2, FOXJ3, FOXL2, FOXM1, FOXP1, FOXP4, FOXS1, FUBP1, FXR2, GATAD2A, GCFC2, GIN1, GON4L, GPANK1, GPATCH1, GPATCH3, GSPT1, GTF2IRD1, HBP1, HBS1L, HDAC5, HELQ, HIST1H2AD, HIST1H2AE, HIST1H2AL, HIST1H2AM, HIST1H4B, HIST1H4E, HIST2H2BE, HMGB3, HMGXB3, HNF4A, HOXB6, HSF2, IFI16, IMP3, IRF7, ISG20, JUN, KAT6A, KCTD7, KDM3A, KIAA2018, KLF2, KLF3, KLF4, KLF5, LARP1B, LARP4, LCORL, LRPPRC, MAFA, MBD1, MBD6, MED26, MEF2A, MEF2D, MEIS3, MEX3A, MEX3B, MKRN1, MKRN3, MLF1, MNX1, MRPL1, MSL3, MTF1, MTRF1L, MXD1, MYNN, NFAT5, NFATC3, NFRKB, NFX1, NOL8, NOP58, NR1D2, NR1I3, NR2C2, NR2F2, NR3C1, NR4A1, NR6A1, NUDT16, NXF5, OAS3, PATL1, PCBP1, PDCD2, PER1, PGR, PHTF2, PIAS1, PLAG1, POLH, POLR2A, POLR2L, POT1, PPARD, PPP1R8, PRDM10, PRM3, PRRX2, PSPC1, QKI, RAD51B,

			RAD52, RAD54L2, RBM19, RBM23, RBM7, RBPMS2, RC3H1, RC3H2, RCAN3, RCOR3, RELA, RELB, RFX1, RFX3, RLF, RNASE1, RNASE4, RPP25, RSL1D1, RUNX1, RUNX2, RXRA, SAP30L, SCAF4, SCAF8, SETD1A, SETD1B, SETMAR, SETX, SF3B3, SF3B4, SIN3A, SIX1, SKIV2L, SLFN11, SLU7, SMAD6, SMNDC1, SOHLH1, SOS1, SP110, SP4, SP6, SREK1IP1, SRP68, SRRM1, SRRM2, SRRT, SRSF10, SRSF4, SRXN1, SSH1, SSH2, SSX5, STAT1, STAT3, SWAP70, SYNCRIP, SYNJ1, TADA2B, TAF13, TAF4, TAOK1, TBL1Y, TBX19, TCEANC, TCEB2, TCEB3, TDRKH, TEAD4, TERF2, TFE3, THRA, THRB, TIA1, TIGD7, TOPORS, TP53BP1, TP73, TRA2B, TRIM21, TRIM24, TRIM27, TRMT6, TROVE2, TSEN34, TUT1, U2AF2, UBN1, UBQLN2, UBTF, UHRF2, UNK, UNKL, VDR, VEZF1, WBP4, WIBG, WRNIP1, XRN1, ZBTB1, ZBTB24, ZBTB26, ZBTB39, ZBTB41, ZBTB43, ZBTB45, ZBTB49, ZBTB5, ZBTB9, ZC3HAV1, ZCCHC14, ZCCHC24, ZFP1, ZFP36, ZFP37, ZFPM1, ZFX, ZGPAT, ZIC5, ZMYM3, ZMYND11, ZNF107, ZNF112, ZNF124, ZNF134, ZNF136, ZNF140, ZNF165, ZNF180, ZNF189, ZNF2, ZNF200, ZNF207, ZNF211, ZNF212, ZNF213, ZNF222, ZNF224, ZNF226, ZNF229, ZNF235, ZNF253, ZNF26, ZNF263, ZNF264, ZNF268, ZNF280A, ZNF282, ZNF292, ZNF296, ZNF304, ZNF317, ZNF318, ZNF350, ZNF37A, ZNF394, ZNF425, ZNF432, ZNF44, ZNF446, ZNF449, ZNF468, ZNF480, ZNF490, ZNF502, ZNF528, ZNF530, ZNF541, ZNF577, ZNF579, ZNF594, ZNF600, ZNF616, ZNF622, ZNF626, ZNF628, ZNF629, ZNF652, ZNF658, ZNF678, ZNF684, ZNF69, ZNF701, ZNF721, ZNF75A, ZNF771, ZNF776, ZNF789, ZNF79, ZNF805, ZNF808, ZNF837, ZNF841, ZNF844, ZNF846, ZNF93, ZSCAN12, ZXDC
**c2**	cellular response to hormone stimulus(GO:0032870)	3.97E-08	ACSL6, ACVR1, APPL1, ASIP, ATP6V0D1, ATP6V0D2, ATP6V1B2, ATP6V1C1, ATP6V1D, ATP6V1E1, ATP6V1H, BAIAP2, BCAR1, CGA, CRK, CYP11A1, DENND4C, DUSP1, EGR1, ESRRB, FOS, FOXS1, FRS2, GAS2L1, GNG8, GRB10,

			HDAC5, HNF4A, HRAS, IRS2, KDM3A, LATS1, LPIN1, MDM2, NCF2, NR1D2, NR1I3, NR2C2, NR2F2, NR3C1, NR4A1, NR6A1, PDPK1, PGF, PGR, PIK3R1, PIK3R4, PKLR, PPARD, PPM1A, PRKCI, PXN, RHEB, RXRA, SH2B2, SOCS7, SOS1, STAT3, TGFB1, THRA, THRB, TNFRSF1A, TRIM24, TSC1, VDR, VIMP, ZNF106
**c2**	enzyme linked receptor protein signaling pathway(GO:0007167)	8.42E-07	ACVR1, ADAM10, AGO2, AMHR2, ANKS1A, APH1B, APPL1, ARHGEF18, ATP6V0D1, ATP6V0D2, ATP6V1B2, ATP6V1C1, ATP6V1D, ATP6V1E1, ATP6V1H, AXL, BAIAP2, BCAR1, BMPR2, BRAF, CALM1, CASP2, CCL2, CLTA, COL1A2, CRIM1, CRK, CSF1R, CSRNP1, DUSP3, EFNA1, EFNB2, EGR1, EIF2AK3, EPHB6, FIP1L1, FKBP1A, FOS, FOXS1, FRS2, GIGYF1, GRB10, GUCA1B, HBEGF, HGS, HRAS, IRS2, JUN, LIFR, LTBP3, MAPK7, MDM2, MEF2A, MET, NEDD4L, NFKBIA, NGEF, NGFR, NLK, NR4A1, PCSK6, PDGFRB, PDPK1, PGF, PIK3R1, PIK3R4, PLAT, PLEKHA1, PPM1A, PRKCI, PTK2B, PTPRF, PTPRJ, PXN, RALB, RELA, RHEB, RICTOR, RPS6KA3, RTN4R, RUNX2, RYR2, SERPINE1, SH2B2, SHCBP1, SHOC2, SKI, SKIL, SMAD6, SOCS5, SOCS7, SOS1, SQSTM1, STAT3, TGFB1, TSC1, USP9X, VIL1, ZFYVE9, ZNF106
**c2**	positive regulation of macromolecule metabolic process(GO:0010604)	2.58E-06	ABLIM1, ACVR1, AGO2, AMH, ANKLE2, APC, ARNTL2, ASPH, ASXL1, ATF6, BCL9L, BCLAF1, BMPR2, BRAF, BRCC3, BRMS1, BRPF1, C3, CALCOCO1, CALM1, CAMKK2, CASP8, CBFA2T2, CCK, CCL2, CCL5, CD276, CD44, CELF4, CGA, CIRBP, CLCF1, CNPY2, CREB3, CREB3L2, CREB5, CREBBP, CSF1R, CSRNP1, CSRNP2, CYR61, DDX58, DLX2, DNAJB2, DVL2, DYRK1A, DYRK1B, EFNA1, EGR1, ELF4, EPAS1, ESRRB, ETS1, ETS2, F2RL1, FBXO4, FKBP1A, FLCN, FNIP1, FNIP2, FOS, FOXA3, FOXJ2, FOXJ3, FOXL2, FOXM1, FOXP1, FOXS1, FUBP1, FZD7, GBA, GLMN, HDAC5, HNF4A, HRAS, HSF2, ID2, IER3,

			IFI16, IL11, IL1A, IL5, IL6R, IRF7, IRS2, IST1, JUN, JUP, KAT6A, KDM3A, KLF2, KLF4, KLF5, KRT17, LATS1, LGR4, LPCAT1, LPIN1, MAFA, MAPK7, MDM2, MED10, MEF2A, MEF2D, MET, MTF1, NDNL2, NFAT5, NFATC3, NFKBIA, NPTN, NR2C2, NR2F2, NR4A1, PAF1, PASK, PDGFRB, PELI1, PELI2, PHF1, PHF19, PIAS1, PIK3R1, PLAC8, PLAG1, POLR2A, POLR2L, POT1, PPAP2B, PPARD, PPM1A, PPP3CA, PRICKLE1, PRMT2, PSMC2, PTK2B, QKI, RELA, RELB, RFX3, RHBDD1, RICTOR, RIPK1, RLF, RPS6KA3, RSF1, RUNX1, RUNX2, RXRA, S1PR1, SERPINE1, SERTAD1, SERTAD2, SIN3A, SIX1, SKI, SMPD1, SOCS5, SOHLH1, SQSTM1, STAT1, STAT3, STK16, SUCO, SUPT5H, TAF4, TBL1Y, TBX19, TCEB2, TCEB3, TEAD4, TERF2, TFE3, TGFB1, THRA, THRAP3, THRB, TIRAP, TNC, TNFAIP1, TNFRSF1A, TNIP1, TOLLIP, TOPORS, TP53BP1, TP53INP1, TP53INP2, TP73, TRA2B, TRIB1, TRIM24, UBTF, UIMC1, VDR, WIBG, ZC3HAV1, ZFP36, ZFPM1, ZMIZ2, ZNF292, ZXDC
**c2**	nuclear body(GO:0016604)	2.87E-06	ACIN1, AKAP17A, CCNL1, CDC5L, CIR1, CREB3, CREBBP, DAPK3, DAXX, DYRK1A, ELF4, GATAD2A, HDAC5, HIPK1, HSPA1A, HSPA1B, IFI16, ISG20, KAT6A, LSG1, MAPK7, MBD1, MDM2, NOP58, PATL1, PIAS1, PPIG, PPP1R8, PRPF18, PRPF4, PSPC1, RSRC1, SART1, SETD1A, SETD1B, SKI, SKIL, SLU7, SMC6, SMNDC1, SRRM1, SRRM2, SRSF10, SRSF4, TOLLIP, TOPORS, TP53INP1, TP53INP2, TRIM27, TUT1, UBE2I, UBN1, WBP4
**c2**	response to inorganic substance(GO:0010035)	3.05E-06	ALG2, ALOX5AP, APOD, AQP3, ASPH, ATP5D, BDH1, BRAF, CALM1, CASP8, CDKN1A, CYP11A1, CYP1A1, DUSP1, EGR1, ETS1, FANCC, FOS, FOSB, GSN, HVCN1, HYAL1, ID2, IL1A, JUN, KCNK3, KLF2, LOXL2, MAOB, MAPK7, MDM2, MEF2A, MPO, MT1G, MT2A, MTF1, NDRG1, NEDD4L, NFATC3, PDGFRB, PKLR, PLEKHA1, PPP3CA, PTK2B, PXN, RELA, RYR2, S100A13, S100A16, SCGB1A1, SLC30A1, SOD3, SRRT, TPM1, TXNRD1

**c2**	ligand-dependent nuclear receptor activity(GO:0004879)	4.73E-06	ESRRB, HNF4A, NR1D2, NR1I3, NR2C2, NR2F2, NR3C1, NR4A1, NR6A1, PGR, PPARD, PTGER3, RXRA, STAT3, THRA, THRB, VDR
**c2**	endosome(GO:0005768)	8.62E-06	ALS2, ANXA2, AP1G1, ARC, ARFGEF2, ARPC2, ATP11A, ATP6V0D1, ATP6V0D2, ATP6V1E1, ATP9A, AVL9, CALCRL, CLN3, CLVS1, CTNS, DGKH, DIAPH2, FRS2, GPC1, HGS, KIAA0226, KIAA0368, KIAA1324, LAMP1, LAMP2, LDLR, LEPROT, LMTK2, NBR1, NGFR, PI4K2A, PIK3R4, PLD1, PRKCI, PTPN23, RAB17, RAB21, RAB22A, RAB7A, RASGEF1B, RILP, SLC30A3, SQSTM1, STARD3, SYTL4, TFRC, TNFAIP1, TOM1, TRAK1, VPS18, VPS36, VPS41, WASF2, YKT6, ZFYVE9
**c2**	activation of innate immune response(GO:0002218)	2.67E-05	AIM2, CASP8, CNPY3, CTSK, CYLD, DUSP3, FOS, IFI16, IRF7, ITGB2, JUN, LY96, MAPK7, MEF2A, NFKBIA, PELI1, PELI2, PIK3R4, RELA, RIPK1, RPS6KA3, S100A14, SIN3A, TAB3, TIRAP, TNIP1, TRIM5
**c2**	sequence-specific DNA binding transcription factor activity(GO:0003700)	2.96E-05	ADNP2, AFF4, ARNTL2, ATF6, BATF, BATF2, BATF3, BCL6, CBFA2T2, CCRN4L, CEBPE, CIR1, CREB3, CREB3L2, CREB3L3, CREB5, CREBBP, CSRNP1, CSRNP2, DLX2, EGR1, ELF4, EPAS1, ESRRB, ETS1, ETS2, FOS, FOSB, FOSL2, FOXA3, FOXJ2, FOXJ3, FOXL2, FOXM1, FOXP1, FOXP4, FOXS1, FUBP1, GATAD2A, GCFC2, GTF2IRD1, HNF4A, HOXB6, HSF2, IRF7, JUN, KDM3A, KLF2, KLF3, KLF4, KLF5, LZTR1, MAFA, MBD1, MEF2A, MEF2D, MEIS3, MNX1, MSL3, MTF1, MXD1, MYNN, NFAT5, NFATC3, NFX1, NR1D2, NR1I3, NR2C2, NR2F2, NR3C1, NR4A1, NR6A1, PGR, PHF1, PLAG1, PLAUR, PPARD, PRRX2, RELA, RELB, RFX1, RFX3, RUNX1, RUNX2, RXRA, SIN3A, SIX1, SMAD6, SOHLH1, SP4, STAT1, STAT3, STK16, TAF13, TAF4, TBX19, TEAD4, TFE3, THRA, THRB, TP73, TRIM25, TRIM29, UBN1, VDR, ZFP37, ZFPM1, ZGPAT, ZIC5, ZNF134, ZNF140, ZNF165, ZNF189, ZNF207, ZNF213, ZNF263, ZNF268, ZNF292, ZNF37A, ZNF394, ZNF446, ZNF449, ZNF628, ZNF69, ZNF93,

			ZSCAN12, ZXDC
**c2**	regulation of macrophage chemotaxis(GO:0010758)	4.11E-05	C3AR1, C5, CCL2, CCL5, PTK2B, PTPRJ
**c2**	intracellular signal transduction(GO:0035556)	4.14E-05	ADM, AEN, AGAP1, AGO2, ALS2, ARF3, ARFGEF2, ARFIP2, ARHGAP12, ARHGAP23, ARHGAP25, ARHGAP28, ARHGEF18, ARL16, ARL5B, ARL6, ARL8B, ASB6, ASPH, AXL, AZI2, BATF, BCAR3, BCL6, BRAF, BRAP, CALCRL, CALM1, CAMKK2, CASP2, CCL2, CCL5, CDC42EP4, CDKN1A, CLCF1, COL1A2, CSF1R, DAPK3, DEDD2, DGKH, DHCR24, DNMBP, DOCK9, DRD5, DUSP1, DUSP3, DVL2, EIF2AK3, FLCN, FLNA, FNIP1, FNIP2, FOS, FOXM1, FRS2, FZD7, GEM, GPS2, GSN, HBEGF, HIPK1, HIST1H4B, HIST1H4E, HRAS, HRH2, HTR1D, ICK, IFI16, IFT27, INPP5F, IRS2, JUN, KSR1, LATS1, LY96, MAGI3, MAP3K10, MAP3K12, MAP3K13, MAPK7, MC2R, MC3R, MDM2, MEF2A, MOB1B, NDRG1, NGEF, NLK, NR4A1, OXSR1, PCLO, PDGFRB, PDPK1, PI4KA, PIAS1, PIK3R1, PINK1, PLD1, PLEKHA1, PLEKHM1, PPP3CA, PRKCI, PSMC2, PTGER3, PTK2B, PTPN6, RAB17, RAB21, RAB22A, RAB26, RAB27B, RAB32, RAB33A, RAB33B, RAB38, RAB3D, RAB7A, RABL6, RALB, RALGPS2, RAPGEF2, RASA4, RASD1, RASGEF1B, RASGRP3, RCAN3, RHEB, RHOBTB1, RHOBTB2, RHOF, RICTOR, RND1, RPS6KA3, RRAD, RRAGB, RRAGC, RYR2, S1PR1, S1PR3, SH2B2, SH3BP5, SHOC2, SIPA1, SLC9A3R1, SNIP1, SOCS1, SOCS4, SOCS5, SOCS6, SOCS7, SOS1, SPSB3, SQSTM1, STAT1, STAT3, SYDE2, TAB3, TEAD4, TGFB1, TIRAP, TJP2, TOLLIP, TOPORS, TP73, TRIB1, TRIM23, WASF2, ZFP36
**c2**	autophagic vacuole(GO:0005776)	5.07E-05	AMBRA1, ATG14, ATG16L1, CLN3, NBR1, OSBPL7, SQSTM1, TP53INP1, TP53INP2, WIPI2
**c2**	regulation of cellular component	5.85E-05	ABTB2, ACSL6, ADAM10, ADNP2, AIM2, ANXA2, APC, APOD, APPL1, ARC,

	organization(GO:0051128)		ARHGEF18, ARHGEF19, ARPC2, AVIL, BAIAP2, BCAR1, BCL6, BCL9L, BCLAF1, BMF, BMPR2, BRWD1, C3, CALY, CBFA2T2, CBLL1, CCK, CCL2, CDC42EP2, CDC42EP3, CDC42EP4, CDC6, CDKN1A, CNOT6, COL5A1, CRBN, CREB3, CRIM1, CRK, CSF1R, CYLD, CYP27B1, CYR61, DACT3, DAPK3, DGUOK, DNAJB2, EFNA1, ERCC1, ETF1, ETS1, EXOSC2, F2RL1, FBLIM1, FGD6, FLCN, FMNL3, FNBP1L, FOXM1, GAS2L1, GPRC5B, GSN, HBEGF, HNF4A, HRAS, HSPA1A, HSPA1B, HYAL1, IGFBP6, IL1A, IL5, INPP5J, ITGA7, ITGB2, JMY, JUP, KDM3A, KIAA0226, KIAA1324, KLHL41, KRT17, LARP4, LATS1, LPIN1, LRPPRC, MAP6D1, MEF2A, NEDD4L, NES, NGEF, NGFR, NOL8, NPTN, NRCAM, NUMBL, OSGIN1, PDGFRB, PDPK1, PHF1, PHF19, PIK3R1, PINK1, POT1, PPM1A, PRKCI, PTK2B, PTPRJ, RAB17, RALB, RICTOR, RPS6KA3, RTN4R, S100A13, S1PR1, SDC2, SDC4, SERPINE1, SERTAD2, SGK1, SIPA1, SIX1, SKI, SKIL, SMAD6, SPG20, SPTAN1, SSH1, SSH2, STAT1, STK24, STON2, SURF4, TAC1, TAOK1, TBCCD1, TERF2, TEX14, TGFB1, THRA, TMEM67, TMSB15A, TMSB15B, TPM1, TRO, TSC1, TWF1, UBD, USP47, VIL1, WTIP, ZMYM3
**c2**	nucleoplasm(GO:0005654)	7.26E-05	AEN, ATF6, CALM1, CASC5, CASP7, CCRN4L, CDC6, CDKN1A, CDT1, CIRBP, CREBBP, CSTF3, DDB1, DUSP1, DUSP3, DUSP5, DYRK1A, EGR1, ENSA, EPAS1, ERCC1, ESCO2, ESRRB, FANCB, FANCC, FANCD2, FMR1, FOS, HDAC10, HDAC5, HIST1H4B, HIST1H4E, HNF4A, HUS1, IFI16, IP6K1, IPMK, IRF7, ISG20, JMJD1C, JUN, KRT8, LMNA, LRPPRC, MAPK7, MAU2, MDC1, MDM2, MED10, MED15, MED26, MEF2A, NEDD4L, NGFR, NR1D2, NR1I3, NR2C2, NR3C1, NR4A1, NR6A1, NRBF2, NRBP1, NUDT16, NUP214, NUP98, OPTN, PCBP1, PCF11, PDPK1, PGR, PIAS1, POLH, POLR2A, POLR2L, POT1, PPARD, PPIG, PPM1A, PPP1R12B, PRPF4, PSMC2, PSPC1, RAD51B, RAD52,

			RBM19, RELA, RPS6KA3, RSF1, RXRA, SART1, SETX, SF3B3, SF3B4, SIN3A, SKI, SKIL, SQSTM1, SRRM1, SRRT, SRSF10, SRSF4, STAT1, STAT3, STK24, SUPT5H, SYNCRIP, TAF13, TAF4, TATDN2, TCEB2, TCEB3, TEAD4, TERF2, THRA, THRB, TJP2, TP53BP1, TRIM27, U2AF2, UBTF, VDR, WIBG, WTAP, ZFPM1
**c2**	intracellular(GO:0005622)	1.15E-04	AGAP1, ALS2, ANO7, ANXA5, APOD, ARF3, ARHGAP12, ARHGAP23, ARHGAP25, ARHGAP28, ARHGEF19, ARID3B, ARL16, ARL5B, ARL6, ARL8B, ASCC3, BCAR3, C6orf62, CAMKK2, CAPN2, CARD6, CARD8, CASP2, CETN2, CSTF3, DHX40, DNMBP, DVL2, EFR3A, EID2, ENO2, FAM49A, FGD6, FNIP1, FSD1, FSD1L, GAD1, GEM, GLMN, GSPT1, HIST1H2AD, HIST1H2AE, HIST1H2AL, HIST1H2AM, HIST2H2BE, HRAS, IFT27, IMP3, ISG20, JMJD1C, KSR1, MOB3A, NEDD4L, NES, NGEF, NR3C1, PGLYRP4, PIK3R1, PITPNM1, RAB17, RAB21, RAB22A, RAB26, RAB27B, RAB32, RAB33A, RAB33B, RAB38, RAB3D, RAB7A, RABL6, RALB, RALGPS2, RAPGEF2, RASA4, RASD1, RASGEF1B, RASGRP3, RHEB, RHOBTB1, RHOBTB2, RHOF, RND1, RNF114, RRAD, RRAGB, SCGB1A1, SLC2A1, SMAD6, SMC6, SOS1, SRGAP2, SSX5, SYDE2, TBC1D10B, TBC1D10C, TBC1D15, TBC1D17, TBC1D19, TBCK, TNIP1, TOM1, TRIM11, TRIM15, TRIM2, TRIM21, TRIM23, TRIM24, TRIM27, TRIM29, TRIM38, TRIM5, TRIM52, TRIM56, TRIM68, TTPAL, TUBB3, TWF1, WASF2, XRN1, ZFP1, ZFP37, ZNF112, ZNF124, ZNF136, ZNF140, ZNF180, ZNF189, ZNF2, ZNF211, ZNF212, ZNF213, ZNF222, ZNF224, ZNF226, ZNF229, ZNF235, ZNF253, ZNF26, ZNF263, ZNF264, ZNF268, ZNF282, ZNF304, ZNF317, ZNF350, ZNF37A, ZNF394, ZNF425, ZNF432, ZNF44, ZNF446, ZNF468, ZNF480, ZNF490, ZNF528, ZNF530, ZNF577, ZNF616, ZNF626, ZNF658, ZNF678, ZNF684, ZNF69, ZNF701, ZNF721, ZNF75A, ZNF776, ZNF789, ZNF79, ZNF805,

			ZNF808, ZNF841, ZNF844, ZNF846, ZNF93
**c2**	phospholipid metabolic process(GO:0006644)	1.29E-04	ACSL6, AGPAT3, ARF3, CEPT1, CHKA, CLN3, CSF1R, DPM3, ETNK1, FAM135A, GGPS1, INPP4A, INPP5F, INPP5J, IP6K1, ISYNA1, LIPG, LPCAT1, LPGAT1, LPIN1, MBOAT2, PCYT1A, PDGFRB, PGS1, PI4K2A, PI4KA, PIGC, PIGQ, PIGU, PIGV, PIK3R1, PIK3R4, PIP4K2A, PLA2G16, PLAA, PLD1, PPAP2B, SAMD8, SGMS2, SLC44A1, SLC44A5, SMG1, SMPD1, SMPDL3B, SYNJ1
**c2**	response to laminar fluid shear stress(GO:0034616)	1.57E-04	ETS1, KLF2, MAPK7, NCF2, SMAD6, TGFB1
**c2**	membrane-bounded vesicle(GO:0031988)	1.61E-04	ACRBP, ADAM10, ANXA2, ANXA5, AP1G1, AP1G2, APH1B, ARC, ARFGEF2, ATP6V0D1, ATP6V1B2, BMF, C3, CASC5, CD274, CLCA1, CLN3, CLTA, CLVS1, CNP, CNST, COL1A2, COPB1, DENND4C, DVL2, ENTHD2, FLNA, FNBP1L, GPRC5B, GSN, HGS, HPS1, IDUA, IST1, KIAA0368, LAMP1, MAP6D1, MME, MPO, MYO7A, NCF2, NEU1, PCLO, PDGFRB, PDPK1, PI4K2A, PI4KA, PKM, PLAA, PLAT, PLEKHF2, PRG2, PTPN23, RAB17, RAB22A, RAB27B, RAB32, RAB38, RAB3D, RAB7A, RPH3A, SCGB1A1, SLC1A4, SLC2A1, SLC30A3, SLC32A1, SMPD1, SOCS1, SRGAP2, STK16, STON2, STX1A, STX3, STXBP3, SYN1, SYNGR1, SYT11, SYTL4, TBC1D2, TFRC, TGFB1, TIRAP, TPRG1L, VPS41
**c2**	L-alanine transport(GO:0015808)	1.91E-04	SLC1A4, SLC36A1, SLC36A4, SLC38A7
**c2**	glucosylceramide metabolic process(GO:0006678)	1.91E-04	CLN3, GBA, GBA2, UGCG
**c2**	regulation of biological quality(GO:0065008)	2.05E-04	ABHD6, ACP5, ACTA2, ADM, ALAS2, ALS2, ANXA2, ANXA5, APC, APLP2, ARC, ARHGEF18, ARPC2, ASPH, ATP11A, ATP2A2, ATP2B1, ATP6V0D1, ATP6V0D2, ATP6V1B2, ATP6V1C1, ATP6V1D, ATP6V1E1, ATP6V1H, ATP8B1,

			ATP9A, AVIL, AXL, BAIAP2, BAIAP3, BCAR1, BMPR2, BRPF3, BRWD1, C3AR1, C5, CACNB3, CALM1, CASC5, CCK, CCL2, CCL5, CCR10, CD244, CD52, CD59, CD9, CDC42EP2, CDC42EP3, CDC42EP4, CELF4, CGA, CHAMP1, CHERP, CLCN6, CLN3, CNNM2, CNP, COL1A2, CREB3, CREBBP, CRK, CRTC2, CRYM, CSF1R, CTSK, CYP11A1, CYP1A1, CYP27B1, CYP4F2, DCLRE1C, DEDD2, DFNB31, DGKH, DHRS9, DOCK9, DPM3, DRD5, EGR1, EHD2, EIF2AK3, EPAS1, EPHB6, ESAM, F2RL1, F2RL2, F3, FANCC, FBLIM1, FBXO4, FGD6, FGFBP3, FLNA, FLVCR1, FMNL3, FOXA3, FOXS1, FTO, GAD1, GLRX, GLS, GSN, GUCA1B, HIST1H4B, HIST1H4E, HNF4A, HOXB6, HPS1, HPS6, HRAS, HRH2, HTR1D, ID2, IDUA, IER3, IL1A, IRS2, ITGA4, ITGA7, ITGB2, JMJD1C, JMY, JUN, JUP, KCNA5, KCNE2, KCNJ11, KCNK3, KEL, KIF3A, KIF3B, KLF2, LAMP2, LATS1, LDLR, LIPG, LPCAT1, MAFA, MC3R, MDM2, MEF2A, MET, MME, MMP1, MT2A, NCF2, NEDD4L, NFKBIA, NGFR, NLRP5, NPC2, NPTN, NR3C1, NXN, PCLO, PCSK6, PDGFRB, PDK3, PDPK1, PIK3R1, PLAT, PLAUR, PLEKHA1, POT1, PPAP2B, PPP3CA, PQLC2, PROCR, PTGER3, PTK2B, PTPN6, PTPRJ, RAB38, RAB7A, RAD51B, RAPGEF2, RICTOR, RYR2, S100A13, S100A14, S1PR1, S1PR3, SCPEP1, SERPIND1, SERPINE1, SFXN3, SGK1, SH2B2, SH3BGRL3, SHBG, SIN3A, SKIL, SLC1A3, SLC30A1, SLC32A1, SLC4A1, SLC7A7, SLC7A8, SLC9A3R1, SLC9A6, SMC6, SNPH, SOS1, SPG20, SPTAN1, SSH1, SSH2, STARD3, STAT3, STIM2, STRA6, STX1A, STXBP3, SYN1, SYNGR1, TAC1, TBCCD1, TERF2, TEX14, TFRC, TGFB1, THRA, THRB, TIMP1, TMSB15A, TMSB15B, TNIP1, TOPORS, TP73, TPM1, TRIM21, TRIM24, TSC1, TWF1, TXNDC15, TXNRD1, VDR, VIL1, VIMP, ZFPM1, ZSWIM7
**c2**	regulation of vascular endothelial growth factor	2.71E-04	C3, C3AR1, C5, CCL2, HPSE, IL1A, NDRG2, TGFB1

	production(GO:0010574)		
**c2**	negative regulation of receptor activity(GO:2000272)	3.12E-04	ERRFI1, PTPRJ, SOCS4, SOCS5, ZGPAT
**c2**	lysosome(GO:0005764)	3.20E-04	ACP5, AGA, AKR1B10, ARSA, CALCRL, CLN3, CTNS, CTSA, CTSK, GM2A, GNS, HPS1, HPSE, HYAL1, IDUA, KCNE2, KIAA0226, KIAA1324, LAMP1, LAMP2, LDLR, MAN2B2, MANBA, MPO, NBR1, NCF2, NEU1, NPC2, RAB7A, RILP, RRAGB, RRAGC, SLC36A1, SQSTM1, STX3, VPS36
**c2**	phosphotransferase activity, alcohol group as acceptor(GO:0016773)	3.60E-04	ACVR1, AMHR2, AXL, BMP2K, BMPR2, BRAF, CAMKK2, CASS4, CCL2, CCL5, CCNK, CDK19, CDK20, CDKL3, CDKN1A, CHKA, CLK4, CRIM1, CSF1R, DAPK3, DGKH, DGUOK, DYRK1A, DYRK1B, EFNA4, EIF2AK3, EPHB6, ETNK1, FASTKD2, FASTKD5, FIP1L1, FKBP1A, GALK2, HIPK1, HSPA9, ICK, IP6K1, IPMK, ITPKC, KSR1, LATS1, LIMK2, LMTK2, MAP3K10, MAP3K12, MAP3K13, MAPK7, MET, MLKL, NAGK, NEK7, NLK, NRBP1, OXSR1, PAK4, PASK, PDGFRB, PDK3, PDPK1, PDXK, PI4K2A, PI4KA, PIK3R4, PINK1, PIP4K2A, PKLR, PKM, POLR2A, POLR2L, PRKCI, PTK2B, RIPK1, RPS6KA3, SCYL3, SGK1, SLK, SMG1, SQSTM1, STK16, STK24, TAOK1, TBCK, TESK1, TESK2, TEX14, TP53RK, TRIB1, TRIM24, TRIM27, TWF1, XYLB
**c2**	cytoplasmic vesicle(GO:0031410)	4.28E-04	ACRBP, ADAM10, AMBRA1, ANXA2, AP1G1, AP1G2, APH1B, ARC, ARFGEF2, ATP6V0D1, ATP6V1B2, ATP6V1C1, BMF, CALY, CASC5, CLCA1, CLN3, CLTA, CLVS1, CNP, CNST, COPB1, DENND4C, DVL2, ENTHD2, FNBP1L, HGS, HPS1, HYAL1, IDUA, IST1, KIAA0368, LAMP1, MAP6D1, MME, MPDZ, MPO, MYO7A, NBR1, NCF2, NEU1, PCLO, PDGFRB, PDPK1, PI4K2A, PI4KA, PLAT, PLEKHF2, PRG2, PTPN23, RAB17, RAB22A, RAB27B, RAB32, RAB38, RAB3D, RAB7A, RPH3A, SCGB1A1, SLC1A4, SLC2A1, SLC30A3, SLC32A1, SMPD1, SOCS1, SQSTM1, SRGAP2, STK16, STON2, STX1A, STX3,

			STXBP3, SYN1, SYNGR1, SYT11, SYTL4, TBC1D2, TFRC, TGFB1, TIRAP, TP53INP1, TP53INP2, TPRG1L, VPS41
**c2**	response to antibiotic(GO:0046677)	4.34E-04	CASP8, CCL2, CYP11A1, CYP1A1, EGR1, ETS1, HYAL1, MDM2, RSRC1, SKIL, SLC1A3
**c2**	response to lipopolysaccharide(GO:0032496)	4.95E-04	ACP5, ADM, AXL, CASP8, CCL2, CCL5, CEBPE, CNP, CYP11A1, CYP1A1, CYP27B1, FOS, JUN, LY96, MAOB, MEF2A, NCF2, NFKBIA, PAF1, PALM3, PELI1, PTGER3, RELA, RPS6KA3, S100A14, SCGB1A1, SERPINE1, STAT1, TAC1, TGFB1, TIRAP, TNFRSF1A, TRIB1, VIMP
**c2**	Rap GTPase activator activity(GO:0046582)	5.11E-04	RAPGEF2, RASGRP3, SIPA1
**c2**	positive regulation of toll-like receptor 3 signaling pathway(GO:0034141)	5.11E-04	F2RL1, PELI1, TIRAP
**c2**	macroautophagy(GO:0016236)	5.36E-04	CLN3, KIAA1324, NBR1, SQSTM1
**c2**	organelle organization(GO:0006996)	6.18E-04	ABLIM1, ACIN1, ALS2, ANAPC16, ANK1, ANLN, APC, ARC, ARFGEF2, ARFIP2, ARHGEF18, ASIP, ASXL1, ATG14, ATG16L1, ATP6V0D1, ATP6V0D2, ATP6V1B2, ATP6V1C1, ATP6V1D, ATP6V1E1, ATP6V1H, AVIL, BAIAP2, BCAR1, BCL6, BIN3, BRCC3, BRPF1, BRPF3, BRWD1, CALY, CASC5, CASP7, CBX6, CCK, CCL2, CCNK, CCSER2, CD2AP, CDC42EP2, CDC6, CEP152, CETN2, CHAMP1, CHD4, CLN3, CLVS1, CNP, COPB1, COPRS, CREBBP, DAAM1, DAPK3, DCLRE1C, DIAPH2, DMAP1, DOPEY2, DOT1L, DPYSL2, DTX3L, EHD2, EHMT2, EIF2AK3, ENSA, EPAS1, EPB41L1, EPC2, ERCC1, ETS1, FAM160A2, FANCD2, FBXO4, FGD6, FLNA, FMNL3, FNBP1L, FOXA3, FOXP1, FSD1, GAS2L1, GSN, HDAC10, HDAC5, HIST1H2AD, HIST1H2AE, HIST1H2AL, HIST1H2AM, HIST1H4B, HIST1H4E, HIST2H2BE, HPS1, HPS6,

			HRAS, IDUA, IFI6, JMJD1C, JMY, JUN, JUP, KAT6A, KDM3A, KDM4C, KIF13A, KIF3A, KIF3B, KLHL21, KLHL41, KRT17, KRT19, KRT8, KRT9, LARP4, LATS1, LMNA, LOXL2, LPIN1, MAP1A, MAP3K12, MEF2A, MICAL3, MIS12, MSL2, MSL3, MUM1, MYO7A, NASP, NCF2, NEDD9, NEK7, NR3C1, NUP98, OPTN, PAF1, PAK4, PCLO, PDGFRB, PDPK1, PEX16, PHF1, PHF19, POT1, PRKCI, PRM3, PRMT2, PTK2B, PXN, RAB22A, RAB32, RAB7A, RANBP10, RCBTB1, RHOF, RICTOR, RLF, RND1, RNF40, RSF1, SAP130, SEC23IP, SEC24A, SEC24B, SETD1A, SETD1B, SETD7, SETMAR, SH2B2, SIPA1, SLC25A4, SLC9A3R1, SMC6, SMCHD1, SNAP29, SPG20, SSH1, SSH2, SUPT5H, SURF4, TBCCD1, TCAP, TERF2, TESK2, TEX14, TMED5, TMSB15A, TMSB15B, TNFAIP1, TP53INP2, TP73, TPM1, TUBA1A, TUBB3, UBE2I, UBN1, UIMC1, USP9X, VIL1, VPS18, WASF1, WASF2, WDR45B, WIPI2, WTIP, ZFPM1, ZMYM3, ZMYND11
**c2**	cytosol(GO:0005829)	6.57E-04	ACIN1, ACP5, ACSS2, ACTA2, ADI1, AFTPH, AGO2, AIM2, AKR1B10, ALDOC, ALOX5AP, ALS2, ANK1, ANO7, ANXA9, AP1G1, AP1S2, AP1S3, APC, APPL1, ARFGEF2, ARHGAP12, ARHGAP23, ARHGAP25, ARHGAP28, ARHGEF18, ARPC2, ATP6V1B2, ATP6V1C1, ATP6V1D, ATP6V1E1, ATP6V1H, BAG3, BAIAP2, BCAR1, BCAT1, BMF, BRAF, BRPF3, C9orf89, CACNB3, CALM1, CASC5, CASP2, CASP7, CASP8, CDC6, CDKN1A, CDT1, CEP152, CETN2, CHKA, CHMP4C, CHMP7, CLTA, CMBL, CNOT6, COPB1, CREB3, CRK, CYLD, DAPP1, DAXX, DDX58, DENND4C, DGUOK, DHCR24, DIAPH2, DOCK9, DPYSL2, DSN1, DUSP3, DVL2, EDC4, EIF2S2, EIF5, ENO2, ENTHD2, EPAS1, EPB41L1, ETF1, ETNK1, EXOSC2, FANCC, FBLIM1, FBXL3, FKBP1A, FLNA, GBE1, GBP2, GGPS1, GLRX, GLS, GRB10, GSN, GZMB, HDAC5, HGS, HIF1AN, HPS6, HRAS, HSPA1A, HSPA1B, ICK, ID2, IFI16, IFIT3, IL1A,

			INPP4A, INPP5J, IP6K1, IRF7, IRS2, IST1, ISYNA1, ITPKC, JUN, JUP, KCTD5, KCTD7, KIF3A, KIF3B, LATS1, LMNA, LPIN1, LSG1, MAP1A, MAP3K12, MAP3K7CL, MAPK7, MDM2, MIS12, MOB1B, MT2A, MYO7A, NAT2, NBR1, NCF2, NDRG1, NDRG2, NEDD4L, NFATC3, NFKBIA, NGEF, NGFR, NLRP5, NNMT, NR1I3, NR3C1, NUP214, NUP98, NXN, OAS3, OPTN, OSBPL7, PARD6B, PARD6G, PATL1, PCYT1A, PDPK1, PDXK, PELI1, PELI2, PI4K2A, PI4KA, PIK3R1, PIK3R4, PINK1, PIP4K2A, PKLR, PKM, PLA2G16, PMM2, PPM1A, PPM1B, PPP3CA, PPP3CC, PRICKLE1, PRKCI, PRMT2, PSMC2, PTK2B, PTPN6, RANBP10, RASA4, RELA, RELB, RHEB, RHOBTB1, RHOBTB2, RHOF, RICTOR, RIPK1, RND1, RNF115, RPL17, RPS6KA3, RSPH1, RUSC2, S100A13, S100A16, S100A6, SART1, SCPEP1, SEC24A, SEC24B, SERPINB8, SGK1, SH2B2, SIPA1, SMAD6, SMG1, SOCS1, SOD3, SOS1, SPTAN1, SQSTM1, SRGAP2, SRP68, SRRM1, SRXN1, STAT1, STAT3, STK24, STXBP3, SULT2A1, SYDE2, SYN1, SYNJ1, TAB3, TAOK1, TCAP, TCEB2, TESK1, THRA, TIRAP, TJP2, TOLLIP, TOM1, TP53INP1, TP53INP2, TP73, TPM1, TRIM24, TRIM25, TSC1, TUBA1A, TXNRD1, UBAP1, USP9X, VPS36, VPS41, WASF2, WIPF2, WIPI2, XRN1, YKT6, ZFP36, ZNF106
**c2**	aging(GO:0007568)	7.06E-04	ADM, ALDOC, AMH, APOD, CASP2, CCL2, CDKN1A, CNP, CYP1A1, ERCC1, FOS, GSN, HRAS, ID2, JUN, KAT6A, LOXL2, MME, NCF2, RELA, SERPINE1, SIN3A, SLC32A1, SMC6, TERF2, TGFB1, TIMP1, UTRN
**c2**	GTPase activity(GO:0003924)	7.76E-04	AGAP1, ARF3, ARL5B, ARL6, ARL8B, EFTUD1, EHD2, EIF5, GBP2, GEM, GNB5, GNG8, GSPT1, HBS1L, HRAS, IFT27, LSG1, PIK3R1, RAB17, RAB21, RAB22A, RAB26, RAB27B, RAB32, RAB33A, RAB33B, RAB38, RAB3D, RAB7A, RALB, RASD1, RHEB, RHOBTB1, RHOBTB2, RHOF, RND1, RRAD, RRAGB, RRAGC, TRIM23, TUBA1A, TUBB2A, TUBB3

**c2**	ubiquitin-protein ligase activity(GO:0004842)	8.03E-04	BIRC7, BRAP, CBLL1, DTX3L, FBXL3, FBXO10, FBXO3, FBXO4, FEM1A, FEM1B, KLHL21, MDM2, NEDD4L, PELI1, PJA2, RC3H1, RNF115, RNF213, RNF40, RNF43, TNFAIP1, TOPORS, TRIM11, TRIM2, TRIM21, TRIM23, TRIM24, TRIM25, TRIM27, TRIM5, TRIM56, TRIM68, UBE2I, UBE2J1, UBE2O, UBE2Q1, UBE2Z, UBOX5, UHRF2
**c2**	regulation of epidermis development(GO:0045682)	8.05E-04	AQP3, CYP27B1, ERRFI1, HPSE, JUP, KEAP1, KRT17, NAB1, NAB2, NGFR, VDR
**c2**	GTP catabolic process(GO:0006184)	9.68E-04	AGAP1, ARF3, ARL8B, EFTUD1, EIF5, GEM, GNB5, GNG8, GSPT1, HRAS, IFT27, LSG1, RAB17, RAB21, RAB22A, RAB26, RAB27B, RAB32, RAB33A, RAB33B, RAB38, RAB3D, RAB7A, RALB, RASD1, RHEB, RHOBTB1, RHOBTB2, RHOF, RND1, RRAD, RRAGC, TRIM23, TUBA1A, TUBB2A, TUBB3
**c2**	regulation of granulocyte chemotaxis(GO:0071622)	9.97E-04	C3AR1, CCL2, CCL5, PTPRJ, S100A14
**c2**	Schwann cell differentiation(GO:0014037)	1.17E-03	CYP11A1, GPC1, NAB1, NAB2
**c2**	intracellular receptor mediated signaling pathway(GO:0030522)	1.20E-03	AIM2, CALCOCO1, CASP8, CRY1, CYLD, DAXX, ESRRB, KDM3A, NFKBIA, NR1D2, NR1I3, NR2F2, NR3C1, NR4A1, NR6A1, PDK3, PGR, PIAS1, PPARD, PTGER3, RELA, RXRA, STAT3, TAB3, THRA, THRAP3, VDR
**c2**	kinase activity(GO:0016301)	1.24E-03	ACVR1, AMHR2, AXL, BMP2K, BMPR2, BRAF, CALM1, CAMKK2, CASS4, CCL2, CCL5, CCNK, CDK19, CDK20, CDKL3, CDKN1A, CHKA, CLK4, CNP, CRIM1, CSF1R, DAPK3, DGKH, DGUOK, DLG3, DYRK1A, DYRK1B, EFNA4, EIF2AK3, EPHB6, ETNK1, FASTKD2, FASTKD5, FIP1L1, FKBP1A, GALK2, HIPK1, HMGXB3, ICK, IP6K1, IPMK, ITPKC, KSR1, LATS1, LIMK2, LMTK2, MAGI3, MAP3K10, MAP3K12, MAP3K13, MAPK7, MET, MLKL, N4BP2L2, NAGK, NEK7, NLK, NRBP1, OXSR1, PAK4, PASK, PDGFRB, PDK3, PDPK1,

			PDXK, PI4K2A, PI4KA, PIK3R4, PINK1, PIP4K2A, PKLR, PKM, POLR2A, POLR2L, PRKCI, PTK2B, RIPK1, RPS6KA3, SCYL3, SGK1, SGMS2, SLK, SMG1, SQSTM1, STK16, STK24, TAOK1, TBCK, TESK1, TESK2, TEX14, TJP2, TP53RK, TRIB1, TRIM24, TRIM27, TWF1, XYLB
**c2**	lysosomal lumen(GO:0043202)	1.34E-03	ARSA, CTSA, GBA, GM2A, GNS, GPC1, HPSE, HYAL1, IDS, IDUA, NEU1, PDGFRB, SDC2, SDC4, SMPD1
**c2**	lipid binding(GO:0008289)	1.54E-03	ACBD3, AGAP1, ALOX5AP, ALS2, ANLN, ANXA2, ANXA4, ANXA5, ANXA9, APOD, APOL2, APPL1, ARHGAP12, ARHGAP23, ARHGAP25, ARHGEF18, ARHGEF19, ARL6, ATP5G3, AXL, BDH1, BIN2, CLVS1, CPNE1, CYP11A1, DAPP1, DGKH, DOCK9, ESRRB, F3, FGD6, FNBP1L, GRB10, HNF4A, IRS2, NGEF, NPC2, NR2F2, NR3C1, OSBPL7, PAQR5, PASK, PCLO, PCYT1A, PGR, PHLDA1, PIGU, PIK3R1, PLD1, PLEKHA1, PLEKHF2, PLEKHM1, PPARD, PRKCI, RALGPS2, RAPGEF2, RAPH1, RASA4, RASGRP3, RHO, RPH3A, S100A13, S1PR1, S1PR3, SGK1, SH2B2, SHBG, SNX11, SNX30, SNX7, SOS1, STARD3, STX3, SWAP70, SYTL4, TBC1D2, TIRAP, TWF1, VEPH1, VIL1, VPS36, WDR45B, WIPI2, ZCCHC14, ZFYVE1, ZFYVE26, ZFYVE9
**c2**	vesicle-mediated transport(GO:0016192)	1.83E-03	ADM, ALS2, ANK1, AP1G1, AP1G2, AP1S2, AP1S3, AP5Z1, ARC, ARF3, ARFGEF2, ATP6V1H, AXL, BCAP29, BRPF3, CALCRL, CALM1, CALY, CCL5, CD9, CEBPE, CHMP4C, CHMP7, CLIP2, CLN3, CLTA, COPB1, CPNE1, CREB3L2, DOPEY2, DPYSL2, EHD2, ELMOD2, EXOC2, EXOC3L2, FAM160A2, FLNA, FNBP1L, FOLR1, GOLGA4, GOSR1, GOSR2, GSN, HGS, HRAS, KIAA0226, KIF13A, LAMP2, LDLR, LLGL2, LMTK2, LRP4, MIA3, MICAL3, MYO7A, NRBP1, OPTN, PCLO, PI4K2A, PRKCI, RAB17, RAB22A, RAB26, RAB27B, RAB3D, RAB7A, RILP, RIN1, S100A13, SEC24A, SEC24B, SERPINE1, SNAP29, SQSTM1, STON2, STX1A, STX3, STXBP3, SYNJ1, SYNRG, SYTL4,

			TGFB1, TIMP1, TNFAIP2, TOM1, TRAK1, TRAPPC10, TXLNA, VPS18, VPS36, VPS37B, VPS37C, VPS41, WASF2, YKT6, ZFYVE9
**c2**	alanine transmembrane transporter activity(GO:0022858)	1.92E-03	SLC1A4, SLC36A1, SLC38A7
**c2**	cytoplasmic mRNA processing body(GO:0000932)	1.94E-03	AGO2, CCRN4L, EDC4, MEX3A, MEX3B, PATL1, PSMC2, RC3H1, SQSTM1, TRIM21, TRIM5, WTIP, XRN1
**c2**	response to hyperoxia(GO:0055093)	2.08E-03	CDKN1A, CYP1A1, EGR1, NCF2, PDGFRB, TXNRD1
**c2**	myeloid dendritic cell differentiation(GO:0043011)	2.08E-03	BATF, BATF2, BATF3, RELB, TGFB1, UBD
**c2**	embryo implantation(GO:0007566)	2.08E-03	GRN, MST1, NLRP5, PPARD, RXRA, SCGB1A1, TEAD4, TRO, UBTF, VMP1
**c2**	response to fibroblast growth factor stimulus(GO:0071774)	2.12E-03	CCL2, CCL5, CD44, CYP11A1, HYAL1, NR4A1, SCGB1A1
**c2**	potassium ion binding(GO:0030955)	2.19E-03	KCNJ11, PDXK, PKLR, PKM
**c2**	SMAD protein import into nucleus(GO:0007184)	2.19E-03	JUN, TGFB1, TOB1, ZFYVE9
**c2**	SMAD protein complex assembly(GO:0007183)	2.19E-03	EID2, FKBP1A, TGFB1, ZFYVE9
**c2**	GDP binding(GO:0019003)	2.49E-03	ARL8B, GEM, RAB17, RAB21, RAB22A, RAB27B, RAB7A, RHEB, RRAGC, TRIM23
**c2**	ferric iron transport(GO:0015682)	2.54E-03	ATP6V0D1, ATP6V0D2, ATP6V1B2, ATP6V1C1, ATP6V1D, ATP6V1E1, ATP6V1H, LMTK2, TFRC
**c2**	multi-organism process(GO:0051704)	2.57E-03	ACP5, ACTA2, ADM, AP1G2, APLP2, APOD, ATP6V0D1, ATP6V0D2, ATP6V1B2, ATP6V1C1, ATP6V1D, ATP6V1E1, ATP6V1H, BAIAP2, BATF,

			BATF2, BATF3, CASP8, CCL2, CCL5, CEBPE, COL16A1, COPB1, CREB3, CREBBP, CRTC2, CYP11A1, CYP1A1, DAXX, DDB1, DDX58, DEFB1, DRD5, EFNB2, ELMOD2, ETS1, F2RL1, FOS, HIST2H2BE, HRAS, HYAL1, IER3, IFI16, IFIT3, IFNGR1, IL2RB, IL6R, IRF7, ISG20, JUN, KCTD5, KRT19, KRT8, LDLR, LTBR, MAGI3, MDM2, MICA, MICB, MMP1, MPDZ, MPO, NCF2, NEDD4L, NFKBIA, NFX1, NPC2, NUP98, OAS3, PAPPA, PGF, PGLYRP4, PIK3R1, PKLR, PLAC8, PLD1, PPM1D, PRG2, PSMC2, RELA, RLF, RXRA, S100A14, SCGB1A1, SERPINE1, SLC25A4, SLFN11, SP110, STAT1, STAT3, SYNCRIP, TAC3, TAF4, TFRC, TGFB1, TIRAP, TNFRSF1A, TNIP1, TRIM11, TRIM23, TRIM25, TRIM5, TRIM56, UBE2I, UBN1, VIL1, XPR1, ZC3HAV1, ZMYND11
**c2**	midbody(GO:0030496)	2.58E-03	ANXA2, ARL8B, CHMP4C, CYLD, FLCN, IST1, KEAP1, KIF13A, PITPNM1, RALB, SLC2A1, SPG20, SSH1, TEX14, TOPORS, VPS37B, ZFYVE26
**c2**	phosphorus metabolic process(GO:0006793)	2.82E-03	ACP5, ACVR1, ADAM10, AMHR2, ATP5D, AVPI1, AXL, BIRC7, BMP2K, BMPR2, BRAF, C5, CAMKK2, CCL2, CCL5, CDK19, CDK20, CDKL3, CLK4, CRK, CSF1R, DAPK3, DAPP1, DAXX, DGUOK, DUSP1, DUSP13, DUSP3, DUSP5, DYRK1A, DYRK1B, EFNA1, EIF2AK3, EPHB6, FIP1L1, FNIP2, FRS2, GADD45B, GALK2, HIPK1, HMGXB3, HRAS, HUS1, ICK, INPP5J, IP6K1, KSR1, LATS1, LIMK2, LMTK2, MAP3K10, MAP3K12, MAP3K13, MAPK7, MET, MEX3B, MLKL, MOB1B, MTMR11, NEK7, NLK, NRBP1, OXSR1, PAK4, PASK, PDGFRB, PDK3, PDPK1, PI4KA, PIK3R1, PIK3R4, PINK1, PIP4K2A, POLR2A, POLR2L, PPAPDC1B, PPM1A, PPM1B, PPM1D, PPP3CA, PRKCI, PTK2B, PTPN23, PTPN6, PTPRF, PTPRJ, PXN, RHO, RIPK1, RPRD1A, RPS6KA3, RSRC1, SCYL3, SGK1, SLC20A1, SLK, SMG1, SSH1, SSH2, STK16, STK24, SYNJ1, TAB3, TAOK1, TBCK, TESK1, TESK2, TEX14, TGFB1, TOLLIP, TP53RK, TP73, TRIB1, TRIM24

**c2**	positive regulation of angiogenesis(GO:0045766)	2.90E-03	ADM, C3, C3AR1, C5, ECM1, ETS1, F3, HIPK1, HYAL1, IL1A, PGF, PTK2B, RUNX1, SERPINE1, TNFRSF1A, VASH2
**c2**	vacuolar transport(GO:0007034)	3.49E-03	CHMP7, CLN3, DSCR3, FAM160A2, HGS, HGSNAT, KIF13A, RAB7A, RILP, TRAK1
**c2**	ruffle organization(GO:0031529)	3.53E-03	ARFIP2, CSF1R, LPIN1, PLEKHA1, TPM1
**c2**	response to tropane(GO:0014073)	3.74E-03	DPYSL2, DRD5, EGR1, HDAC5, HNMT, MDM2, PTK2B, SMPD1
**c2**	regulation of bone mineralization(GO:0030500)	3.81E-03	ACVR1, ATRAID, BMP2K, BMPR2, CD276, CYP27B1, ECM1, LTBP3, MEF2A, NBR1, PTK2B, TGFB1
**c2**	glycosaminoglycan catabolic process(GO:0006027)	3.81E-03	CD44, GNS, GPC1, HGSNAT, HPSE, HYAL1, IDS, IDUA, PGLYRP4, SDC2, SDC4, TGFB1
**c2**	regulation of I-kappaB kinase/NF-kappaB cascade(GO:0043122)	4.33E-03	C9orf89, CARD8, CASP8, ECM1, F2RL1, FKBP1A, FLNA, LTBR, PDPK1, PELI1, PELI2, PINK1, PPM1A, RELA, RIPK1, S100A13, SLC20A1, SQSTM1, STAT1, TIRAP, TNFRSF1A, TNIP1, TRIM38, TRIM5, UBD, ZC3HAV1, ZMYND11
**c2**	B cell differentiation(GO:0030183)	4.36E-03	BCL6, CLCF1, DCLRE1C, FNIP1, FOXP1, HDAC5, IL11, ITGA4, LRRC8A, PIK3R1, PTK2B, ZBTB1
**c2**	heart valve morphogenesis(GO:0003179)	4.41E-03	ACVR1, CYR61, EFNA1, JUP, MEF2A, STRA6, ZFPM1
**c2**	plus-end-directed organelle transport along microtubule(GO:0072386)	4.51E-03	KIF13A, KIF3A, KIF3B
**c2**	positive regulation of toll-like receptor 4 signaling pathway(GO:0034145)	4.51E-03	F2RL1, PELI1, TIRAP
**c2**	AP-1 adaptor complex(GO:0030121)	4.51E-03	AFTPH, AP1G2, SYNRG

**c2**	Ran guanyl-nucleotide exchange factor activity(GO:0005087)	4.51E-03	ALS2, RANBP10, RCBTB2
**c2**	protein binding transcription factor activity(GO:0000988)	4.64E-03	ASXL1, ATF6, BATF3, BCL9L, CALCOCO1, CBFA2T2, CIR1, CREB3, CREBBP, CRTC2, CRY1, CRYM, DMAP1, DYRK1B, ESRRB, GPS2, HDAC5, HSF2, JMY, JUN, JUP, KAT6A, KLF4, LOXL2, LPIN1, MAP3K10, MBD1, MED10, MED15, MED26, MEF2A, MTF1, MXD1, NAB2, NR1I3, NR2C2, NR2F2, PER1, PER2, PIAS1, PRMT2, RAD54L2, RELB, RXRA, SAP130, SERTAD2, SIN3A, SKI, SKIL, SP4, TAF4, TBL1Y, THRAP3, THRB, TOB1, TP53BP1, TRIM24, WTIP, ZFX, ZMIZ2, ZNF136
**c2**	ruffle(GO:0001726)	4.65E-03	ALS2, ARFIP2, BAIAP2, BCAR1, CD2AP, FGD6, GSN, INPP5J, KLHL41, RAB22A, S100A6, SH2B2, SLC9A3R1, VIL1, WASF2
**c2**	wound healing(GO:0042060)	4.78E-03	ARHGEF19, CD44, DRD5, GSN, HPSE, MIA3, PDGFRB, PLAT, PPARD, SDC2, SPRR3, TGFB1, TIMP1, TPM1
**c2**	adipose tissue development(GO:0060612)	4.93E-03	BDH1, CREB5, FTO, ID2, PPARD, SPG20

Supplementary Table S2: Pathway and network associated with downregulated DEGs of 8-OHD-treated samples (H1 and H5) via MetaCore platform.

#	Maps	pValue	Network Objects from Active Data
**1**	Development_Transcription regulation of granulocyte development	1.83E-07	PU.1, STAT3, CD45, PKC, E2F1, SOCS3, c-Myb, STAT5, G-CSF receptor, Lysozyme, c-Myc, AML1 (RUNX1)
**2**	Prolactin/ JAK2 signaling in breast cancer	6.51E-06	STAT3, STAT5A, CISH, c-Myb,

			Cyclophilin A, STAT5, Cyclin D1, SK4/IK1, STAT5B
**3**	IL-6 signaling in colorectal cancer	8.24E-06	STAT3, Cyclin D2, Cyclin B, Cyclin E, USP7, SOCS3, IL-6, Cyclin D1, Cyclin B1, HSP70, c-Myc
**4**	Cell cycle progression in Prostate Cancer	1.44E-05	STAT3, 4E-BP1, STAT5A, Cyclin B, WNT, E2F1, IL-6, Cyclin D1, AKT(PKB), STAT5B, p38 MAPK
**5**	Regulation of microRNAs in colorectal cancer	1.70E-05	STAT3, LIN-28B, microRNA 18a, EGFR, miR-18a-5p, PTP4A3, MECOM, microRNA 19a, miR-19a-3p, microRNA 20a, AKT(PKB), HGF receptor (Met), c-Myc, miR-20a-5p
**6**	Immune response_IL-4-induced regulators of cell growth, survival, differentiation and metabolism	1.70E-05	STAT3, CPT-1A, CISH, Cyclin E, SOCS3, Cyclin D, MCM6, STAT5, ACADM, Osteoprotegerin, AKT(PKB), SK4/IK1, c-Myc, LPL
**7**	Role of metalloproteases and heparanase in progression of pancreatic cancer	1.77E-05	Alpha 1-antitrypsin, MMP-15, MMP-1, EGFR, MMP-14, VEGF-A, SOS, MMP-11, Collagen IV, CD147
**8**	Influence of multiple myeloma cells on bone marrow stromal cells	1.77E-05	FGFR3, CD80, ITGA4, HES1, IL-6, VEGF-A, FGF2, Jagged2, Osteoprotegerin, IGF-1
**9**	Development_PIP3 signaling in cardiac myocytes	1.81E-05	HGF, 4E-BP1, PARD3, Cyclin D, SOS, AKT(PKB), IGF-1, HGF receptor (Met),

			c-Myc, PARD6, p90Rsk, PI3K cat class IB (p110-gamma)
**10**	Immune response_M-CSF-receptor signaling pathway	1.88E-05	PU.1, STAT3, Syk, Cyclin D2, STAT5A, PKC, ETS1, YES, Cyclin D1, AKT(PKB), SOS1, GRAP2, c-Myc, STAT5B, PLAU (UPA), IP3 receptor
**11**	Role of IGH translocations in multiple myeloma	1.88E-05	Cyclin D2, FGFR3, Histone H3, HDAC2, HES1, E2F1, VEGF-A, Cyclin D1, IRF4, c-Myc, Histone H4
**12**	c-Myc in multiple myeloma	2.20E-05	STAT3, GRP78, IL-6, VEGF-A, IRF4, c-Myc, p38 MAPK
**13**	Signal transduction_Angiotensin II/ AGTR1 signaling via p38, ERK and PI3K	2.36E-05	Syk, ELAVL1 (HuR), 4E-BP1, ALOX12, ETS1, EGFR, MEF2C, MMP-14, SP3, IL-6, G-protein alpha-q, Cyclin D1, SOS, AKT(PKB), p38 MAPK, PI3K cat class IB (p110-gamma), CaMK II delta, ATP7A
**14**	Development_Thrombopoietin signaling via JAK-STAT pathway	4.07E-05	STAT3, STAT5A, CISH, SOCS3, STAT5, Cyclin D1, SOCS2, STAT5B
**15**	Role of histone modificators in progression of multiple myeloma	5.11E-05	Cyclin D2, Tubulin alpha, Histone H3, HDAC2, E2F1, IL-6, Cyclin D1, Jagged2, Histone H4
**16**	Multiple myeloma (general schema)	5.48E-05	FGFR3, IL-6, VEGF-A, Cyclin D1, FGF2, IGF-1, c-Myc
**17**	NETosis in SLE	6.81E-05	PKC, Histone H3, HMGB1, IFN-alpha, Fc gamma RII alpha, Histone H2, Histone

			H2A, p38 MAPK, Histone H4
**18**	Resolution of inflammation in healing myocardial infarction	8.05E-05	STAT3, HGF, MMP-1, VEGF-A, G-CSF receptor, FGF2, AKT(PKB), HGF receptor (Met)
**19**	Signal transduction_Leptin signaling via JAK/STAT and MAPK cascade	8.69E-05	STAT3, PPCKC, STAT5A, SOD2, SOCS3, STAT5, VEGF-A, Leptin receptor, STAT5B, p90Rsk
**20**	FGF2 signaling in melanoma	8.69E-05	STAT3, FGFR3, MMP-1, YES, VEGF-A, FGF2, SOS, Collagen IV, Fibronectin, p38 MAPK
**21**	Cell adhesion_ECM remodeling	9.66E-05	TIMP3, Caveolin-2, MMP-15, MMP-1, EGFR, MMP-14, Collagen IV, IGF-1, Fibronectin, Kallikrein 1, PLAU (UPA), Kallikrein 3 (PSA)
**22**	ErbB2-induced breast cancer cell invasion	1.78E-04	ITGA4, MMP-1, ETS1, NGAL, EGFR, MMP-14, VEGF-A, SOS, AKT(PKB), ER81, p38 MAPK, PLAU (UPA), IP3 receptor
**23**	Ligand-independent activation of Androgen receptor in Prostate Cancer	1.78E-04	STAT3, STAT5A, NCOA3 (pCIP/SRC3), EGFR, PP2A regulatory, Cyclin D1, FGF2, SOS, AKT(PKB), IGF-1, c-Myc, STAT5B, Kallikrein 3 (PSA)
**24**	Transcription_HIF-1 targets	1.92E-04	Heme oxygenase 1, FECH, Carbonic anhydrase XII, Transferrin, Nucleophosmin, PLGF, REDD1, NOXA, VEGF-A, P4HA1, WT1, FGF2, HGF

			receptor (Met), c-Myc, Stanniocalcin 2, AK3
**25**	Tumor-stroma interactions in pancreatic cancer	1.96E-04	HGF, MMP-1, VEGF-A, FGF2, IGF-1, HGF receptor (Met), Fibronectin, CD147
**26**	Immune response_Sublytic effects of membrane attack complex	2.08E-04	AKT1, GRP78, PKC, EGFR, IL-6, SOS, AKT(PKB), Cyclin B1, RGC32, p38 MAPK, ATF-6 alpha (50kDa), PI3K cat class IB (p110-gamma), IP3 receptor
**27**	Immune response_IL-15 signaling via JAK-STAT cascade	2.37E-04	STAT3, Syk, STAT5A, IL-15RA, STAT5, sIL-15RA, STAT5B
**28**	PR action in breast cancer: stimulation of cell growth and proliferation	2.41E-04	STAT3, STAT5A, Cyclin E, EGFR, Cyclin D1, SOS, c-Myc, PR (membrane), PR (nuclear)
**29**	Abnormalities in cell cycle in SCLC	2.57E-04	Histone H3, CKS1, Cyclin E, E2F1, Cyclin D1, Aurora-B, Cyclin B1, c-Myc
**30**	Mechanisms of resistance to EGFR inhibitors in lung cancer	3.12E-04	HGF, 4E-BP1, Ep-CAM, EGFR, Claudin-7, CIN85, SOS, AKT(PKB), HGF receptor (Met), Fibronectin
**31**	Signal transduction_Adenosine A1 receptor signaling pathway	3.21E-04	STAT3, GLRA3, PKC, EGFR, ASK1 (MAP3K5), Adenylate cyclase, ADA, AKT(PKB), G-protein alpha-q/11, SFK, p38 MAPK, IP3 receptor
**32**	Apoptosis and survival_Granzyme A signaling	3.32E-04	Rab-27A, Histone H3, NDPK A, SET, DNA ligase IV, Lamin B1, PHAP1 (pp32), MUNC13-4

**33**	Transcription_Ligand-dependent activation of the ESR1/SP pathway	3.32E-04	NCOA3 (pCIP/SRC3), Cyclin E, E2F1, EGFR, SP3, ADA, VEGF-A, Cyclin D1
**34**	Immune response_HSP60 and HSP70/ TLR signaling pathway	3.55E-04	CD69, CD80, IRAK4, IL-6, TPL2(MAP3K8), IRAK1/2, E2N(UBC13), HSP60, HSP70, p38 MAPK, IL-12 alpha
**35**	ERBB family and HGF signaling in gastric cancer	3.55E-04	STAT3, HGF, STAT5A, EGFR, VEGF-A, Cyclin D1, SOS, AKT(PKB), HGF receptor (Met), c-Myc, p38 MAPK
**36**	Transcription_Hypoxia- and receptor-mediated HIF-1 activation	3.77E-04	STAT3, HGF, 4E-BP1, NCOA3 (pCIP/SRC3), VHL, EGFR, HIF-prolyl hydroxylase, AKT(PKB), IGF-1, p38 MAPK
**37**	Transcription_Androgen Receptor nuclear signaling	3.77E-04	STAT3, AKT1, WNT, EGFR, IL-6, Cyclin D1, SOS, AKT(PKB), IGF-1, Kallikrein 3 (PSA)
**38**	Immune response_IL-3 signaling via JAK/STAT, p38, JNK and NF-kB	4.89E-04	CD69, PU.1, STAT3, Cyclin D2, STAT5A, CISH, SOCS3, IL-6, STAT5, Cyclin D1, AKT(PKB), Cyclin B1, Fibronectin, c-Myc, p38 MAPK
**39**	Main genetic and epigenetic alterations in lung cancer	5.42E-04	HGF, FHIT, E2F1, EGFR, VEGF-A, Cyclin D1, DNMT3A, IGF-1, HGF receptor (Met), c-Myc
**40**	Signal transduction_Adenosine A3 receptor signaling pathway	5.42E-04	STAT3, PKC, Adenylate cyclase, Rho GTPase, VEGF-A, AKT(PKB), G-protein alpha-q/11, p38 MAPK, PI3K cat class IB (p110-gamma), IP3 receptor

**41**	Immune response_IL-2 signaling via JAK/ STAT	5.68E-04	STAT3, Syk, Cyclin D2, STAT5A, STAT5, c-Myc, STAT5B
**42**	Transcription_Transcription regulation of aminoacid metabolism	5.68E-04	FECH, NF-E2 (45 kDa), PKC, DCOR, ASSY, HMBS, c-Myc
**43**	Tissue Factor signaling in cancer via PAR1 and PAR2	6.44E-04	STAT5A, EGFR, YES, VEGF-A, SOS, AKT(PKB), G-protein alpha-q/11, Tissue factor, STAT5B, IP3 receptor
**44**	Development_Growth factors in regulation of oligodendrocyte progenitor cell proliferation	6.72E-04	HGF, FGFR3, PKC, Tenascin-C, EGFR, Cyclin D1, FGF2, SOS, AKT(PKB), IGF-1, HGF receptor (Met), Fibronectin
**45**	Immune response_IL-15 signaling	6.76E-04	4E-BP1, IL-15RA, AKT1, ETS1, IL-6, sIL-15RA, SOS, AKT(PKB), c-Myc, Rac3, p38 MAPK
**46**	CTP/UTP metabolism	7.62E-04	ITPA, POLR1B, AK2, CTP synthase II, RRP4, RPB5, NDPK A, NDPK complex, RRP46, NDPK D (mitochondrial), POLR2I, NT5M, POLR2J, RRP43, NDPK B, AK3
**47**	ATP metabolism	7.62E-04	ITPA, FHIT, BPNT1, AK2, WARS, Adenylate cyclase type VIII, NDPK A, NDPK complex, NMNA3, Connexin 43, ENPP3, NDPK D (mitochondrial), KPYR, NDPK B, AK3, Adenylate cyclase type III
**48**	Immune response_Fc epsilon RI pathway: Lyn-mediated cytokine production	7.63E-04	Syk, CARD9, Slp76, PKC, MEF2C, IL-6, SOS, FYB1, GRAP2, Btk, p38 MAPK, NF-AT, IP3 receptor, SH3BP-2

**49**	Oxidative stress_ROS-induced cellular signaling	8.44E-04	Syk, ELAVL1 (HuR), Heme oxygenase 1, Chk2, PKC, HES1, DLC1 (Dynein LC8a), IL-6, NOXA, VEGF-A, Cyclin D1, TXNIP (VDUP1), HIF-prolyl hydroxylase, AKT(PKB), Cyclin B1, p38 MAPK
**50**	DNA damage_ATM/ATR regulation of G2/M checkpoint: cytoplasmic signaling	8.97E-04	Chk2, Histone H3, Nucleolysin TIAR, B56G, PP2A regulatory, hnRNP A0, Aurora-B, p38gamma (MAPK12), Cyclin B1, p38 MAPK

Supplementary Table S3: Top KEGG pathways associated with putative targets of 8-OHD against ML

Term	Count	%	*p* Value	Entrez_genes	Bonferroni	Benjamini	FDR
hsa05221:Acute myeloid leukemia	14	13.3	5.22E-15	5294, 5371, 5595, 4893, 6654, 6774, 5291, 4609, 51176, 6777, 6688, 207, 6776, 3845	8.82E-13	8.82E-13	5.37E-13
hsa05220:Chronic myeloid leukemia	12	11.4	9.94E-11	5294, 4609, 5595, 7157, 4893, 6654, 6777, 6776, 207, 3066, 5291, 3845	1.68E-08	7.20E-09	4.39E-09
hsa05161:Hepatitis B	15	14.3	1.28E-10	5294, 6773, 5595, 7157, 4893, 6774, 5291, 332, 3569, 4609, 6777, 207, 6776, 6778, 3845	2.16E-08	7.20E-09	4.39E-09
hsa04151:PI3K-Akt signaling pathway	20	19.0	7.52E-10	5294, 7010, 3791, 5595, 7422, 7157, 4893, 6654, 7533, 3320, 5291, 284, 3569, 4609, 3718, 3479, 2321, 207, 4602, 3845	1.27E-07	3.18E-08	1.94E-08
hsa05200:Pathways in cancer	21	20.0	9.77E-10	5294, 5371, 5595, 7422, 7157, 4893, 6654, 6774, 3320, 3066, 5291, 332, 3569, 4609, 3479, 51176, 6777, 6688, 207, 6776, 3845	1.65E-07	3.30E-08	2.01E-08
hsa05213:Endometrial cancer	10	9.5	1.79E-09	5294, 4609, 5595, 7157, 51176, 4893, 6654, 207, 5291, 3845	3.03E-07	5.05E-08	3.08E-08
hsa05214:Glioma	10	9.5	1.40E-08	808, 5294, 3479, 5595, 7157, 4893, 6654, 207, 5291, 3845	2.37E-06	3.12E-07	1.90E-07
hsa05215:Prostate cancer	11	10.5	1.48E-08	5294, 3479, 5595, 7157, 51176, 4893, 6654, 3320, 207, 5291, 3845	2.50E-06	3.12E-07	1.90E-07

hsa04917:Prolactin signaling pathway	10	9.5	3.11E-08	5294, 5595, 4893, 6654, 6774, 6777, 6776, 207, 5291, 3845	5.26E-06	5.84E-07	3.56E-07
hsa04066:HIF-1 signaling pathway	11	10.5	3.46E-08	5294, 7010, 3569, 3479, 5595, 7422, 2321, 6774, 207, 5291, 284	5.85E-06	5.85E-07	3.57E-07
hsa04014:Ras signaling pathway	15	14.3	4.31E-08	808, 5294, 7010, 3791, 5595, 7422, 4893, 6654, 5291, 284, 3479, 2321, 207, 5923, 3845	7.28E-06	6.62E-07	4.04E-07
hsa04550:Signaling pathways regulating pluripotency of stem cells	12	11.4	1.30E-07	5294, 4086, 4609, 3718, 3479, 5595, 4211, 4893, 6774, 207, 5291, 3845	2.19E-05	1.83E-06	1.11E-06
hsa04015:Rap1 signaling pathway	14	13.3	1.41E-07	808, 5294, 7010, 3791, 5595, 7422, 3683, 4893, 5291, 284, 3479, 2321, 207, 3845	2.38E-05	1.83E-06	1.12E-06
hsa05210:Colorectal cancer	9	8.6	1.67E-07	5294, 332, 4609, 5595, 7157, 51176, 207, 5291, 3845	2.82E-05	1.87E-06	1.14E-06
hsa05166:HTLV-I infection	15	14.3	1.86E-07	5294, 7015, 7157, 3683, 4893, 5291, 3569, 4609, 3718, 6777, 6688, 207, 6776, 4602, 3845	3.14E-05	1.87E-06	1.14E-06
hsa04630:Jak-STAT signaling pathway	12	11.4	1.86E-07	5294, 3569, 4609, 3718, 6773, 6654, 6774, 6777, 6776, 207, 6778, 5291	3.14E-05	1.87E-06	1.14E-06
hsa04012:ErbB signaling pathway	10	9.5	1.88E-07	5294, 4609, 5595, 4893, 6654, 6777, 6776, 207, 5291, 3845	3.18E-05	1.87E-06	1.14E-06
hsa05205:Proteoglycans in cancer	13	12.4	6.35E-07	5294, 3791, 5595, 7422, 7157, 4893, 6654, 6774, 5291, 4609, 3479, 207,	1.07E-04	5.96E-06	3.63E-06

				3845			
hsa05202:Transcriptional misregulation in cancer	12	11.4	7.78E-07	5371, 8013, 3569, 4086, 4609, 3479, 7157, 4211, 2321, 6688, 3066, 4208	1.31E-04	6.92E-06	4.22E-06
hsa05203:Viral carcinogenesis	13	12.4	8.28E-07	5294, 5595, 9734, 7157, 4893, 7533, 6774, 3066, 5291, 3718, 6777, 6776, 3845	1.40E-04	6.99E-06	4.26E-06
hsa05223:Non-small cell lung cancer	8	7.6	1.30E-06	5294, 5595, 7157, 4893, 6654, 207, 5291, 3845	2.20E-04	1.05E-05	6.37E-06
hsa04370:VEGF signaling pathway	8	7.6	2.35E-06	5294, 5595, 7422, 3791, 4893, 207, 5291, 3845	3.97E-04	1.80E-05	1.10E-05
hsa04722:Neurotrophin signaling pathway	10	9.5	2.94E-06	808, 5294, 5595, 7157, 4893, 6654, 207, 4217, 5291, 3845	4.98E-04	2.16E-05	1.32E-05
hsa05230:Central carbon metabolism in cancer	8	7.6	3.26E-06	5294, 4609, 5595, 7157, 4893, 207, 5291, 3845	5.51E-04	2.30E-05	1.40E-05
hsa05212:Pancreatic cancer	8	7.6	3.63E-06	5294, 5595, 7422, 7157, 6774, 207, 5291, 3845	6.13E-04	2.45E-05	1.49E-05

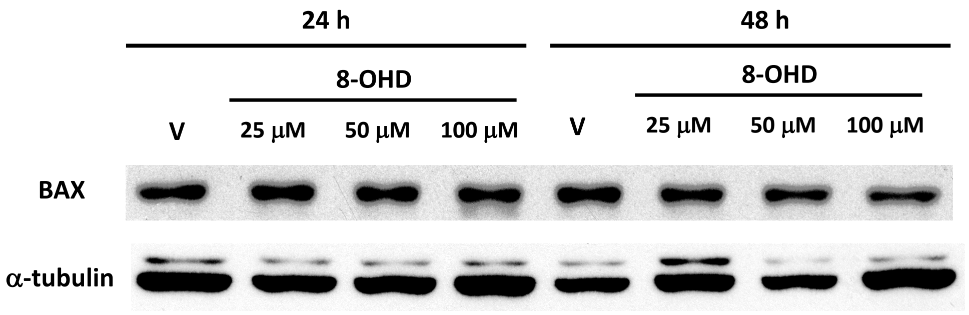

Supplementary Figure S1. Western blotting analysis of BAX expression in response to 8-OHD treatment.
